# Supplementary material for: A weakly structured stem for human origins in Africa
Source: Nature. 2023 May 17;617(7962):755–63. doi: 10.1038/s41586-023-06055-y (PMC10208968; doi:10.1038/s41586-023-06055-y)
Supplement: Supplementary file 1 — This file contains further discussion, methods and data, Supplementary Tables S1–S8, Supplementary Figs. S1–S40, and Supplementary References. [file 41586_2023_6055_MOESM1_ESM.pdf]

nature portfolio

https://doi.org/10.1038/s41586-023-06055-y

Supplementary information

A weakly structured stem for human origins in Africa

In the format provided by the authors and unedited

Supplementary Information for "A weakly structured stem for human origins in Africa"

Aaron P. Ragsdale, Timothy D. Weaver, Elizabeth G. Atkinson, Eileen Hoal, Marlo Möller, Brenna M. Henn, and Simon Gravel

March 16, 2023

Contents

1 Data and sequencing **2**
1.1 Sequencing and variant calling . . . 2
1.1.1 ADMIXTURE . . . 2
1.2 Nama sample collection and consent . . . 3
1.2.1 Ethics and Initiation of DNA Collection . . . 3
1.2.2 Collection Procedures . . . 3
1.2.3 Return of Results . . . 3
1.3 Details about populations used in analyses . . . 4
1.4 Filtering and subsetting data . . . 4
1.5 PCA and `ADMIXTURE` analysis . . . 4

2 Computing statistics **5**
2.1 LD and diversity statistics used in model fits . . . 5
2.2 Computing LD and diversity statistics . . . 5
2.3 Estimating two-locus statistics with finite sample sizes . . . 6
2.4 Computing conditional SFS . . . 6

3 Model specification and fitting **7**
3.1 General strategy for building models and introducing complexity . . . 8
3.1.1 Evidence for the Out of Africa timing . . . 8
3.2 Likelihoods, uncertainty, and confidence intervals . . . 10
3.3 Optimization using `moments` . . . 10

4 Gene genealogy reconstruction **10**

5 Predictions from inferred demographic models **11**
5.1 Divergence between coexisting populations over time . . . 11
5.2 Shared differentiation between pairs of contemporary and pairs of ancient populations . . . 12
5.3 Back-to-Africa Gene flow and Neanderthal ancestry in Africa . . . 12
5.4 Relating model predictions to morphological variation . . . 13

6 Model validation strategy **14**
6.1 Validations using alternative data sets and model specification . . . 14
6.1.1 Alternate Thousand Genomes population sets . . . 14
6.1.2 Variable thresholds of Nama-associated ancestry . . . 14
6.1.3 Varying fixed divergence times . . . 15
6.1.4 Recombination map inferred from Nama . . . 15
6.2 Validations using simulations from inferred demographic models . . . 15
6.2.1 Simulation details . . . 15
6.2.2 cSFS prediction under inferred models . . . 16

7 **Model validation results** **16**
7.1 Model fits using alternative datasets . . . 16
7.1.1 Replacing Thousand Genomes populations . . . 16
7.1.2 Fit to LD statistics from Nama recombination map . . . 17
7.1.3 Varying Nama ancestry-proportion thresholds . . . 17
7.2 Relaxed inference of divergence times . . . 17
7.3 Alternative statistics for four models . . . 18
7.3.1 Conditional SFS . . . 18
7.3.2 Relate curves from inferred models . . . 18
7.3.3 Distribution of deep branch affinities to Neanderthal sequence . . . 19
7.4 Re-inference of IM models from simulated data . . . 19
7.5 Evaluation of estimated confidence intervals . . . 20
7.6 Batch effects from merged data biases inference . . . 21
7.7 Model choice from likelihood differences . . . 22
7.8 Allowing for additional migrations . . . 23
7.9 Replacing the reference population for statistics normalization . . . 24

8 **Mutation versus recombination rates** **24**

References **24**

Supplementary tables and figures **29**

1 Data and sequencing

1.1 Sequencing and variant calling

Low coverage (4-8x) Illumina short read data were generated for the Nama, Gumuz, Amhara and Oromo populations as part of the African Diversity Reference Panel (Sanger / Wellcome Trust) (Gurdasani *et al.*, 2015; Pagani *et al.*, 2015) and approved through a secondary data analysis agreement for this project. Briefly, raw reads were aligned to GRCH37 with BWAmem, duplicates marked with Picard MarkDuplicates, reads were realigned around indels with GATK RealignerTargetCreater/IndelRealigner followed by BQSR with dbSNP 137. Contamination checks were performed requiring that FREEMIX < 0.05; contamination checks resulted in the elimination of 22 Nama samples. We note that the high heterozygosity in these genomes both due to inherent genetic diversity and admixture may have violated base assumptions for this heterozygosity check. Genomes were then variant called with GATK3.2 Unified Genotyper (DePristo *et al.*, 2011) using joint calling across 2,478 individuals within the African Diversity Reference Panel (ADRP) dataset with a minimum base quality of 17. Data were merged with 1000 Genomes Phase 3 (1000 Genomes Project Consortium *et al.*, 2015) using the union of sites identified with bcftools isec (-n+1) (Danecek *et al.*, 2021) then refined with Variant Recalibrator with a truth sensitivity threshold of 99.5%. HapMap III and dbSNP 138 served as known sites while 1000 Genomes Phase 1 Omni2.5 and Phase 1 genomic SNP served as the training set. After VR, no batch effects were observed along PC1 and PC2 for 1000 Genomes vs. ADRP. Phasing on the combined dataset was performed via SHAPEIT2 (Delaneau *et al.*, 2013) and utilized the duoHMM option for duos and trios. We present new whole-genome data for 82 Nama individuals available under accession number EGAD00001006198. Second- and third-degrees relatives were inferred from reconstructed pedigrees with Omni2.5 SNP array data. Among these 82 Nama samples, we down-sampled the individuals to minimize close relatives and admixture, such that 44 Nama genomes were retained.

1.1.1 ADMIXTURE

Nama individuals with $> 70\%$ estimated Khoe-San ancestry were retained for analysis here, after partitioning ancestry into $K = 6$ clusters with an `ADMIXTURE` (Alexander *et al.*, 2009) analysis on the full ADRP dataset (van Eeden *et al.*, 2022). The 27 ADRP populations represent a wide variety of West African, East African, North African and Bantu-speaking South Africans. Europeans (1000 Genomes: CEU) were included to account for colonial admixture. A Khoe-San specific ancestry emerged at $K = 3$, predominant in the Nama. This ancestry remained stable at higher K's. At $K = 6$, an ancestry representing the southern Bantu-speakers

(Sotho and Zulu) also emerged, at low frequency in some Nama. Higher K's highlighted population-specific ancestries or those with no representation in the Nama, therefore we focused on $K = 6$ for selection of high Khoe-San ancestry individuals. For illustration, `ADMIXTURE` analysis in Figures 2D and S40 was restricted to 1,157 individuals representing a subset of the full ADRP and several 1000 Genomes populations used in analyses presented here (see Section 1.3).

1.2 Nama sample collection and consent

1.2.1 Ethics and Initiation of DNA Collection

DNA samples were collected from three Nama communities in the Richtersveld region of South Africa, which borders southern Namibia. Members of the Nama community in the Richtersveld were initially approached regarding a genetic ancestry study in 2011. The members included church elders, representatives at the Richtersveld National Park including the appointee for Peoples and Conservation (from which we obtained permission to sample individuals who still live in the park), the Richtersveld Cultural World Heritage Site Coordinator, and the Kuboes tourist office. All of these individuals self-identified as Nama. There was strong support for research that examined the extent to which the Nama are related to other Khoe-San populations, the impact of colonial migration, the time depth of their presence in southern Africa and relationships to other African groups. One primary concern was to make sure the research results were reported back to the community. Based on the positive response we received, permission was granted from the Stellenbosch University HREC to continue the study. Written consent was recorded per our IRB protocol with human subjects approval from Stanford University (Protocol #13829), Stellenbosch University (N11/07/210) and later maintained via SUNY Stony Brook (Protocol #727494). DNA were collected for the express purpose of understanding Nama population history, their relationship to other Africans and human evolution. No commercial activities are allowed. The IRB has approved the data for public deposition and sharing provided that the research is related to population history or human evolution.

1.2.2 Collection Procedures

Demographic and DNA collection was initiated in 2012 with a joint team of South African and American researchers which included both geneticists and anthropologists. Research assistants were fluent in Afrikaans, Nama and English. Collection primarily occurred at home, by first approaching a family member, gauging interest in the study, entering the home or sitting outside by their invitation, oral and written consent occurring with family members present and then finally – completing the DNA sampling. By sampling individuals at home, members of the family are able to voice concerns, whether or not they decide to participate, and thus the final decisions are made in a slow and ethical manner. This process sometimes involved introducing the study and then returning at a later day in order to give participants sufficient time to consider. Saliva samples were extracted from Oragene [OGR-500] kits at Stellenbosch University. A copy of the English consent form is available as a Supplementalal Document.

1.2.3 Return of Results

Results stemming from genetic analyses have been communicated in 2015, 2019 and 2021 via community presentations, a radio interview and in person to representatives of the Richtersveld National Park. We created a poster illustrating Khoe-San ancestry proportions, discriminating ancestry on maternal vs. paternal lineages, the relationship between the Nama and Kalahari Khoe-San populations, and the evolution of light skin pigmentation in southern Africa. The poster was printed in Afrikaans and presented to the tourism office in Kuboes (a central location) for display. A display panel on genetic ancestry is currently under construction for the Richtersveld National Park entrance, created in consultation with the authors here. Future community meetings are anticipated in 2023. We remain committed to upholding this decade long relationship with the Nama, and value the community's input in the research process.

1.3 Details about populations used in analyses

From the merged dataset of the African Diversity Reference Panel (ADRP) (Gurdasani *et al.*, 2015; Pagani *et al.*, 2015) and the 1000 Genomes Project Consortium *et al.* (2015) phase 3 data, we subsampled to the populations included in this study. From the 1000 Genomes data, these included all populations in the African super-population (ESN, GWD, LWK, MSL, and YRI) and four non-African populations (CEU, GBR, CHB, and PJL). From the ADRP, these included the Amhara, Bakiga, Gumuz, Nama, Oromo, Somali, and Zulu. This larger set of populations were used in the PCA and `Admixture` analyses. The `Relate` analysis used a slightly smaller set, excluding the Bakiga, Somali, and Zulu samples.

For the demographic modeling, we further subset to the regional populations, from West Africa: Mende from Sierra Leone (MSL); South Africa: Nama from South Africa (newly sequenced); East Africa: Gumuz (traditionally hunter-gatherer with low levels of Eurasian admixture), Oromo and Amhara (combined as traditionally agriculturalists with large proportion of back-to-Africa Eurasian ancestry); and Europe: British (GBR). These were combined with the high-coverage Vindija Neanderthal genome (Prüfer *et al.*, 2017), resulting in 290 total samples used in demographic inference.

The number of publicly available whole-genome sequenced individuals from a diverse set of African groups has grown in recent years (e.g., Mallick *et al.*, 2016; Bergström *et al.*, 2020; Schlebusch *et al.*, 2020). Our choice of populations was motivated by both technical and study design considerations. First, while the diversity and linkage disequilibrium statistics used in our inferences are robust to low coverage sequencing data (Ragsdale and Gravel, 2019), we wanted to have at least 20 individuals per population to increase accuracy in statistical measurements by reducing the variance due to small sample sizes (Ragsdale and Gravel, 2020). Second, merging of datasets post-variant calling can introduce biases in demographic inference due to batch effects (as demonstrated in Section 7.6). We therefore wanted to be able to perform joint calling in all of our samples. Together, these considerations make the jointly called Thousand Genomes and ADRP dataset best-suited for our demographic inference.

From a study design perspective, our choice of populations aimed to improve our understanding of early human demography in Africa. The Nama derive ancestry from a relatively deeply diverged population branch, and thus their demographic history is of unique interest. Given previous archaeological and genetic evidence, we expected that a minimal model for the Nama must include pastoralist East African groups that contributed to the Nama (e.g., Uren *et al.*, 2016), as well as European colonial admixture. The inclusion of three Ethiopian populations here represent some uncertainty in the possible source population for the pastoralist contribution to the Nama, and the Amhara and Oromo also have substantive "back-to-Africa" ancestry. The Gumuz serve as a representative of southwest Ethiopian ancestry that pre-dates the Holocene back-to-Africa migration. Finally, because previous studies have suggested a role for "ghost" archaic admixture or deep structure in West African populations (e.g., Speidel *et al.*, 2019; Durvasula and Sankararaman, 2020), we chose to also include representatives of West African ancestry.

1.4 Filtering and subsetting data

All analyses presented in this work focus on biallelic single nucleotide polymorphisms within the 1000 Genomes Phase 3 strict mask ($L_{included} = 2,064,554,803$bp). To enable comparison with Neanderthal DNA, we excluded regions for which the Vindija Neanderthal sample had less than 100 contiguous base pairs with high quality variant calls ($L_{included} = 1,519,643,507$bp). For the `moments-LD` analysis, we further focused on intergenic regions because these appear less affected by natural selection compared to both synonymous and nonsynonymous variation (Ragsdale *et al.*, 2018) ($L_{included} = 637,639,065$bp).

The 290 individuals used in the `moments-LD` inference featured 18,461,915 polymorphic biallelic SNPs genome-wide in the callable regions with Neanderthal alignment, of which 8,115,115 were in intergenic and thus putatively neutral regions.

1.5 PCA and `ADMIXTURE` analysis

We used principal component analysis (PCA) implemented in `scikit-learn` (Pedregosa *et al.*, 2011) and `ADMIXTURE` version 1.3.0 (Alexander and Lange, 2011) to summarize genetic diversity among a broader set of 1000 Genomes and ADRP populations (Figure 2 in the main text, and Figure S40). Data filtering was identical for both analyses. We retained biallelic SNPs with no missing data and minor allele frequency

greater that 5%. We then thinned the remaining sites to remove SNPs in high linkage disequilibrium with one another. We used the `locate_unlinked` function from `scikit-allel` version 1.2.1 (Miles *et al.*, 2021) to identify SNPs in low linkage disequilibrium, using a threshold of $r^2 = 0.1$, a window size of 100 SNPs, and a step size of 20 SNPs, removing the remaining SNPs. This process was iterated five times for each chromosome, resulting in 327,656 roughly unlinked SNPs used in the PCA and `ADMIXTURE` analyses.

2 Computing statistics

2.1 LD and diversity statistics used in model fits

We used multi-population linkage disequilibrium (LD) and pairwise diversity statistics to fit demographic models to data. These statistics, introduced and described in detail in Ragsdale and Gravel (2019), are the multi-population analog of the classic LD statistics first described and computed by Hill and Robertson (1968); Ohta and Kimura (1971).

Given two biallelic loci, with alleles A and a and the left locus and alleles B and b at the right locus, the standard covariance measure of LD is $D = p_{AB}p_{ab} - p_{Ab}p_{aB}$, where p_{AB} is the frequency of AB haplotypes in a population (and thus the probability of drawing an AB haplotype in a random sample of that population). Hill and Robertson (1968) solve for the expectation of D^2 using a system of equations that includes $\mathbb{E}[Dz] = \mathbb{E}[D(1-2p_A)(1-2p_B)]$ and $\mathbb{E}[\pi_2] = \mathbb{E}[p_A(1-p_A)p_B(1-p_B)]$, where p_A and p_B are the frequencies of A and B at the left and right loci, respectively. This system also requires the expected pairwise diversity (or expected heterozygosity, assuming random mating), denoted $\mathbb{E}[H] = \mathbb{E}[2p_A(1-p_A)] = \mathbb{E}[2p_B(1-p_B)]$, assuming equal mutation rates at the two loci.

Ragsdale and Gravel (2019) showed how to compute the analogous multi-population set of LD statistics, and we refer readers there for details on their definitions, computation, and interpretations. In short, we obtain expectations of D^2 in each population, the cross-population product D_iD_j (where i and j index populations), as well as those additional statistics Dz and π_2 taken over different combinations of population indexing. That is $Dz_{i,j,k} = D_i(1-2p_{A,j})(1-2p_{B,k})$ and $\pi_{2;i,j,k,l} = p_{A,i}(1-p_{A,j})p_{B,k}(1-2p_{B,l})$. We consider statistics normalized by π_2 in an arbitrary reference population (throughout, we use the Mende π_2), which removes any dependence on the mutation rate. Thus, statistics take the form

$$\begin{aligned} \sigma^2_{D;i,j} &= \frac{\mathbb{E}[D^2_{i,j}]}{\mathbb{E}[\pi_2]} \\ \sigma_{Dz;i,j,k} &= \frac{\mathbb{E}[Dz_{i,j,k}]}{\mathbb{E}[\pi_2]}, \end{aligned} \tag{S1}$$

and so on. Multi-population pairwise diversity statistics $\mathbb{E}[H_i] = \mathbb{E}[2p_i(1-p_i)]$ and $\mathbb{E}[H_{i,j}] = \mathbb{E}[p_i(1-p_j) + p_j(1-p_i)]$ were also normalized by H in the Mende, so that all pairwise diversity measures are relative to the reference population.

σ_{Dz} has been shown to be sensitive to deep population structure and archaic hominin admixture (Ragsdale and Gravel, 2019), and this statistic is closely related to S^* statistics used to scan for introgressed haplotypes (Plagnol and Wall, 2006). Pairwise diversity statistics $H_{i,j}$ have also been widely used in demographic inference involving ancient DNA and many statistics, as f_2, f_3 and f_4 statistics can be expressed as linear combinations of $H_{i,j}$. f-statistics form the basis of admixture graph analysis (Lipson, 2020). Therefore, the set of statistics used here encompass multiple features of genetic data that have been used to infer models of archaic hominin admixture and population structure involving many populations.

2.2 Computing LD and diversity statistics

We compared single- and two-locus statistics in the data to predictions based on detailed demographic models. Model predictions were obtained using recursions described in Ragsdale and Gravel (2019) and implemented in the software `moments` (`https://bitbucket.org/simongravel/moments/src/main/`). The model computes expected patterns of single-nucleotide pairwise diversity and linkage disequilibrium as a function of recombination distance between variants within and across populations, under the assumption of neutrality.

For numerical convenience, observed genetic variants were binned by recombination distances. We assessed the robustness of the statistics to errors in the recombination maps by considering three different recombination maps, the OMNI YRI and HapMapII (1000 Genomes Project Consortium *et al.*, 2015; International HapMap Consortium *et al.*, 2007), and a recombination map inferred from the Nama (van Eeden *et al.*, 2022). Statistics were largely unchanged by using a different map (Figures S7, S8). The OMNI and HapMap recombination maps resulted in LD statistics that were nearly identical, with only minor deviations between the OMNI/HapMap and the Nama-inferred recombination maps. Both the OMNI and HapMap maps were inferred using array data, which is sparser than whole-genome sequencing data as was used in the Nama map inference. In subsequent analyses, we used data based on recombination rates from the OMNI YRI map, and in the Supplemental Results we show that the inferences are robust when using the Nama recombination map (Section 7.1).

We removed bins of recombination distance less than a recombination distance of $r = 5 \times 10^{-6}$ (at a rough estimate of 1 cM/Mb, this corresponds to a minimum distance of 500 bp on average) to avoid previously reported biases at short distances due to processes like multinucleotide mutations (Harris and Nielsen, 2014; Ragsdale and Gravel, 2019). To avoid uncertainty in phasing, we used unphased genotypes to compute LD statistics, as proposed in Ragsdale and Gravel (2020).

Statistics were computed from intergenic loci, as described in Section 1.4, to avoid biases due to direct or indirect selection in protein-coding regions. To assess how strongly selection in coding regions affects LD and diversity statistics, we also computed statistics from all regions of the genome that pass the Thousand Genomes strict mask. Genome-wide statistics showed slight deviations from statistics inferred from intergenic regions alone (Figure S9), although the differences were smaller than the differences between our best-fit models and the data they were fit to. Nonetheless, we chose to use only intergenic data to avoid possible biases due to selection in and around protein-coding regions.

2.3 Estimating two-locus statistics with finite sample sizes

The approach from Ragsdale and Gravel (2020) provides unbiased estimates of the LD statistics considered here, which can be directly compared to expectations for those same statistics computed from the multi-population Hill-Robertson system. Estimated statistics for populations with smaller sample sizes have greater uncertainty, which is accounted for by computing variances and covariances via bootstrap over genomic regions, assuming that there is no correlation between distant genomic regions (i.e., no long-range LD).

Some statistics, such as $\mathbb{E}[D_i D_j]$, require at least two diploid samples from a population to compute. Since we used a single Neanderthal sample, such statistics for the Neanderthal population were not used in the fit. By contrast, there are statistics that only require a single sample in a given population to estimate. These include cross-population nucleotide diversity measures, as well as some LD statistics involving more than one population. For example, statistics of the form $D_{\text{human}}(1-2p_{\text{human}})(1-2q_{\text{neanderthal}})$ require a single Neanderthal sample and are informative of the Neanderthal demography. These statistics were included in the fit, but statistics requiring more than one Neanderthal sample to estimate were removed.

2.4 Computing conditional SFS

The conditional site frequency spectrum (or cSFS) is the distribution of allele frequencies restricted to loci that satisfy a given condition. Specifically, we consider the distribution of allele frequencies in present-day populations conditioned on the Vindija Neanderthal carrying the derived allele relative to the inferred ancestral allele. Ancestral alleles alleles were determined from a 6 primate alignment (1000 Genomes Project Consortium *et al.*, 2015). This cSFS is expected to be close to uniform under neutrality and a simple split model (with no subsequent migration) between the ancestors of humans and Neanderthals (Chen *et al.*, 2007). By contrast, a U-shaped distribution has been taken as evidence for introgression from an archaic hominin population that split from humans at least as far back in time as the human-Neanderthal split (Yang *et al.*, 2012; Durvasula and Sankararaman, 2020). Sites with no calls in the Vindija Neanderthal were excluded from this analysis.

Because we were concerned that cSFS analyses may be affected by incorrect inference of the ancestral allele (Hernandez *et al.*, 2007), we computed the cSFS for all mutations, and for transitions and transversions

separately (Figures S20–S23). Comparisons of these observed cSFS with model predictions are discussed in the model prediction section below.

3 Model specification and fitting

As is commonly done in demographic inference, all models we considered are initialized from an ancestral panmictic population at mutation-recombination-drift equilibrium. This allows for rapid initialization of the summary statistics to reasonable values. The size N_e of this ancestral population is a model-specific adjustable parameter that also serves as a reference size to define subsequent population size changes.

For the early history following this initialization, we tested model parameterizations that cover many of the proposed scenarios of population structure, size changes, and/or archaic hominin admixture. The simplest model, in terms of number of parameters, was a single-origin expansion of humans, with no structure in the stem and no archaic hominin admixture aside from the Neanderthal admixture in Eurasian populations following the out-of-Africa migration. This model allowed for a population size change in the stem of humans between the ancestral split of the human-Neanderthal lineages and the more recent split of branches leading to southern and western/eastern African populations. The recent expansion and the African multi-regional models (Figure 1A and D) have the same topology, so interpretation of the model depends on the specified or inferred divergence times. Thus, because model optimization explores a wide range of parameter values, we account for both scenarios with this parameterization.

To include population structure in early human history, we considered multiple parameterizations of models that allowed more than a single stem population. In general, stem populations were allowed to vary in their sizes, split times, and migration rates, with parameterizations flexible enough to encompass proposed scenarios of either archaic hominin admixture or population structure, both connected by gene flow or with isolation between stems.

In one parameterization of early structure, which we refer to as a "continuous migration" model, a secondary stem population (stem 2) split from the primary stem (stem 1) that leads to contemporary humans. Stem 1 contributed to present-day populations via a series of population splits similar to the single-origin model, while stem 2 contributed through continuous symmetric migrations with contemporaneous populations. The symmetrical migration rates could differ across population pairs and over different epochs. This continuous migration was allowed until stem 2 disappeared, which occurred as recently as 5ka. We tested models that both allowed or disallowed migration between stems, i.e., before stem 1 split into S/E/W African populations.

In another parameterization of early structure, a secondary stem population (stem 2) contributed ancestry to present-day populations via a series of instantaneous admixture events (i.e., pulse admixture or "merger" events) to lineages leading to sampled present-day African populations. Merger events were allowed to occur in one or more of the Nama, Mende, and Gumuz branches, as well as the branch of East and West Africans prior to their split. Those admixture events were allowed to occur at any time along those branches, and with any proportions, and stem 2 was allowed to split from the primary stem at any time before subsequent divergences (and either before or after the split of the Neanderthal branch). We tested models that allowed migration between the early stems, before subsequent splits and admixture events, as well as models that were restricted to isolation between stem branches. Depending on the specific parameters, such models encompass commonly-considered scenarios for admixture from a ghost archaic hominin population (e.g., if a long-isolated lineage more recently contributes a minority of ancestry to one or more populations), as well as relatively simple fragmentation-coalescence scenarios.

Based on the geographical locations of present-day populations, we tentatively labeled ancestral branches using a parsimony in migration, referring to South, East, and West African branches (S/E/W AFR, Figures 1, 2). We do not know the geographical location of these ancestral populations (nor even if they correspond to populations with a well-defined geographic range), and these labels should be considered as tentative. However, we found it useful to name branches in reference to where in Africa their descendants are found. Parameters were converted to physical units (years, effective population sizes, etc) assuming a human generation time of 29 years.

3.1 General strategy for building models and introducing complexity

With up to six sampled populations in final demographic models that we fit, there are many parameters to learn. Even in the simplest model involving all populations (such as the tree-like single-origin model), there are a few dozen parameters defining split times, migration rates, admixture timings and proportions, and population sizes and size changes. Thus, parameter space for a given model topology is large. In addition, as the number of populations increases, the number of possible topologies also increases, as there are more possibilities for the order of divergence and admixture events.

In order to narrow the set of possible models to plausible scenarios and to avoid overfitting, we took an approach that combined the incremental addition of complexity, starting with fewer populations before combining all populations, as well as fixing parameters that have been previously estimated or that fit consistently across all model scenarios. By initially considering sets of two or three populations, we were able to narrow down the relative orders of divergences between African and Eurasian populations. Assuming simple isolation-with-migration models, the Nama appeared to be the earliest diverging population of those we considered, with West African (Mende) and East African (Gumuz) populations diverging more recently, followed by the split of the Eurasian branch from the East African branch.

We performed an initial round of optimization including all six populations with a family of models including single- and multiple-stem scenarios as described in the previous section. We identified some parameters that reached consistent values across all models:

- Neanderthal/human split: 550ka
- Eurasian back-to-African migration: 12ka

When testing multiple variations of the more complex models, we kept these values fixed (see Table S2 for a complete list of all fixed parameters). This reduced the potential for overfitting the more complex models, while reducing the computational cost of optimization.

Our models also included recent events to account for known migrations, admixtures, and growths and declines in effective population sizes. Many of these parameters were fixed based on previous historical, genetic, or anthropological research, namely

- Western African vs. eastern African divergence: 60ka (Pagani *et al.*, 2015; Chevrier *et al.*, 2018; Niang *et al.*, 2020)
- Eastern Africa vs. British divergence (i.e., OOA): 50ka (Mellars, 2011; Fu *et al.*, 2014; Hublin *et al.*, 2020)
- Neanderthal introgression to Eurasian branch: 45ka (Reilly *et al.*, 2022)
- Mende population expansion: 5ka (Tennessen *et al.*, 2012)
- Gumuz population decline: 5ka (Gopalan *et al.*, 2022)
- East African pastoralist to South African admixture: 2ka (Henn *et al.*, 2008)
- European to South African admixture: 10 generations ago (Uren *et al.*, 2016)
- South African population decline following colonial admixture: 9 generations ago (van Eeden *et al.*, 2022)

The total number of parameters that were ultimately inferred were twenty-one to thirty-one, depending on the complexity of the model.

3.1.1 Evidence for the Out of Africa timing

Above (Section 3.1), we assume that the eastern Africa-Eurasian population divergence occurs at 50ka and Neanderthal admixture into this Eurasian ancestral population occurs at 45ka. The choice of these dates deserves additional discussion and consideration. Our focus is on the possible expansion Out of Africa (OOA) that led to a sustained, widespread population from whom present-day OOA populations are derived.

In this context, a number of possible models have been outlined (Groucutt *et al.*, 2015). Among these, Groucutt *et al.* enumerate variants on an early onset dispersal model. There is persuasive paleoanthropological evidence for a *Homo sapiens* presence in Arabia between 85–120ka, coincident with Marine Isotope Stage (MIS) 5. The crania identified at Skhul and Qafzeh in the Levant, as well a finger bone from southern Arabian Peninsula point to a dispersal OOA when conditions in North Africa facilitated a connection through the Sinai land bridge, and/or a possible connection via crossing the Red Sea. Ecological conditions would have been similar between North Africa and the Arabian Peninsula at this time, with greater abundance in game during the higher rainfall MIS 5. While Skhul and Qafzeh are taxonomically assigned to *Homo sapiens*, we note that they are morphologically quite different from contemporary populations (Pearson, 2008). Under some formulations of the early onset dispersal model, archaeologists posit a further expansion of humans into South Asia or as far as southeastern Asia (Groucutt *et al.*, 2015).

However, the replacement of *Homo sapiens* by Neanderthals in the Levant (e.g. Kebara 2) complicates the persistence of modern humans in the Arabian Peninsula after 85ka (Valladas *et al.*, 1987). The end of MIS 5 coincides with increasing aridity in the region which would have rendered much the Peninsula uninhabitable, except for a secluded southern refugia, decreasing the probability of migrations through the Sinai land bridge (Beyer *et al.*, 2021). This barrier to human migration OOA via the northern route likely remained until the Holocene (as modeled by precipitation amount). A southern route across the Red Sea becomes possible during MIS 4 (70ka) until 30ka (Beyer *et al.*, 2021). Even during MIS 4, there were few plausible routes out of the southern Arabian Peninsula, except along the Gulf of Oman or gaps in the Arabian Desert through the Zagros Mountains. Neanderthal remains dating to this period have been identified in the Zagros Mountains, adding a further potential barrier to expansion (Yousefi *et al.*, 2020; Heydari-Guran *et al.*, 2021).

Thus, either an incipient OOA population must have expanded beyond the Near East by 85ka and then given rise to today's Eurasian populations or a successful expansion would likely post-date 65ka. Morphological and archaeological evidence for *Homo sapiens* in the Near East reappears by 50ka in the Levant with the Initial Upper Paleolithic stone tool industry (Boaretto *et al.*, 2021) and a cranium from Manot Cave (Hershkovitz *et al.*, 2015). There is also strong consensus that a widespread human presence in Europe and Asia is dated to after 45ka. Archaeological (e.g., Fewlass *et al.*, 2020), morphological (e.g., Hublin *et al.*, 2020), and genetic (e.g., Prüfer *et al.*, 2021) data all indicate that about 45ka *Homo sapiens* had expanded into eastern Europe and within 5,000 years rapidly colonized the entire span of southern Europe. This period further coincides with the dates for the last Neanderthal presence in Europe at approximately 40ka (Devièse *et al.*, 2017; Hublin, 2017). Similarly in Asia, evidence of the Initial Upper Paleolithic (IUP) occurs 39-45ka as far east as Lake Baikal. Characteristics of the IUP support a coherent, cultural package which may have been transmitted by demic expansion (Kuhn and Zwyns, 2014; Zwyns, 2021). By 45ka, there is additionally an unequivocal *Homo sapiens* femur from southern Siberia which contains derived alleles shared with later Eurasian populations (Fu *et al.*, 2014). The Ust'-Ishim specimen contains evidence of admixture with Neanderthals, at a level similar to contemporary East Asian genomes. This supports estimates of Neanderthal admixture occurring earlier than 45ka, estimated to be by 47-65ka as inferred from Neanderthal tract lengths (Sankararaman *et al.*, 2012).

In summary, a sustained, rapid human expansion across Eurasia dates to just prior to 45ka and emerges from a Near Eastern population. The time of population divergence between eastern Africans and this Near Eastern group is difficult to pinpoint. An exit OOA and persistence prior to 65ka appears unlikely to have contributed to the ancestors of most Eurasians. By 50ka, a *Homo sapiens* population occupied the Levant, subsequently admixed and replaced Neanderthals, and is a likely source for the Eurasian populations. Hence, we fixed the eastern African/Eurasian divergence at 50ka, though arguably dates even earlier up to 65ka would be appropriate (see SI Section 7.2).

We tested our models for sensitivity to a subset of the fixed parameters, including those relevant to the OOA timing. When unconstrained, the inferred population split times between eastern African populations and Europeans occurred around 40ka, and the inferred split between eastern and western African populations occurred about 10ka earlier. The 95% CI for the inferred divergence times (which accounts for the non-independence of sites) does not contain the values of the fixed estimates. Under the high-likelihood models, the 95% confidence intervals for the eastern African-European divergence were roughly 38 ± 5ka, and for the eastern/western African divergence was 45 ± 10ka, with the uncertainties reflecting 2 standard deviations according to bootstrap of genomic regions.

We hypothesize that these discrepancies result from model misspecification related to the divergence between the ancestors of OOA and eastern African populations. As is typical, our models suppose a single split leading to the OOA event followed by constant, symmetric migration between eastern African and OOA populations until 12ka. We consider the British population (GBR) as a proxy for all Eurasians populations, in part to avoid adding many additional parameters associated with including 2 or 3 Eurasian populations. Further, our study and most others lack representative genomes from the Arabian Peninsula and Levant.

3.2 Likelihoods, uncertainty, and confidence intervals

`moments-LD` uses a composite multivariate Gaussian likelihood approach to simultaneously fit relative pairwise diversity and LD statistics over a range of recombination distances. This composite likelihood and Gaussian assumption are described in more detail and validated in Ragsdale and Gravel (2019), but we outline them here. To compute likelihoods, we need an estimate of the joint distribution of summary statistics. We estimate uncertainty due to the finite amount of genetic material used in inference using bootstrap over 500 segments along the genome with roughly equal lengths of retained sequences within each segment. First, for each distance bin, we use these bootstrap samples to obtain a variance-covariances matrix across all statistics. This variance-covariance matrix is used to obtain a model likelihood for each recombination distance bin and single-locus nucleotide diversity, as a multivariate Gaussian likelihood. The full model likelihood is taken as the product of likelihoods over each bin. In other words, we followed the approach of Ragsdale and Gravel (2019) in optimizing a composite likelihood where observations in different bins were taken to be independent. We computed 95% confidence intervals for each model using inferences from 200 block-bootstrapped datasets, which were fit using the same procedure as the original data. From the resulting parameters from these fits, we removed the bottom 2.5% and top 97.5% values and report the range of the remaining fit parameter values.

Tables S3–S7 give the best fit values for all parameters in the four models considered here, along with 95% confidence intervals from the refit bootstrapped datasets. Figures S10–S13 show the corresponding fits to many of the LD statistics used in the inference. The σ_{Dz} statistics are fit poorly by the single-origin model, while the models with multiple stems all provide reasonable fits to the data. The merger-with-stem-migration model performed the best of these four models, although the fits were nearly visually indistinguishable from the fits from the merger-with-stem-migration model.

A simulation-based validation of the confidence interval calculation is provided in Section 7.5.

3.3 Optimization using `moments`

For each model tested, we ran multiple rounds of likelihood optimization, alternating between the *optimize_log_fmin*, *optimize_log_powell*, and *optimize_lbfgsb* methods to explore parameter space and hone in on the best fit parameters. We imposed bounds on many of the parameters in the fits. This is done primarily to avoid optimisation exploring parts of the parameter space that are unrealistic (e.g., negative migration rates), where the model calculation can be costly (e.g., population splits billions of years ago), and where model assumptions can break down (e.g., very small populations). Population sizes had a lower bound of $N_e = 100$, migration rates were bounded between 0 and 10^{-3}, and admixture proportions between 0 and 1. Event times were only constrained to occur in the proper order (so that populations were required to have positive existence times, for example). Most best-fit parameters were away from these boundaries.

Initial guesses for parameters were chosen from demographically plausible starting points and then perturbed to explore space, using gradient descent (on the log of the parameters). The best fits from these initial rounds of optimization were then chosen as the starting points for optimization using the Powell and/or the L-BFGS-B methods (as implemented in the SciPy optimization package (Virtanen *et al.*, 2020)). This process was repeated with alternating optimization methods until the best fit parameter set converged consistently.

4 Gene genealogy reconstruction

We used `Relate` version 1.0.16 (Speidel *et al.*, 2019) to reconstruct genome-wide gene genealogies using a combined set of 1000 Genomes and African Diversity Reference Panel datasets, retaining all AFR-labeled

populations and GBR, CEU, PJL, and CHB from the 1000 Genomes panel and the Nama, Gumuz, Oromo, and Amhara from ADRP. We used all autosomes and applied the 1000 Genomes Phase 3 strict mask, we used an ancestral sequence determined by a 6-primate alignment (human_ancestor_GRCh37_e59), and we used the HapMap II combined recombination map, all in GRCh37 coordinates. We assumed a generation time of 29 years, a mutation rate of 1.25e-8, and followed the standard pipeline described in the `Relate` documentation.

From the reconstructed gene genealogies, we computed coalescence rates within and between populations using `Relate`'s function `RelateCoalescentRate`. This allows for an estimate of the instantaneous inverse coalescence rate (IICR) for samples drawn within each population (the inverse of which is often interpreted as the effective population size history), and the relative cross coalescence rates between pairs of populations (which are commonly used to estimate divergence times).

Following Speidel *et al.* (2019) we also identified "deep branches" within gene trees. Such a "deep branch" is a branch within a marginal tree (meaning, the tree at a given locus) that has its upper end (i.e., the node of its coalescence with another branch) extending to more than 1 million years in age, and we partitioned deep branches based on their lower node ages (where two or more branches coalesce into this single branch) into bins between 0 and 1Ma. Such branches can be categorized by their association with Neanderthals by comparing mutations that fall upon such a branch to the allele found in a Neanderthal genome sequence. For this, we used the published high-coverage Vindija Neanderthal (Prüfer *et al.*, 2017). Again following the analysis in Speidel *et al.* (2019), if one or more mutations on a deep branch (carrying at least two derived mutations) are shared with the Neanderthal sequence, the branch is inferred to have passed through the Neanderthal lineage.

A deep branch is assigned to a contemporary population if at least one sample from that population has ancestry that passes through that branch. Speidel *et al.* (2019) show that deep branches with lower ends more recent than the Neanderthal introgression event are enriched for Neanderthal-matching alleles in Eurasian populations, while a large majority of deep branches in 1000 Genomes West African populations do not match either the Neanderthal or Denisovan samples. This observation was taken as further evidence for deep population structure or archaic hominin admixture in West African populations from an unidentified hominin unrelated to the Neanderthal/Denisovan complex.

5 Predictions from inferred demographic models

5.1 Divergence between coexisting populations over time

We considered a few different measures of differentiation between populations in four inferred models, starting with F_{ST}. For each of the four inferred demographic models presented here, we computed predicted F_{ST} for pairs of contemporaneous populations over time.

F_{ST} represents the proportion of genetic variance in a multi-population sample that can be attributed to drift between populations in that sample (e.g., Bhatia *et al.* (2013)). F_{ST} depends on population divergence times, gene flow, and population sizes. For present-day sampled populations, predicted F_{ST} can be compared to data (Fig. S14), while past estimates represent model predictions about the differentiation between past populations. We computed F_{ST} as a ratio of expected (or average) variances, using observed pairwise diversity within and between populations as in Peter (2016):

$$F_{ST}(i,j) = \frac{\mathbb{E}[2H_{i,j} - H_i - H_j]}{\mathbb{E}[2H_{i,j} + H_i + H_j]}, \qquad \text{(S2)}$$

where H_{ij} is the average number of differences between one haploid copy of the genome from population i and one from population j, and H_i and H_j are the average number of differences for two haploid copies from the same population.

In all inferred models, F_{ST} between Neanderthals and the ancestors of contemporary humans were much larger than between any pair of contemporary human populations or their ancestors (stems). F_{ST} between human populations over the last $100ka$ remained relatively low, at ~ 0.1 or less. In the continuous-migration model, the two stem populations also were predicted to have low F_{ST} within this range, as migration rates were inferred to be high enough to maintain genetic similarity. In both the merger-without-stem-migration and the merger-with-stem-migration, one of the stem populations was inferred to have a short,

severe bottleneck, which led to increased F_{ST} between stem populations during that time. In the merger-with-stem-migration, F_{ST} reached a maximum value of 0.3 between stems, intermediate between human-Neanderthal divergence (0.45) and the maximum F_{ST} observed between contemporary human populations ($\lessapprox$ 0.1.)

Whereas the high F_{ST} value between Neanderthal and humans reflects both elevated genetic differences between the genomes of humans and Neanderthals and reduced diversity within the Neanderthal population (Fig. S14), the increased F_{ST} between the stems is primarily due to an inferred bottleneck reducing stem diversity, while genetic differentiation between individuals from the two stems is within the range of genetic differentiation between (and even within) human populations. In other words, our best fit model describes two stems with one stem showing reduced diversity, but individuals across stems showing similar differentiation to that between two contemporary human population.

5.2 Shared differentiation between pairs of contemporary and pairs of ancient populations

To evaluate the degree to which divergence among contemporary human populations is inherited from divergence between early stem population, we computed f_4 statistics of the form

$$f_4(\text{stem}_1, \text{stem}_2; \text{present}_1, \text{present}_2) = \mathbb{E}[(p_{\text{stem}_1} - p_{\text{stem}_2})(p_{\text{present}_1} - p_{\text{present}_2})] \tag{S3}$$

This statistic measures co-linearity of genetic drift between contemporary populations $v = (p_{\text{present}_1} - p_{\text{present}_2})$ and stem populations $w = (p_{\text{stem}_1} - p_{\text{stem}_2})$ (see Figures S16–S19 and Lipson (2020) for an overview of f_4 statistics).

To contrast these statistics to divergence across contemporary populations,

$$f_2(\text{present}_1, \text{present}_2) = \mathbb{E}[(p_{\text{present}_1} - p_{\text{present}_2})^2], \tag{S4}$$

we interpret the f-statistics as scalar products between vectors v and w, i.e., $f_2(\text{present}_1, \text{present}_2) = v \cdot v$ and $f_4(\text{stem}_1, \text{stem}_2; \text{present}_1, \text{present}_2) = v \cdot w$. We can then write $v = v_\perp + v_\parallel$ as the sum of a component $v_\perp$ perpendicular and a component $v_\parallel$ parallel to $w = (p_{\text{stem}_1} - p_{\text{stem}_2})$. We can then decompose $f_2(\text{present}_1, \text{present}_2) = v_\perp^2 + v_\parallel^2$, with

$$v_\parallel^2 = \frac{(v \cdot w)^2}{w \cdot w} = \frac{f_4^2(\text{stem}_1, \text{stem}_2; \text{present}_1, \text{present}_2)}{f_2(\text{stem}_1, \text{stem}_2)} \tag{S5}$$

Finally, the proportion of present-day structure $v^2 = f_2(\text{present}_1, \text{present}_2)$ that is accounted for by the component parallel to ancient population structure is

$$\alpha^2 = \frac{v_\parallel^2}{v^2} = \frac{f_4^2(\text{stem}_1, \text{stem}_2; \text{present}_1, \text{present}_2)}{f_2(\text{stem}_1, \text{stem}_2) f_2(\text{present}_1, \text{present}_2)}. \tag{S6}$$

α^2 can be interpreted as the proportion of the f_2 divergence across contemporary populations that is aligned with the differences in allele frequencies across ancient populations. However, drift occurring after the time of sampling of ancient populations is not correlated to drift occurring before that time. For this reason, we also interpret α^2 as the proportion of the f_2 divergence across contemporary populations that is *explained by* the differences in allele frequencies across ancient populations. We report this predicted statistic for each of the four inferred demographic models in Figures S16–S19. In our best fit models (continuous migration and merger with stem migration), differentiation between Stem 1 and Stem 2 (before the split of Stem 1 into Stem 1E and Stem 1S in the merger model) contributes a maximum of 4% to differentiation between the Nama and Gumuz, and closer to 1% to differentiation between the Nama and Mende or the Mende and Gumuz.

5.3 Back-to-Africa Gene flow and Neanderthal ancestry in Africa

Back-to-Africa migrations likely introduced Neanderthal ancestry into Africa (Chen *et al.*, 2020). This Neanderthal ancestry would cause reticulation in the ancestry of the African populations, and is therefore

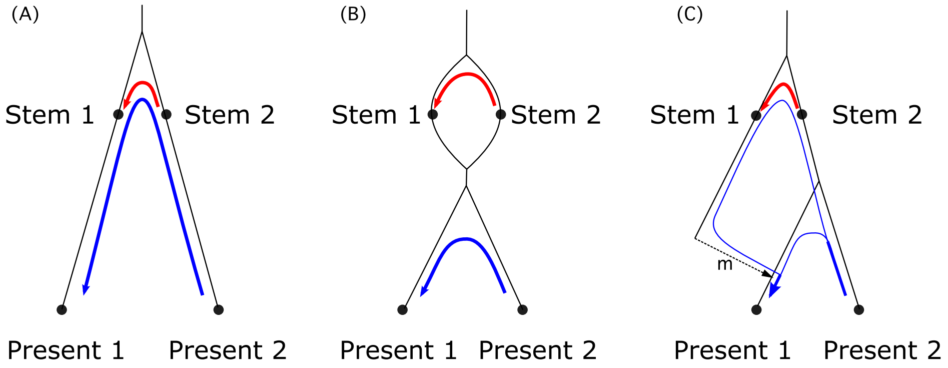

Figure S1: **Present-day structure attributable to ancient population structure.** The statistic $f_4(\mathrm{stem}_1, \mathrm{stem}_2; \mathrm{present}_1, \mathrm{present}_2)$ captures how much of present-day population structure can be attributed to population structure among the stems. The blue line represents genetic drift along ancestral branches between contemporary populations, while the red line represents drift between stem populations. f_4 measures the overlap between the blue and red line, which corresponds to shared genetic drift between the two pairs. In scenario A, contemporary populations are descended from distinct stems, and $f_4(\mathrm{stem}_1, \mathrm{stem}_2; \mathrm{present}_1, \mathrm{present}_2)$ equals the drift between stems, $f_2(\mathrm{stem}_1, \mathrm{stem}_2)$. In scenario B, despite deep population structure, there is no shared drift and $f_4 = 0$. That is, present-day population structure is unrelated to structure among stems. In more realistic scenario C, where $\mathrm{present}_1$ receives a proportion m of its ancestry from the dotted arrow, the overlap is only found along the corresponding path, and f_4 depends on both ancestry proportion m and drift between stems, $f_4(\mathrm{stem}_1, \mathrm{stem}_2; \mathrm{present}_1, \mathrm{present}_2) = m f_2(\mathrm{stem}_1, \mathrm{stem}_2)$

relevant to the modeling presented here. The models we inferred account for both Neanderthal admixture in Eurasia and Back-to-Africa migrations, such that they predict the amount of Neanderthal ancestry that we would expect to find in African individuals. We find that African populations without high proportions of recent Eurasian ancestry are predicted to carry $0.1 - 0.2\%$ Neanderthal ancestry (Table S8), which is remarkably consistent with the results of Chen *et al.* (2020). Since the IBD statistics from Chen *et al.* (2020) are not used in the present analysis, this serves as an independent validation of the present models. More importantly, since the Neanderthal admixture in Africa is properly accounted for in the model, we do not expect the presence of such ancestry to bias our estimate of reticulation in Africa.

5.4 Relating model predictions to morphological variation

Here we show how the predictions of our model can be related to morphological variation in the fossil record. Let Δ^2 be the expected squared difference in a morphological measurement between fossil 1 and fossil 2,

$$\Delta^2 = \mathbb{E}\left[\left(\bar{X}_1 - \bar{X}_2\right)^2\right] + \mathbb{E}\left[V_1\right] + \mathbb{E}\left[V_2\right], \tag{S7}$$

where $\bar{X}_1$ and V_1 and $\bar{X}_2$ and V_2 are measurement means and variances for the populations from which fossil 1 and fossil 2 are sampled.

If we assume that the trait being measured is evolving neutrally, which seems reasonable as an approximation based on studies of morphological (cranial) variation in humans and Neanderthals (Relethford, 1994; Weaver *et al.*, 2008; von Cramon-Taubadel, 2009), then

$$\mathbb{E}\left[\left(\bar{X}_1 - \bar{X}_2\right)^2\right] = 4\sigma_m^2 \tau_B - 2\sigma_m^2 \tau_{W_1} - 2\sigma_m^2 \tau_{W_2}, \tag{S8}$$

$$\mathbb{E}\left[V_1\right] = \frac{1}{h^2}\sigma_m^2 \tau_{W_1}, \tag{S9}$$

and

$$\mathbb{E}\left[V_2\right] = \frac{1}{h^2}\sigma_m^2 \tau_{W_2}, \tag{S10}$$

where σ_m^2 is the additive genetic variance introduced by mutation (per zygote per generation), h^2 is the (narrow sense) heritability, τ_{W_1} and τ_{W_2} are the average coalescence time of pairs of alleles from the same population, and τ_B is the average coalescence time of pairs of alleles from different populations (Whitlock, 1999; Weaver, 2016). Substituting and simplifying gives

$$\Delta^2 = \sigma_m^2 \left(4\tau_B + \frac{1-2h^2}{h^2}\left(\tau_{W_1} + \tau_{W_2}\right)\right), \tag{S11}$$

which relates Δ^2 to between and within population coalescence times.

As a further approximation, if we assume that the heritability of the measurement is close to one half, then

$$\Delta^2 \approx 4\sigma_m^2 \tau_B, \tag{S12}$$

which shows that under these assumptions Δ^2 is proportional to τ_B.

Nucleotide diversity, π, for pairs of individuals sampled from different populations is also proportional to τ_B. Therefore, ratios of Δ^2 can be predicted from ratios of π for the same comparisons. For example, a ratio for which π for Stem 1 and Stem 2 is the numerator and π for a pair of contemporary human populations is the denominator can be used to predict Δ^2 for two fossils sampled from each of the two stems based on Δ^2 for contemporary humans. In this way, the predictions of our model can be related to morphological variation in the fossil record.

6 Model validation strategy

We took two approaches to validate demographic model inferences. First, we explored the effects of using alternative populations, sample sets, and recombination maps, and the effect of relaxing fixed parameters when fitting the demographic models to diversity and LD statistics. We then use simulations to test our inferred model predictions against other informative summary statistics. Results are shown in the Supplementary Results (Section 7).

6.1 Validations using alternative data sets and model specification

6.1.1 Alternate Thousand Genomes population sets

In our primary model fits, we used the Mende in Sierra Leone (MSL) and the British from England and Scotland (GBR). We tested the same models against a different West African population (the Yoruba from Ibidan, Nigeria, YRI), and two different European populations (western Europeans from Utah, CEU, and Toscani in Italy, TSI). The CEU are closely related to GBR and are thus likely to be an equivalently close proxy for European ancestry in African individuals with admixed European ancestry. The TSI are likely more distantly related to the source of this European ancestry. We chose not to replace the GBR by non-European Eurasian populations, as these would be poorer proxies for early Holocene and recent colonial migration from the Near East and Europe back to Africa, and it is unclear how such a model should be interpreted. We therefore refit each of our best-fit demographic models using the following alternate population sets: (Nama, YRI, Gumuz, Oromo/Amhara, and CEU), and (Nama, MSL, Gumuz, Oromo/Amhara, and TSI).

6.1.2 Variable thresholds of Nama-associated ancestry

The Nama have recent European ancestry, and Nama individuals in our dataset show varying levels of inferred ancestry relating to European populations (Figure 2D, in the main text). Each of our inferred models allows for gene flow from Eurasia to Africa after the out-of-Africa migration event, through early Holocene back-to-Africa migration in East Africa, recent colonial admixture in South Africa, and ongoing continuous migration between branches leading to East African and European populations. While our demographic models directly account for European ancestry in Nama individuals, inferred parameters relating to early divergences and population structure may be impacted by recent admixture.

To assess the impact of recent European admixture in Nama individuals on inferred early divergence times and population structure, we subset the set of Nama individuals that we included based on inferred

European-matching ancestry proportions. We used inferred ancestry proportions from ADMIXTURE with the number of clusters $K = 4$ (Section 1.5) and kept individuals with either at least a) 90% or b) 99% proportion of inferred ancestry that clusters within the Nama group (colored green in Figure 2D). This left a) 18 Nama individuals with at least 90% inferred South African ancestry, and b) 14 Nama individuals with at least 99%. For each subset, we recomputed diversity and heterozygosity statistics and fit each of our demographic models to these statistics.

6.1.3 Varying fixed divergence times

In order to reduce parameter space to avoid overfitting, we fixed parameters that have previously been inferred, including the timings of the early back-to-Africa migration contributing to the ancestry of East African agricultural populations (12ka), the divergence of West and East Africans (60ka), and the out-of-Africa migration event (50ka). Due to variability in inference and dating methods, there is some uncertainty surrounding these dates. We explored how variable timings of these events effect our inferred models by 1) increasing the fixed times of the E-W African split (to 100ka) and the out-of-Africa event (to 60ka), and 2) allowing those two parameters to be jointly fit in model optimization.

6.1.4 Recombination map inferred from Nama

Throughout, we used inferred recombination maps based on the YRI OMNI array data (1000 Genomes Project Consortium *et al.*, 2015). Because recombination rates can vary between populations, we tested the robustness of our demographic inference to the recombination map used to compute LD. We recomputed diversity and LD statistics using recombination rates from the Nama recombination map (van Eeden *et al.*, 2022), and refit our best-fit demographic models to these statistics.

6.2 Validations using simulations from inferred demographic models

Here we focus on four parameterizations of early human history: (1) a "single origin" with recent expansion, (2) primary and second stem populations with "continuous migration", (3) two stem populations that do not exchange migrants before merging to form regional African populations, referred to as a "merger without stem migration", and (4) two stem populations that are allowed to exchange migrants before merging to form regional African populations, referred to as a "merger with stem migration". Given the best fit parameters for each model (Tables S3–S7), we used `msprime` version 1.0.4 (Kelleher *et al.*, 2016; Baumdicker *et al.*, 2022) to simulate sampled individual genomes to match the data used in inference (see simulation details below). We used these simulations to compare our model predictions to predictions based on the conditional SFS (cSFS) and on relate curves (see below).

These comparisons serve as independent validations of our inferred demographic models by assessing how well those models fit aspects of the data that have been used in other studies as evidence of population structure or archaic hominin admixture from an unidentified hominin species within Africa.

6.2.1 Simulation details

The four inferred demographic models (single origin, continuous migration, merger without stem migration, and merger with stem migration) were converted to `demes` format (Gower *et al.*, 2022) and used as the input demography for `msprime` simulations. We sampled the same number of individuals from each population as used in the inference (Nama: 44, Mende: 85, Gumuz: 23, combined Oromo/Amhara: 46, British: 91, and Vindija Neanderthal: 1). We assumed a generation time of 29 years, a per-base mutation rate of 1.25×10^{-8}, a per-base recombination rate of 1×10^{-8} (corresponding to 1cM/Mb), and we simulated 100 replicates with sequence lengths of 50Mb per replicate. We computed the conditional site frequency spectrum (cSFS) conditioned on observing the derived variant in the Neanderthal sample, and reconstructed gene genealogies using `Relate` to obtain coalescence rate trajectories and distributions of deep branches passing through the Neanderthal branch. Each analysis followed the same procedure as described above in the analysis of the real data.

6.2.2 cSFS prediction under inferred models

We compared the observed cSFS to model predictions for the Nama, Mende, and Gumuz populations. The cSFS from data is sensitive to ancestral allele misidentification, but our simulations assume perfect knowledge of the ancestral allele. To investigate the role of misidentification, we compared predicted and observed cSFS for transitions and transversions separately as well as jointly.

For each comparison, we fit a misidentification parameter p_{misid} as well as a scaled mutation rate θ. The misidentification parameter is the fraction p_{misid} of loci that have the ancestral state misidentified. Accounting for p_{misid} mixes the predicted joint population SFS with a small amount of the ancestral-allele-reversed SFS (i.e. for a two-population joint SFS, $SFS_{misid}(i,j) = (1-p_{misid}) * SFS(i,j) + p_{misid} * SFS(n-i, m-j)$, where n and m are the sample sizes from the two populations). The cSFS for the first population (conditioning on the second) is found by projecting the sample size to $(n, 1)$ and taking $cSFS = SFS(:, 1)$. θ and p_{misid} were chosen to maximize a likelihood assuming that each observed entry in the cSFS is drawn from a Poisson distribution (Sawyer and Hartl, 1992).

7 Model validation results

7.1 Model fits using alternative datasets

All supplementary inference results discussed in this section are provided in Demes format (Gower *et al.*, 2022) as a supplemental zipped archive. For each scenario, we refit the continuous migration and multiple merger models, with stem migration.

7.1.1 Replacing Thousand Genomes populations

We replaced the Mende (MSL) and British (GBR) population sets with Yoruba (YRI) and western and northern Europeans (CEU), respectively, and refit our inferred demographic models to diversity and LD statistics computed from those populations (along with Nama, Oromo/Amhara, and Gumuz). Because the MSL and YRI share recent West African ancestry, and the GBR and CEU share recent European ancestry, we expected most parameters in inferred models using these two datasets to be largely similar. Indeed, all parameters inferred using the YRI and CEU data were well within one standard error of the same parameters inferred using the MSL and GBR data, across all tested models. The one exception to this was the recent size of the YRI branch, inferred to be somewhat smaller than the final size of the analogous MSL branch ($\approx 20,000$ vs. $\approx 30,000$, resp.). Other population sizes, migration rates, and divergence times were highly consistent.

Because CEU and GBR are genetically closely related, we also tested the consistency of inferred parameters when replacing GBR with Toscani from Italy, a more distantly related European population set than GBR. Most parameters in each of the tested models were consistent with parameters inferred using GBR. The only large difference was found in fitting the continuous migration model, where the Nama divergence was $\approx$ 200ka rather than 135ka. Our original model using the dataset with GBR underestimated both σ_{Dz} and σ_{π_2} compared to data (Figure S11), and as a result provided a poorer fit to the data than the multiple merger model. When fitting this same model to the TSI, the model provided a better fit to the σ_{Dz} statistics but a worse fit to the σ_{π_2} statistics (and still provided a significantly worse fit to the data compared to the multiple merger model). Migration rates during this early period of the model also shifted to larger values between the stem populations, demonstrating that divergence times and migration rates are confounded parameters in this model. These differences in inferred parameters are likely driven by a combination of factors: the continuous migration model mis-specifies historical dynamics in the range of 100-200ka; the TSI are an inappropriate proxy for recent European admixture in East and South Africa; and the TSI may also carry recent North African ancestry, which is not captured in our model. By contrast, the multiple merger model (which provides a better fit to the data) was inferred to have consistent divergence and merger times between the models fit using GBR and TSI, with ancestral populations "reshuffling" between 100 and 120ka.

In summary, across all validations to alternative datasets described in this section, model fits were largely robust and matched those shown in the main text (Figure 3)—the single outlier in these validations was the estimated divergence time for the Nama in the continuous migration model fit to the dataset that included

TSI instead of GBR. Thus, while specific aspects of the inferred demographic inference can be sensitive to the choices of populations to include and the choices of model specification, the results presented in the main text are broadly robust to choices of model, dataset, and analysis choices. Our conclusions in the main text took into account both this overall robustness and the important sources of uncertainty.

7.1.2 Fit to LD statistics from Nama recombination map

The LD statistics computed using the OMNI YRI recombination map and the Nama-specific recombination map were very similar, with only a handful of statistics showing slight visible differences (Figure S8). We therefore expected inferred demographic models to be highly consistent when fit to the two sets of statistics. Across each of our models, inferred parameters from fits using data from the Nama-specific map were unchanged, with best-fit values all within one estimated standard error from the inferred parameters from the OMNI YRI map.

Among human populations, the Nama are relatively highly diverged from the sampled populations used in estimating the OMNI recombination maps (such as the YRI), and the two recombination maps were inferred using different computational methods from different types of sequencing data (arrays vs. whole genomes). Therefore, differences between these two maps are likely near the upper bound for differences in recombination rates between present-day human populations (van Eeden *et al.*, 2022). Because our fits were highly consistent when using the two recombination maps, we conclude that inferences using the diversity and LD statistics here are robust to variation in recombination rates between present-day populations as well as to any differences in inferred recombination rates due to true recombination rate differences or typical statistical error in recombination rate estimation.

7.1.3 Varying Nama ancestry-proportion thresholds

In the demographic inference results presented in the main text, we did not impose a minimum threshold on inferred Nama ancestry (as estimated using ADMIXTURE). Because our proposed models allow for post-divergence gene flow as well as recent admixture events, we can jointly learn deeper history and recent admixture dynamics. This is in contrast to methods that do not account for ongoing migration or recent admixture events, for which un-admixed genomes (if they even exist) are needed for unbiased estimates of earlier history. Although the methods used in this paper (`moments-LD`) are able to infer early and recent history jointly, we tested the robustness of the inferred early history to varying ancestry proportions in the Nama population. To do this, we used ADMIXTURE to cluster inferred ancestry components, noting that ADMIXTURE-inferred ancestries are coarse and can be misleading about true underlying admixture proportions. Nonetheless, we included Nama individuals that exceeded either 90% or 99% ancestry primarily shared among Nama individuals.

For both thresholds, early history was robust in all tested models. Divergence times and migration rates did not vary significantly from the original fits that included all unrelated Nama individuals. The primary differences between these fits and the original fits that did not impose an ancestry threshold were the inferred admixture proportions of East African agriculturalists (2ka) and Europeans (10 generations ago) in the Nama population. The East African admixture proportion was reduced from $\approx 25\%$ to $20-22\%$, while the European admixture proportion was reduced from $\approx 15\%$ to either $5-6\%$ (with a 90% ancestry cutoff) or $3-5\%$ (with a 99% ancestry cutoff). No other parameters were strongly affected by subsetting the Nama individuals by ADMIXTURE-inferred ancestry proportions. Because only the inferred proportions of recent admixture in our model fits changed, we conclude that our inferences of early history are robust to recent admixture, as long as that recent admixture is accounted for in the demographic models.

7.2 Relaxed inference of divergence times

In the primary results presented in the main text (Tables S3–S7), we fixed a number of parameters that have been previously inferred or are well constrained by historical records (Section 3.1), including the Eurasian out-of-Africa expansion at 50ka and the divergence of western and eastern African groups at 60ka. To evaluate the effects of these choices on the inference of other parameters in the model, we first fixed those modeled divergence times to 60ka (for the out-of-Africa expansion) and 100ka (for the E/W African divergence). In the next section, we allow those parameters to be fit along with all other model parameters.

By fixing those divergence times to older dates, the inferred models preferred older dates for the other free divergence time parameters. These dates were stretched by a similar proportion that our fixed parameters were shifted, so that the Nama divergence time was inferred to be $\approx$ 150ka, rather than $\approx$ 130ka. Notably, older divergence times resulted in models with worse likelihoods than our primary presented results. Overall, these inferred models have a similar interpretation to the primary inference results, and the deep population structure was unaffected by these choices.

When we allowed the out-of-Africa expansion and eastern/western African divergence times to be jointly fit in model optimization, we found that the best-fit inferred E/W divergence time and the OOA expansion were more recent ($45-55$ka and $35-40$ka, resp.). The remainder of the parameters were largely unaffected by these shifts to more recent divergence times, and our interpretation of the inferred early history remains unchanged. The 95% confidence intervals around these parameters were relatively narrow ($\approx$ 5ka for the OOA split, $\approx$ 10ka for the East/West African split), although this uncertainty does not capture uncertainty due to model mis-specification (as discussed in Section 3.1).

In summary, fixing these parameters (i.e., East Africa-Eurasian split at 50ka, Neanderthal admixture at 45ka, see Section 3.1) resulted in an estimated value for the split between the ancestors of East and West African populations at around 60ka. This timing agrees with previous LD-based estimates of West African-Eurasian divergence times, of $\sim$ 61ka ($50-64$ka, 95% confidence interval) (Ragsdale and Gravel, 2019), which used a different set populations than those in this study. A date of 60ka is also in close agreement with inferred divergence times of $\sim$ 65ka from both SFS-based demographic inference, using synonymous variation from a subset of populations used in this study (Mende, British, and Han Chinese), as well as that of LD-based inference using these same groups, of 53ka ($43-63$ka, 95% confidence interval). Taken altogether, we decided to fix this value (60ka) in most subsequent analyses.

7.3 Alternative statistics for four models

7.3.1 Conditional SFS

The single-origin model fit the cSFS relatively poorly across the three populations compared to the models that allow structure between stem populations (Fig. S20). The model that best fit the cSFS was the merger-with-stem-migration model, which also provided the best fit to pairwise diversity and LD statistics in the initial optimization. For the Mende and Gumuz, the merger-with-stem-migration model fits the data very well, while it underestimates rare variants in the Nama cSFS (Fig. S23). Models learned from the two-locus statistics provided a better qualitative description of the cSFS than models previously fit directly to the cSFS (see, e.g., figure 1 in Durvasula and Sankararaman (2020)). We found that the inferred ancestral misidentification rates were concordant across populations. The inferred rates of ancestral misidentification were about twice as high for transitions relative to transversions as expected, qualitatively, from the higher transition mutation rate (Hernandez *et al.*, 2007).

7.3.2 Relate curves from inferred models

We used `Relate` (Speidel *et al.*, 2019) to reconstruct gene genealogies from the simulations under our four inferred demographic models. From each of these, following the same approach as for the analysis of the actual data, we computed coalescence rates within and between sampled populations. From the coalescence rates, we estimated the inverse instantaneous coalescence rate (IICR, often interpreted as an estimator of N_e) for each population, as well as relative cross coalescence rates between pairs of populations.

The IICR is only a reliable estimator of N_e in the absence of population structure. However, since IIRC are readily estimated and visually interesting, it is commonly attempted even when the assumption is not met. Even if the assumption is not met, the inferred IIRC can be interpreted as a summary statistic for which model predictions can be compared to data. It can therefore be informative about aspects of the data that our models fail to predict. We also note that the coalescence rates inferred from data used a larger set of populations from the combined 1000 Genomes/ADRP dataset, while our model simulations only included a subset of 5 populations. This may lead to differences in resolution of the inferences.

The data shows the characteristic "hump" of increased IICR (N_e) 100-300k for all populations, followed by a decrease in size among all populations that is sharpest in the Eurasian populations and also pronounced in the East African populations that have high proportions of Eurasian ancestry (Amhara and Oromo)

(Fig. S24). The model-inferred N_e curves also show this general trend for the more recent reduction in N_e (Fig. S26). In the the more distant past, N_e fluctuates in a manner that is broadly similar across models with observed "humps" of increased N_e. Despite the large differences in parameterizations and interpretations of the four inferred demographic models, each of them show reconstructed N_e trajectories that are qualitatively similar, highlighting the difficulty in learning complex demography in the deep past from reconstructed coalescence rate trajectories.

We also compared the relative cross-coalescence rates (RCCR) between pairs of populations in the four inferred demographic models (Figures S29–S32) to those in the data (Fig. S28). In the recent past (<10ka), each of the models show increased RCCR compared to the data. However, in the medium to more distant past, the RCCR provide a good match to the data in each of the four models, including both their timing and ordering of increased cross coalescences. The failure to match both the IIRC and RCCR in the recent past in all models suggests that additional parameters would be needed to account for recent increases in population sizes. As is the case with other methods that reconstruct size history trajectories from inferred coalescence rates (e.g. PSMC, MSMC), `Relate` has difficulty inferring coalescences that occur in the recent past.

7.3.3 Distribution of deep branch affinities to Neanderthal sequence

Following Speidel *et al.* (2019), we also characterized Neanderthal-matching deep branches (see Section 4) in our simulated data under the four inferred demographic models. Comparing deep-branch distributions from the data (Fig. S34) to the models (Fig. S35), we find consistent trends between each of the four models and the data in the ordering of proportions of lineages from each population that are labeled as Neanderthal-matching deep branches. This proportion is slightly over-estimated in each of the African populations compared to the data, but is closest in the merger-with-stem-migration model (which also fit the LD statistics the best of the four highlighted models). Given the qualitative discrepancies between the data IICR and the model IICR, the differences in Neanderthal branch affinities is unsurprising.

7.4 Re-inference of IM models from simulated data

Recent studies have focused on characterizing the timing of the earliest divergences among human populations, and such analyses typically fit simple isolation-with-migration (IM) or "clean split" models to observed allele frequencies or inferred coalescence times among and between populations (see Weaver *et al.* (2008); Bergström *et al.* (2021) for recent reviews). Simple IM models typically fit a constant N_e within the two diverged population, the split time, and a symmetric continuous migration rate (which could be fixed to zero, equivalent to the "clean split" model). We explored the effect of fitting such simplified models of history to data from more complex models by simulating the site frequency spectrum (SFS) under each of our four models for pairs of populations and reinferring an IM and clean split model for each pair. The simulations sampled 10 diploid individuals from each population, and we used `msprime` (Baumdicker *et al.*, 2022) to simulated 500 Mb of total sequence length (the sum over 500 replicates each 1 Mb in length) with constant recombination rate $r = 10^{-8}$ and mutation rate $u = 2 \times 10^{-8}$.

As expected, the clean split models consistently provide divergence times T that are more recent than the IM models. Because T and subsequent migration can be inversely confounded in SFS inference, fixing migration rates to zero results in more recent inferred T (Fig. S36). Even though each of our four models inferred divergences of all human populations ∼120ka, ancient structure and size changes in the simulations biases re-inferences and gives inflated estimates of T. Such an effect has been shown to occur when the ancestral population size increases prior to the population split (Momigliano *et al.*, 2021), which is the case in our inferred single-origin model. Re-inferred T between the Nama and other populations were >200ka under the IM model, and ∼50–130ka under the clean split model.

To understand the relationship between model specification and demographic inference, consider the five toy models shown on Figure S2: A) clean split 100ka, B) clean split 200ka, C) population growth at 200ka followed by split 100ka, D) split 200ka followed by migration until 100ka, and E) split 200ka followed by merger 105ka and a new split 100ka.

Models C), D), and E) show qualitatively similar patterns of coalescence rate. Given the same data, an IM model (such as D) will infer a potentially much deeper population divergence than growth model C and

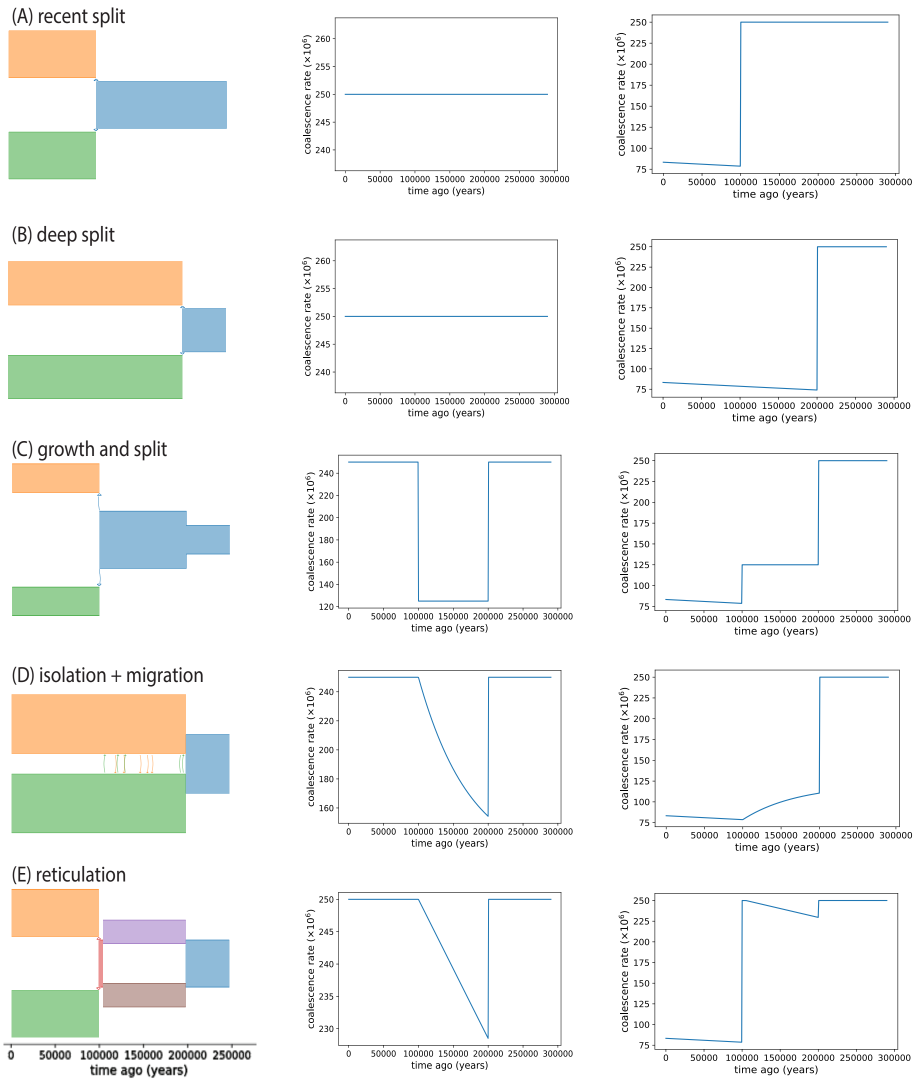

Figure S2: **The impact of model specification on coalescence rate and inference.** We show five simplified demographic models that differ in demographic events, occurring between 200ka and 100ka, that mark the transition between a single ancestral population and two daughter populations. A: A clean split 100ka, B: A clean split 200ka, C: population growth at 200ka followed by split 100ka, D: split 200ka followed by migration until 100ka, and E: split 200ka followed by merger 105ka and a new split 100ka. The left column shows model representation, the middle column shows instantaneous coalescence rates for pairs of lineages from the same daughter population, the right column shows instantaneous coalescence rates for pairs of lineages coming from distinct populations.

reticulation model E.

7.5 Evaluation of estimated confidence intervals

We estimated 95% confidence intervals (CIs) as reported in Tables S3–S7 by refitting each model to block-bootstrap replicated datasets. While our LD-based inference approach is expected to be unbiased (Ragsdale and Gravel, 2019, 2020), we tested whether our bootstrap-estimated CIs contain the true parameters using simulated data from our two highlighted models (the continuous migration model and the merger with stem migration model).

To do this, we simulated 500 1Mb regions using the same sample sizes as our analyzed data, with per-base mutation and recombination rates $u = r = 10^{-8}$, using `msprime` (Baumdicker *et al.*, 2022). From these simulated regions, we replicated our bootstrapping procedure by resampling (with replacement) 500 regions. We repeated this 500 times (to obtain 500 bootstrap replicate datasets). We fit the full (non-bootstrapped) simulated dataset and each of the 500 bootstrap replicates, and compared the inferred parameter values to the true simulated values (Figures S37 and S38).

The distribution of refit values for vast majority of parameters overlapped with the true simulated value. This included every parameter from the continuous migration model, and all of the parameters in the merger with stem migration model except for the size, migration rate, and split time of Stem 1 (which experienced a severe bottleneck in the deep past). Because the true simulated parameter for the bottleneck size in Stem 1 was at the lower bound ($N = 100$), this likely is due to the bottleneck re-inferences failing to fully converge. Overall, bootstrap-estimated CIs typically contain the true parameter, although CIs for parameters that lie at an optimization bound should be treated with caution.

Finally, many features of recent human history were consistently parameterized across each of our tested models (such as recent migration rates and population sizes). We evaluated the consistency of these recent parameters across our different models (Figure S39). Since all models use the same set of bootstrapped simulations, systematic differences between models reflect the effect of model specification rather than statistical uncertainty. The effects of statistical uncertainty and of model specifications need not be of the same order, but we find it useful to compare the two sources of uncertainties. Because model specification differences are mostly in the distant past, we expect to find more differences caused by model specifications in the distant past. Many recent parameters are largely consistent across each of the models, showing that inferences of recent history are largely robust to the details of population structure in the distant past. The model that provides a very poor fit to the data (the single origin model) shows stronger model specification effects even for recent parameters (e.g., Mende population sizes, and some migration rates).

7.6 Batch effects from merged data biases inference

Merging samples from multiple independently called datasets can cause systematic biases in computed diversity statistics (i.e., batch effects). In order to explore the severity of batch effects on the LD statistics used in our inferences, we took advantage of multiple independently sequenced and called datasets using the same individuals from the Thousand Genomes project. We used the phase 3 low coverage 1000 Genomes Project Consortium *et al.* (2015) data and the high coverage resequenced data of the same individuals (Byrska-Bishop *et al.*, 2021) to 1) verify that computed diversity statistics and inferences are robust to low coverage data, and 2) demonstrate that merging data without joint variant calling can lead to substantial biases in inferred demographic parameters. We analyzed two populations, the Mende (MSL) and British (GBR), and considered a two-population isolation-with-migration model.

We computed four sets of diversity and LD statistics, one set of statistics from the jointly called high coverage data, one from the jointly called low coverage data, and one each from merged high and low coverage (MSL high coverage and GBR low coverage; and GBR high coverage and MSL low coverage). Using the measurement variances from each two-locus statistic in the high coverage data, we estimated measurement error of the other three sets of statistics (low/low, low/high, and high/low) against the high coverage data. The jointly called low coverage data provided statistics that closely matched the high coverage statistics, with each statistic within 1–2 standard errors. On the other hand, both the merged datasets provided statistics that deviated from the jointly called data, particularly for the cross-population statistics (Figure S3.

With the observation that the low and high coverage data provided broadly consistent statistics while the merged data provided inconsistent statistics, we next wanted to assess whether their differences affect model inference. To do this, we fit a two-population demographic model to the diversity and LD statistics as described in Section 3.3. The modest differences between the low and high coverage did not result in noticeable differences in the optimized model (with an MSL-GBR inferred divergence time of 60ka, consistent with previous genomics studies). On the other hand, both merged datasets provided substantially inconsistent fits, both to the high and low coverage data and to each other. Notably, the inferred divergence time is significantly older, due to the overestimation of mutational differences between populations due to batch effects.

More careful post-merging quality control may alleviate some of these discrepancies. However, low

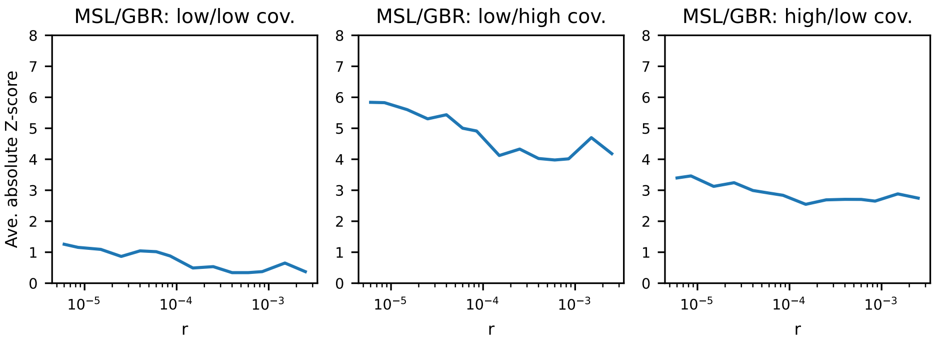

Figure S3: **Average error of statistics from low coverage and merged datasets.** Low coverage data provides allelic diversity and linkage disequilibrium measures consistent with those from high coverage data (left panel). On the other hand, merging separately called datasets can lead to large discrepancies in computed LD statistics (middle and right panels).

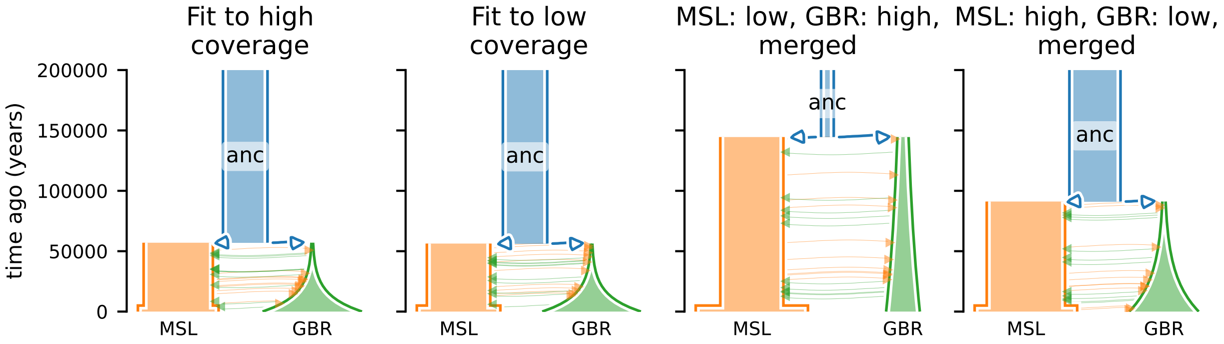

Figure S4: **Merged datasets bias demographic inferences.** The panels represent best-fit models according to different combinations of high- and low- coverage data. While statistics from low and high coverage datasets provide consistent best-fit demographic models, merged datasets result in biased LD statistics (Figure S3). These biased LD statistics lead to large downstream biases in inferred demographic models.

coverage data provides a consistent model fit to the high coverage data. Therefore, it is preferable to favor jointly called low coverage data over a mix of high coverage but separately called data, at least when using the diversity and LD statistics that we have in our analyses.

7.7 Model choice from likelihood differences

The set of models we tested with the full dataset have varying complexity, ranging from a single origin expansion to multiple stem populations connected by migration and gene flow. Thus, our models have varying numbers of parameters (21 in the simplest model, and 30 in the most heavily parameterized). Our analyses found that the more complex models (multiple stems with ongoing migration) provided the best fit to the data. To ensure that these more complex models are not simply fitting noise in our data, we simulated data under both simpler and more complex models, with same sample sizes and similar genome length to that used in this study. From these, we fit each of our models and checked that the correct model was chosen.

While simulations of datasets of this size are rapid, computing LD statistics is computationally burdensome. We simulated 500 1Mb regions under our best fit single origin, continuous migration, and merger with stem migration models. Using these simulated regions, we constructed 500 bootstrap replicate datasets by sampling genomic regions with replacement. Each of these 500 replicate sets were fit using the models

reported in the main text, and we compared log-likelihoods across each fit (Figure S5). The correct model was recovered in the all of the scenarios based on log-likelihoods alone (Table S1). We conclude that our inferences are not simply fitting statistical noise, and the observed differences in log-likelihoods indicate different abilities to capture signal present in the data.

Table S1: **Model choice from fits to simulated data.** We simulated three models, based on the best fit parameters from fits to data. Using these simulated datasets, we reinferred four models that we tested in our analyses in the main text, including simple (single origin) and complex models. Among 500 fits, we exclusively recover the simulated model, even when fitting more complex (i.e., more heavily parameterized) models to simpler simulated models (such as the single origin model).

Simulated model↓	Single origin	Cont. migration	Merger w/out stem mig.	Merger w/stem mig.
Single origin	500	0	0	0
Cont. migration	0	500	0	0
Merger w/stem mig.	0	0	0	500

The single origin model is nested within the more complex models (with migration from the additional stem population fixed to zero), those more complex models can always recover the log-likelihood found by the single origin model. We therefore explored the broader parameter space that included gene flow from the additional stem population. In these fits, the optimization converged either back to the nested single origin model (in which case the single origin model was chosen), or it found a local optimum with lower log-likelihood than the simpler model (see first panel in Figure S5).

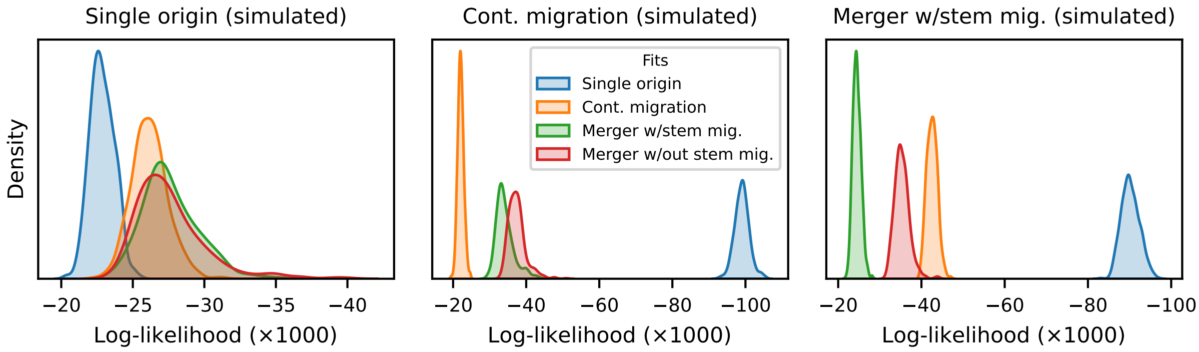

Figure S5: **Log-likelihoods of fits of four models to simulated data.** In three scenarios, a simple single origin model and our preferred continuous migration and merger with stem migration models, we simulated 500 replicates of 500 Mb with the same populations and sample sizes from our reported best fit models. We then fit the four models reported in the main text to each of these simulated datasets and compared log-likelihoods. In each case, the simulated model is correctly chosen, even under the single origin scenario. We conclude our more complex models (with more parameters) are not simply fitting statistical noise.

7.8 Allowing for additional migrations

In preliminary analyses, we found that inferred migration rates between the Nama and non-East African groups were orders of magnitude smaller than migration rates between other populations included in our inferences. Therefore, to reduce the number of parameters needing to be fit we fixed those rates to zero in subsequent fits, and our reported best-fit models did not include migration between the Mende and Eurasians, nor between the Nama and Eurasian populations (aside from recent colonial-period admixture). To assess the impact of this choice, we re-inferred four of our reported models (single origin, continuous migration, and

merger with and without stem migration), while allowing for additional parameters to include continuous, symmetric migration for the duration of the coexistence of the Nama and Mende of the Nama and British.

Indeed, the inferred migration rates for both of these pairs of populations were small, with $2N_em \ll 1$ in all cases. In the single origin model, the Mende-British migration rate was 1.04E-6 ($2N_em = 0.02$) and the Nama-British rate was 2.67E-6 ($2N_em = 0.05$). In the continuous migration model, these rates were 2.69E-6 ($2N_em = 0.038$) and 3.38E-6 ($2N_em = 0.048$), resp. In the merger without stem migration model, these rates were 2.96E-6 ($2N_em = 0.064$) and 5.46E-6 ($2N_em = 0.119$), resp. And in the merger with stem migration model, these rates were 2.64E-6 ($2N_em = 0.055$) and 3.19E-6 ($2N_em = 0.066$), resp. In comparison with other migration rates, with $2N_em \gtrsim 1$, these migrations have negligible impact on underlying statistics. The fits to other parameters were negligibly different from those reported with these migrations fixed to zero, and log-likelihoods were unimproved.

7.9 Replacing the reference population for statistics normalization

We used observed π_2 and pairwise diversity in the Mende to normalize two- and one-locus statistics (see Section 2.1). To confirm that this choice of normalization did not impact inferred model parameters, we reran each of our reported models using data normalized by π_2 and pairwise diversity in the Gumuz. As expected, the best fit parameters from fits using the Gumuz-normalized data were very close to those reported in Tables S3–S7, and they were all well within the reported confidence intervals. Our results are therefore unlikely to be sensitive to this choice of normalization.

Reinferred demographic models from this and preceding sections are reported in Demes format (Gower *et al.*, 2022) as a supplemental archive.

8 Mutation versus recombination rates

Prior studies primarily relied on the mutation clock in order to date events such as population divergence, and uncertainty about the mutation rate translates in uncertainty in timing estimates (e.g., Moorjani *et al.* (2016a)). Recent whole genome sequencing studies from non-pathological pedigrees have typically estimated mutation rates to be $1.2-1.3\times10^{-8}$ per site per generation (Sasani *et al.*, 2019; Tian *et al.*, 2019). A rate of 1.3×10^{-8} also approximates the rate predicted by a mutational model from gnomAD (Karczewski *et al.*, 2020) in the ~650Mb of intergenic regions selected for demographic inference in this study. `moments-LD` differs from other approaches by relying on a recombination clock, which has been argued to be more robust to estimation than a mutational clock (Moorjani *et al.*, 2016b).

From our recombination-rate-calibrated demographic models, we can estimate the mutation rates needed to fit patterns of pairwise diversity. With our best-fit model (merger-with-stem-migration, Table S7), a mutation rate of 1.33×10^{-8} recovers the observed diversity within and between populations, closely matching the average mutation rate estimated from the gnomAD mutation model. The other inferred demographic models (Tables S3–S6) would require mutation rates of $1.75-1.90\times10^{-8}$ to match pairwise diversity.

References

1000 Genomes Project Consortium, A. Auton, L. D. Brooks, R. M. Durbin, E. P. Garrison, *et al.*, 2015 A global reference for human genetic variation. Nature **526**: 68–74.

Alexander, D. H., and K. Lange, 2011 Enhancements to the ADMIXTURE algorithm for individual ancestry estimation. BMC Bioinformatics **12**: 246.

Alexander, D. H., J. Novembre, and K. Lange, 2009 Fast model-based estimation of ancestry in unrelated individuals. Genome Res. **19**: 1655–1664.

Baumdicker, F., G. Bisschop, D. Goldstein, G. Gower, A. P. Ragsdale, *et al.*, 2022 Efficient ancestry and mutation simulation with msprime 1.0. Genetics **220**: iyab229.

Bergström, A., S. A. McCarthy, R. Hui, M. A. Almarri, Q. Ayub, *et al.*, 2020 Insights into human genetic variation and population history from 929 diverse genomes. Science **367**.

Bergström, A., C. Stringer, M. Hajdinjak, E. M. L. Scerri, and P. Skoglund, 2021 Origins of modern human ancestry. Nature **590**: 229–237.

Beyer, R. M., M. Krapp, A. Eriksson, and A. Manica, 2021 Climatic windows for human migration out of Africa in the past 300,000 years. Nat. Commun. **12**: 1–10.

Bhatia, G., N. Patterson, S. Sankararaman, and A. L. Price, 2013 Estimating and interpreting FST: the impact of rare variants. Genome Res. **23**: 1514–1521.

Boaretto, E., M. Hernandez, M. Goder-Goldberger, V. Aldeias, L. Regev, *et al.*, 2021 The absolute chronology of Boker Tachtit (Israel) and implications for the Middle to Upper Paleolithic transition in the Levant. Proc. Natl. Acad. Sci. U. S. A. **118**.

Byrska-Bishop, M., U. S. Evani, X. Zhao, A. O. Basile, H. J. Abel, *et al.*, 2021 High coverage whole genome sequencing of the expanded 1000 Genomes Project cohort including 602 trios. bioRxiv : 2021.02.06.430068.

Chen, H., R. E. Green, S. Pääbo, and M. Slatkin, 2007 The joint allele-frequency spectrum in closely related species. Genetics **177**: 387–398.

Chen, L., A. B. Wolf, W. Fu, L. Li, and J. M. Akey, 2020 Identifying and Interpreting Apparent Neanderthal Ancestry in African Individuals. Cell **180**: 677–687.e16.

Chevrier, B., É. Huysecom, S. Soriano, M. Rasse, L. Lespez, *et al.*, 2018 Between continuity and discontinuity: An overview of the West African Paleolithic over the last 200,000 years. Quat. Int. **466**: 3–22.

Danecek, P., J. K. Bonfield, J. Liddle, J. Marshall, V. Ohan, *et al.*, 2021 Twelve years of SAMtools and BCFtools. Gigascience **10**.

Delaneau, O., J.-F. Zagury, and J. Marchini, 2013 Improved whole-chromosome phasing for disease and population genetic studies. Nat. Methods **10**: 5–6.

DePristo, M. A., E. Banks, R. Poplin, K. V. Garimella, J. R. Maguire, *et al.*, 2011 A framework for variation discovery and genotyping using next-generation DNA sequencing data. Nat. Genet. **43**: 491–498.

Devièse, T., I. Karavanić, D. Comeskey, C. Kubiak, P. Korlević, *et al.*, 2017 Direct dating of Neanderthal remains from the site of Vindija Cave and implications for the Middle to Upper Paleolithic transition. Proc. Natl. Acad. Sci. U. S. A. **114**: 10606–10611.

Durvasula, A., and S. Sankararaman, 2020 Recovering signals of ghost archaic introgression in African populations. Sci Adv **6**: eaax5097.

Fewlass, H., S. Talamo, L. Wacker, B. Kromer, T. Tuna, *et al.*, 2020 A 14C chronology for the middle to upper palaeolithic transition at Bacho Kiro Cave, Bulgaria. Nature Ecology & Evolution **4**: 794–801.

Fu, Q., H. Li, P. Moorjani, F. Jay, S. M. Slepchenko, *et al.*, 2014 Genome sequence of a 45,000-year-old modern human from western Siberia. Nature **514**: 445–449.

Gopalan, S., R. E. W. Berl, J. W. Myrick, Z. H. Garfield, A. W. Reynolds, *et al.*, 2022 Hunter-gatherer genomes reveal diverse demographic trajectories during the rise of farming in Eastern Africa. Curr. Biol. **32**: 1852–1860.e5.

Gower, G., A. P. Ragsdale, R. N. Gutenkunst, M. Hartfield, E. Noskova, *et al.*, 2022 Demes: a standard format for demographic models. bioRxiv : 2022.05.31.494112.

Groucutt, H. S., M. D. Petraglia, G. Bailey, E. M. L. Scerri, A. Parton, *et al.*, 2015 Rethinking the dispersal of Homo sapiens out of Africa. Evol. Anthropol. **24**: 149–164.

Gurdasani, D., T. Carstensen, F. Tekola-Ayele, L. Pagani, I. Tachmazidou, *et al.*, 2015 The African Genome Variation Project shapes medical genetics in Africa. Nature **517**: 327–332.

Harris, K., and R. Nielsen, 2014 Error-prone polymerase activity causes multinucleotide mutations in humans. Genome Res. **24**: 1445–1454.

Henn, B. M., C. Gignoux, A. A. Lin, P. J. Oefner, P. Shen, *et al.*, 2008 Y-chromosomal evidence of a pastoralist migration through Tanzania to southern Africa. Proc. Natl. Acad. Sci. U. S. A. **105**: 10693–10698.

Hernandez, R. D., S. H. Williamson, and C. D. Bustamante, 2007 Context dependence, ancestral misidentification, and spurious signatures of natural selection. Mol. Biol. Evol. **24**: 1792–1800.

Hershkovitz, I., O. Marder, A. Ayalon, M. Bar-Matthews, G. Yasur, *et al.*, 2015 Levantine cranium from Manot Cave (Israel) foreshadows the first European modern humans. Nature **520**: 216–219.

Heydari-Guran, S., S. Benazzi, S. Talamo, E. Ghasidian, N. Hariri, *et al.*, 2021 The discovery of an in situ Neanderthal remain in the Bawa Yawan Rockshelter, West-Central Zagros Mountains, Kermanshah. PLoS One **16**: e0253708.

Hill, W. G., and A. Robertson, 1968 Linkage disequilibrium in finite populations. Theor. Appl. Genet. **38**: 226–231.

Hublin, J.-J., 2017 The last Neanderthal. Proc. Natl. Acad. Sci. U. S. A. **114**: 10520–10522.

Hublin, J.-J., N. Sirakov, V. Aldeias, S. Bailey, E. Bard, *et al.*, 2020 Initial Upper Palaeolithic Homo sapiens from Bacho Kiro Cave, Bulgaria. Nature **581**: 299–302.

International HapMap Consortium, K. A. Frazer, D. G. Ballinger, D. R. Cox, D. A. Hinds, *et al.*, 2007 A second generation human haplotype map of over 3.1 million SNPs. Nature **449**: 851–861.

Karczewski, K. J., L. C. Francioli, G. Tiao, B. B. Cummings, J. Alföldi, *et al.*, 2020 The mutational constraint spectrum quantified from variation in 141,456 humans. Nature **581**: 434–443.

Kelleher, J., A. M. Etheridge, and G. McVean, 2016 Efficient Coalescent Simulation and Genealogical Analysis for Large Sample Sizes. PLoS Comput. Biol. **12**: e1004842.

Kuhn, S. L., and N. Zwyns, 2014 Rethinking the initial Upper Paleolithic. Quat. Int. **347**: 29–38.

Lipson, M., 2020 Applying f4 -statistics and admixture graphs: Theory and examples. Mol. Ecol. Resour. **20**: 1658–1667.

Mallick, S., H. Li, M. Lipson, I. Mathieson, M. Gymrek, *et al.*, 2016 The Simons Genome Diversity Project: 300 genomes from 142 diverse populations. Nature **538**: 201–206.

Mellars, P., 2011 The earliest modern humans in Europe. `http://dx.doi.org/10.1038/479483a`. Accessed: 2023-1-16.

Miles, A., P. i. Bot, M. R., P. Ralph, N. Harding, *et al.*, 2021 cggh/scikit-allel: v1.3.3.

Momigliano, P., A.-B. Florin, and J. Merilä, 2021 Biases in Demographic Modeling Affect Our Understanding of Recent Divergence. Mol. Biol. Evol. **38**: 2967–2985.

Moorjani, P., Z. Gao, and M. Przeworski, 2016a Human Germline Mutation and the Erratic Evolutionary Clock. PLoS Biol. **14**: e2000744.

Moorjani, P., S. Sankararaman, Q. Fu, M. Przeworski, N. Patterson, *et al.*, 2016b A genetic method for dating ancient genomes provides a direct estimate of human generation interval in the last 45,000 years. Proc. Natl. Acad. Sci. U. S. A. **113**: 5652–5657.

Niang, K., J. Blinkhorn, M. Ndiaye, M. Bateman, B. Seck, *et al.*, 2020 The Middle Stone Age occupations of Tiémassas, coastal West Africa, between 62 and 25 thousand years ago. Journal of Archaeological Science: Reports **34**: 102658.

Ohta, T., and M. Kimura, 1971 Linkage disequilibrium between two segregating nucleotide sites under the steady flux of mutations in a finite population. Genetics **68**: 571–580.

Pagani, L., S. Schiffels, D. Gurdasani, P. Danecek, A. Scally, *et al.*, 2015 Tracing the route of modern humans out of Africa by using 225 human genome sequences from Ethiopians and Egyptians. Am. J. Hum. Genet. **96**: 986–991.

Pearson, O. M., 2008 Statistical and biological definitions of “anatomically modern” humans: Suggestions for a unified approach to modern morphology. Evol. Anthropol. **17**: 38–48.

Pedregosa, Varoquaux, Gramfort, Michel, Thirion, *et al.*, 2011 Scikit-learn: Machine learning in Python. J. Mach. Learn. Res. **12**: 2825–2830.

Peter, B. M., 2016 Admixture, Population Structure, and F-Statistics. Genetics **202**: 1485–1501.

Plagnol, V., and J. D. Wall, 2006 Possible ancestral structure in human populations. PLoS Genet. **2**: e105.

Prüfer, K., C. de Filippo, S. Grote, F. Mafessoni, P. Korlević, *et al.*, 2017 A high-coverage Neandertal genome from Vindija Cave in Croatia. Science **358**: 655–658.

Prüfer, K., C. Posth, H. Yu, A. Stoessel, M. A. Spyrou, *et al.*, 2021 A genome sequence from a modern human skull over 45,000 years old from Zlatý kůň in Czechia.

Ragsdale, A. P., and S. Gravel, 2019 Models of archaic admixture and recent history from two-locus statistics. PLoS Genet. **15**: e1008204.

Ragsdale, A. P., and S. Gravel, 2020 Unbiased Estimation of Linkage Disequilibrium from Unphased Data. Mol. Biol. Evol. **37**: 923–932.

Ragsdale, A. P., C. Moreau, and S. Gravel, 2018 Genomic inference using diffusion models and the allele frequency spectrum. Curr. Opin. Genet. Dev. **53**: 140–147.

Reilly, P. F., A. Tjahjadi, S. L. Miller, J. M. Akey, and S. Tucci, 2022 The contribution of Neanderthal introgression to modern human traits. Curr. Biol. **32**: R970–R983.

Relethford, J. H., 1994 Craniometric variation among modern human populations. Am. J. Phys. Anthropol. **95**: 53–62.

Sankararaman, S., N. Patterson, H. Li, S. Pääbo, and D. Reich, 2012 The date of interbreeding between Neandertals and modern humans. PLoS Genet. **8**: e1002947.

Sasani, T. A., B. S. Pedersen, Z. Gao, L. Baird, M. Przeworski, *et al.*, 2019 Large, three-generation human families reveal post-zygotic mosaicism and variability in germline mutation accumulation. Elife **8**.

Sawyer, S. A., and D. L. Hartl, 1992 Population genetics of polymorphism and divergence. Genetics **132**: 1161–1176.

Schlebusch, C. M., P. Sjödin, G. Breton, T. Günther, T. Naidoo, *et al.*, 2020 Khoe-San Genomes Reveal Unique Variation and Confirm the Deepest Population Divergence in Homo sapiens. Mol. Biol. Evol. **37**: 2944–2954.

Speidel, L., M. Forest, S. Shi, and S. R. Myers, 2019 A method for genome-wide genealogy estimation for thousands of samples. Nat. Genet. **51**: 1321–1329.

Tennessen, J. A., A. W. Bigham, T. D. O'Connor, W. Fu, E. E. Kenny, *et al.*, 2012 Evolution and Functional Impact of Rare Coding Variation from Deep Sequencing of Human Exomes. Science **337**: 64–69.

Tian, X., B. L. Browning, and S. R. Browning, 2019 Estimating the Genome-wide Mutation Rate with Three-Way Identity by Descent. Am. J. Hum. Genet. **105**: 883–893.

Uren, C., M. Kim, A. R. Martin, D. Bobo, C. R. Gignoux, *et al.*, 2016 Fine-Scale Human Population Structure in Southern Africa Reflects Ecogeographic Boundaries. Genetics **204**: 303–314.

Valladas, H., J. L. Joron, G. Valladas, B. Arensburg, O. Bar-Yosef, *et al.*, 1987 Thermoluminescence dates for the Neanderthal burial site at Kebara in Israel. Nature **330**: 159–160.

van Eeden, G., C. Uren, E. Pless, M. Mastoras, G. D. van der Spuy, *et al.*, 2022 The recombination landscape of the Khoe-San likely represents the upper limits of recombination divergence in humans. Genome Biol. **23**: 1–14.

Virtanen, P., R. Gommers, T. E. Oliphant, M. Haberland, T. Reddy, *et al.*, 2020 SciPy 1.0: fundamental algorithms for scientific computing in Python. Nat. Methods **17**: 261–272.

von Cramon-Taubadel, N., 2009 Congruence of individual cranial bone morphology and neutral molecular affinity patterns in modern humans. Am. J. Phys. Anthropol. **140**: 205–215.

Weaver, T. D., 2016 Estimators for QST and coalescence times. Ecol. Evol. **6**: 7783–7786.

Weaver, T. D., C. C. Roseman, and C. B. Stringer, 2008 Close correspondence between quantitative- and molecular-genetic divergence times for Neandertals and modern humans. Proc. Natl. Acad. Sci. U. S. A. **105**: 4645–4649.

Whitlock, M. C., 1999 Neutral additive genetic variance in a metapopulation. Genet. Res. **74**: 215–221.

Yang, M. A., A.-S. Malaspinas, E. Y. Durand, and M. Slatkin, 2012 Ancient structure in Africa unlikely to explain Neanderthal and non-African genetic similarity. Mol. Biol. Evol. **29**: 2987–2995.

Yousefi, M., S. Heydari-Guran, A. Kafash, and E. Ghasidian, 2020 Species distribution models advance our knowledge of the Neanderthals' paleoecology on the Iranian Plateau.

Zwyns, N., 2021 The Initial Upper Paleolithic in Central and East Asia: Blade Technology, Cultural Transmission, and Implications for Human Dispersals.

Supplementary tables and figures

Table S2: **Fixed parameters in inferred demographic models.** Many of the fixed parameters are specific to the Neanderthal branch, are constrained by known history, or were consistently fit across multiple model parameterizations. We assumed a generation time of 29 years throughout. See Section 3.1 for details.

Parameter	Description	Value
$T_{MH-Neand}$	The time of the split between branches leading to Neanderthals and humans	550ka
T_{NI-Vin}	The time of the split between branches leading to the Vindija sample and the Neanderthal population that mixed with expanding humans	80ka
T_{Vin}	The sample time of the Vindija Neanderthal individual	50ka
$T_{NI \to EUR}$	The timing of admixture from Neanderthals to humans	45ka
$f_{NI \to EUR}$	The proportion of admixture from Neanderthals to humans	0.015
T_{E-W}	The split time of East and West African populations	60ka
T_{GBR}	The split time of Eurasian and East African populations	50ka
$T_{GBR \to EA}$	The time of the back-to-Africa admixture event, giving rise to the Amhara/Oromo branch	12ka
T_{expan}	The expansion time of the Mende and contraction time of the Gumuz	5ka
$T_{EA \to Nama}$	The admixture time of East African pastoralists to the Nama	67 gen.
$T_{GBR \to Nama}$	The admixture time of Europeans to the Nama	10 gen.
T_{bottle}	The bottleneck time of the Nama population	9 gen.

Table S3: **Best-fit parameters from the single-origin model.** This model includes a single stem population that splits into contemporary human groups. Fixed parameters are specified in Table S2. Inferred values are scaled to physical units assuming a generation time of 29 years. This model gave a log-likelihood of -189,271.

Parameter	Description	Value	95% CI
N_e	Ancestral effective population size	9860	9600–10090
N_{MH}	Size of human lineage between Neanderthal and Nama splits	20850	20640–21220
N_{Nama_0}	Initial Nama size	10170	9950–10250
N_{Nama_F}	Final Nama size	222	217–226
N_{MSL_0}	Initial Mende size	17280	16770–17490
N_{MSL_F}	Final Mende size	16730	16370–17060
N_{EA}	Size of East African branch	7130	6940–7130
N_{Gumuz_F}	Final Gumuz size	3800	3690–3900
N_{EP}	East African agriculturist size	12990	12720–13140
N_{GBR_0}	Initial British size	845	836–862
N_{GBR_F}	Final British size	12140	11870–12300
N_{Neand}	Neanderthal size	1850	1650–1990
T_{Nama}	Nama split time (years)	110900	107800–112200
$m_{Nama-MSL}$	Nama–Mende symmetric migration rate	2.83×10^{-5}	2.61×10^{-5}–3.02×10^{-5}
$m_{Nama-EA}$	Nama–East Africa symmetric migration rate	4.98×10^{-5}	4.82×10^{-5}–5.31×10^{-5}
m_{MSL-EA}	Mende–East Africa migration rate	18.7×10^{-5}	18.7×10^{-5}–19.2×10^{-5}
m_{EA-GBR}	East Africa–Europe migration rate	4.38×10^{-5}	4.13×10^{-5}–4.54×10^{-5}
m_{EA-EA}	Intra-East Africa migration rate	43.8×10^{-5}	41.0×10^{-5}–44.2×10^{-5}
$f_{GBR \to EP}$	Ancestry proportion of East African agriculturalists from GBR 12ka ($1 - f$ from Gumuz)	0.661	0.657–0.671
$f_{EP \to Nama}$	Ancestry proportion from EA pastoralists to Nama 2ka	0.280	0.270–0.286
$f_{GBR \to Nama}$	Ancestry proportion from Europeans to Nama 10 generations ago	0.150	0.147–0.154

Table S4: **Best-fit parameters from the Continuous-Migration model without stem migration.** Inferred values are scaled to physical units assuming a generation time of 29 years. This model gave a log-likelihood of -126,540. This model allows for continuous migration between Stem 2 and contemporary populations, but disallows migration between Stem 2 and Stem 1, which later splits into branches leading to South, East, and West African populations.

Parameter	Description	Value	95% CIs
N_e	Ancestral effective population size	10980	10810–11260
N_{stem1}	Size of stem 1 lineage between Neanderthal and Nama splits	13980	13930–15330
N_{stem2}	Size of stem 2 lineage	10390	9780–11250
N_{Nama_0}	Initial Nama size	11240	10760–11280
N_{Nama_F}	Final Nama size	221	216–226
N_{MSL_0}	Initial Mende size	10810	10510–11090
N_{MSL_F}	Final Mende size	25080	24050–25780
N_{EA}	Size of East African branch	7130	6970–7200
N_{Gumuz_F}	Final Gumuz size	3770	2650–3860
N_{EP}	East African agriculturist size	13070	12830–13250
N_{GBR_0}	Initial British size	946	931–964
N_{GBR_F}	Final British size	11560	11320–11760
N_{Neand}	Neanderthal size	2290	2140–2450
T_{stems}	Stem split time (years)	459700	446600–463300
T_{Nama}	Nama split time (years)	145400	137300–146000
$m_{Nama-MSL}$	Nama–Mende symmetric migration rate	1.46×10^{-5}	1.36×10^{-5}–1.81×10^{-5}
$m_{Nama-EA}$	Nama–East Africa symmetric migration rate	4.22×10^{-5}	4.08×10^{-5}–4.60×10^{-5}
m_{MSL-EA}	Mende–East Africa migration rate	20.8×10^{-5}	20.7×10^{-5}–21.3×10^{-5}
m_{EA-GBR}	East Africa–Europe migration rate	4.36×10^{-5}	4.19×10^{-5}–4.58×10^{-5}
m_{EA-EA}	Intra-East Africa migration rate	35.8×10^{-5}	34.6×10^{-5}–37.0×10^{-5}
$f_{GBR \to EP}$	Ancestry proportion of East African agriculturalists from GBR 12ka ($1 - f$ from Gumuz)	0.651	0.644–0.660
$f_{EP \to Nama}$	Ancestry proportion from EA pastoralists to Nama 2ka	0.271	0.264–0.279
$f_{GBR \to Nama}$	Ancestry proportion from Europeans to Nama 10 generations ago	0.153	0.148–0.156
m_{stems}	Stem 1–stem 2 migration rate	0	fixed
$m_{stem2-Nama}$	Stem 2–Nama migration rate	5.63×10^{-5}	4.91×10^{-5}–5.92×10^{-5}
$m_{stem2-MSL}$	Stem 2–Mende migration rate	14.2×10^{-5}	12.4×10^{-5}–14.4×10^{-5}
$m_{stem2-EA}$	Stem 2–East Africa migration rate	3.82×10^{-5}	3.04×10^{-5}–3.95×10^{-5}

Table S5: **Best-fit parameters from the Continuous-Migration model.** Inferred values are scaled to physical units assuming a generation time of 29 years. This model gave a log-likelihood of -115,280.

Parameter	Description	Value	95% CIs
N_e	Ancestral effective population size	6090	4180–6530
N_{stem1}	Size of stem 1 lineage between Neanderthal and Nama splits	8080	7130–8400
N_{stem2}	Size of stem 2 lineage	12340	11330–13580
N_{Nama_0}	Initial Nama size	11860	11160–12390
N_{Nama_F}	Final Nama size	221	215–226
N_{MSL_0}	Initial Mende size	9880	9500–10210
N_{MSL_F}	Final Mende size	27730	25930–28930
N_{EA}	Size of East African branch	7460	7270–7630
N_{Gumuz_F}	Final Gumuz size	3730	3560–3810
N_{EP}	East African agriculturist size	13110	12800–13290
N_{GBR_0}	Initial British size	951	940–981
N_{GBR_F}	Final British size	11874	11490–12090
N_{Neand}	Neanderthal size	2620	2380–2750
T_{stems}	Stem split time (years)	1223000	1199000–1382000
T_{Nama}	Nama split time (years)	134200	127800–139900
$m_{Nama-MSL}$	Nama–Mende symmetric migration rate	0.92×10^{-5}	0.41×10^{-5}–1.43×10^{-5}
$m_{Nama-EA}$	Nama–East Africa symmetric migration rate	4.11×10^{-5}	3.92×10^{-5}–4.56×10^{-5}
m_{MSL-EA}	Mende–East Africa migration rate	21.3×10^{-5}	21.2×10^{-5}–21.8×10^{-5}
m_{EA-GBR}	East Africa–Europe migration rate	4.16×10^{-5}	3.77×10^{-5}–4.33×10^{-5}
m_{EA-EA}	Intra-East Africa migration rate	33.7×10^{-5}	33.0×10^{-5}–36.3×10^{-5}
$f_{GBR \to EP}$	Ancestry proportion of East African agriculturalists from GBR 12ka ($1 - f$ from Gumuz)	0.642	0.639–0.656
$f_{EP \to Nama}$	Ancestry proportion from EA pastoralists to Nama 2ka	0.255	0.245–0.263
$f_{GBR \to Nama}$	Ancestry proportion from Europeans to Nama 10 generations ago	0.155	0.151–0.160
m_{stems}	Stem 1–stem 2 migration rate	6.26×10^{-5}	6.14×10^{-5}–7.83×10^{-5}
$m_{stem2-Nama}$	Stem 2–Nama migration rate	5.85×10^{-5}	4.50×10^{-5}–7.04×10^{-5}
$m_{stem2-MSL}$	Stem 2–Mende migration rate	16.2×10^{-5}	13.8×10^{-5}–18.9×10^{-5}
$m_{stem2-EA}$	Stem 2–East Africa migration rate	3.10×10^{-5}	2.18×10^{-5}–3.65×10^{-5}

Table S6: **Best-fit parameters from the Merger-Without-Stem-Migration model.** Inferred values are scaled to physical units assuming a generation time of 29 years. This model gave a log-likelihood of -107,694.

Parameter	Description	Value	95% CIs
N_e	Ancestral effective population size	10990	10100–11290
N_{stem1}	Size of stem 1 lineage between stem 1–stem 2 split and stem 1E–stem 1S split	278	113–606
N_{stem2}	Size of stem 2 lineage	27850	26500–35060
N_{Nama_0}	Initial Nama and stem 1S size	12640	11310–12910
N_{Nama_F}	Final Nama size	227	223–238
N_{MSL_0}	Initial Mende size	11190	10280–11630
N_{MSL_F}	Final Mende size	27440	25720–30180
N_{EA}	Size of East African and stem 1E branch	9016	8680–9140
N_{Gumuz_F}	Final Gumuz size	3440	3270–3470
N_{EP}	East African agriculturist size	13680	13120–13690
N_{GBR_0}	Initial British size	922	920–963
N_{GBR_F}	Final British size	12020	11790–12430
N_{Neand}	Neanderthal size	2100	1770–2110
T_{stems}	Stem split time (years)	400300	381400–410700
T_{stem1}	Stem 1 split time into stem 1E and stem 1S (years)	281600	154300–320400
$m_{Nama-MSL}$	Nama–Mende symmetric migration rate	0.229×10^{-5}	0.205×10^{-5}–0.697×10^{-5}
$m_{Nama-EA}$	Nama–East Africa symmetric migration rate	4.15×10^{-5}	3.90×10^{-5}–4.42×10^{-5}
m_{MSL-EA}	Mende–East Africa migration rate	19.9×10^{-5}	19.8×10^{-5}–20.8×10^{-5}
m_{EA-GBR}	East Africa–Europe migration rate	3.99×10^{-5}	3.34×10^{-5}–3.95×10^{-5}
m_{EA-EA}	Intra-East Africa migration rate	35.5×10^{-5}	35.2×10^{-5}–39.6×10^{-5}
$f_{GBR \rightarrow EP}$	Ancestry proportion of East African agriculturalists from GBR 12ka ($1 - f$ from Gumuz)	0.641	0.641–0.661
$f_{EP \rightarrow Nama}$	Ancestry proportion from EA pastoralists to Nama 2ka	0.257	0.245–0.264
$f_{GBR \rightarrow Nama}$	Ancestry proportion from Europeans to Nama 10 generations ago	0.156	0.152–0.161
m_{stems}	Stem 1–stem 2 migration rate	0	fixed
T_{Nama}	Time of Nama merger event	112300	101400–116800
$f_{stem2 \rightarrow Nama}$	Proportion of stem 2 ancestry making up initial Nama lineage ($1 - f$ from stem 1S)	0.731	0.706–0.769
T_{EA}	Time of East Africa merger event	95000	94500–101400
$f_{stem2 \rightarrow EA}$	Proportion of stem 2 ancestry making up initial East Africa lineage ($1 - f$ from stem 1E)	0.522	0.510–0.564
T_{MSL}	Time of secondary admixture from stem 2 to Mende	23510	22920–25510
$f_{stem2 \rightarrow MSL}$	Proportion of ancestry from secondary stem 2 admixture to Mende	0.183	0.174–0.196

Table S7: **Best-fit parameters from the Merger-With-Stem-Migration model.** Inferred values are scaled to physical units assuming a generation time of 29 years. This model gave a log-likelihood of -101,618.

Parameter	Description	Value	Std. err.
N_e	Ancestral effective population size	9640	6410–11910
N_{stem1}	Size of stem 1 lineage between Neanderthal split and stem 1E–stem 1S split	100	100–851
N_{stem2}	Size of stem 2 lineage	24210	22130–26340
N_{Nama_0}	Initial Nama size	13440	11500–13560
N_{Nama_F}	Final Nama size	219	214–231
N_{MSL_0}	Initial Mende size	11380	9690–11290
N_{MSL_F}	Final Mende size	27760	26820–32280
N_{EA}	Size of East African Branch	9100	7970–8860
N_{Gumuz_F}	Final Gumuz size	3410	3270–3570
N_{EP}	East African agriculturist size	13510	12780–13500
N_{GBR_0}	Initial British size	958	928–1002
N_{GBR_F}	Final British size	12300	11850–12600
N_{Neand}	Neanderthal size	2520	2100–2730
T_{stems}	Stem split time (years)	1692000	695000–1785000
T_{stem1}	Stem 1S–stem 1E split time (years)	478900	275600–478300
$m_{Nama-MSL}$	Nama–Mende symmetric migration rate	0.98×10^{-5}	0.66×10^{-5}–1.57×10^{-5}
$m_{Nama-EA}$	Nama–East Africa symmetric migration rate	4.48×10^{-5}	4.45×10^{-5}–5.52×10^{-5}
m_{MSL-EA}	Mende–East Africa migration rate	19.7×10^{-5}	20.0×10^{-5}–21.7×10^{-5}
m_{EA-GBR}	East Africa–Europe migration rate	3.82×10^{-5}	3.25×10^{-5}–4.27×10^{-5}
m_{EA-EA}	Intra-East Africa migration rate	35.9×10^{-5}	33.7×10^{-5}–40.9×10^{-5}
$f_{GBR \to EP}$	Ancestry proportion of East African agriculturalists from GBR 12ka ($1-f$ from Gumuz)	0.640	0.636–0.667
$f_{EP \to Nama}$	Ancestry proportion from EA pastoralists to Nama 2ka	0.259	0.237–0.267
$f_{GBR \to Nama}$	Ancestry proportion from Europeans to Nama 10 generations ago	0.158	0.154–0.167
m_{stems}	Stem 1–stem 2 migration rate	12.6×10^{-5}	3.95×10^{-5}–12.3×10^{-5}
T_{Nama}	Time of Nama merger event	119500	101300–124600
$f_{stem2 \to Nama}$	Proportion of stem 2 ancestry making up initial Nama lineage ($1-f$ from stem 1S)	0.698	0.694–0.758
T_{EA}	Time of East Africa merger event	96300	95400–111000
$f_{stem2 \to EA}$	Proportion of stem 2 ancestry making up initial East Africa lineage ($1-f$ from stem 1E)	0.515	0.514–0.589
T_{MSL}	Time of secondary admixture from stem 2 to Mende	25120	22210–26300
$f_{stem2 \to MSL}$	Proportion of ancestry from secondary stem 2 admixture to Mende	0.187	0.182–0.211

Table S8: **Probability that a lineage sampled 500ya is found in a given branch.** Using our best-fit model (merger with stem migration), we calculated the probability that a lineage sampled in a given population (Nama, Mende, or Gumuz) is found in each branch at 10ka, 55ka, and 500ka. Lineages are sampled 500 years ago to avoid the effects of very recent admixture, such as recent European admixture in the Nama.

	Nama branch	Mende branch	Gumuz branch	Oromo/Amhara branch	British branch	Neand.	Stem 1	Stem 2
Found 10ka								
Nama	0.724	0.015	0.032	0.226	0.003			
Mende	0.003	0.881	0.058	0.058	0.001			
Gumuz	0.013	0.058	0.819	0.098	0.012			
Oromo/Amhara	0.013	0.058	0.098	0.819	0.012			
Found 55ka								
Nama	0.677	0.052	0.264			0.002		0.005
Mende	0.021	0.562	0.272			0.001		0.143
Gumuz	0.060	0.211	0.704			0.002		0.023
Oromo/Amhara	0.041	0.142	0.793			0.008		0.017
Found 500ka								
Nama						0.002	0.379	0.618
Mende						0.001	0.430	0.569
Gumuz						0.002	0.473	0.525
Oromo/Amhara						0.008	0.475	0.517

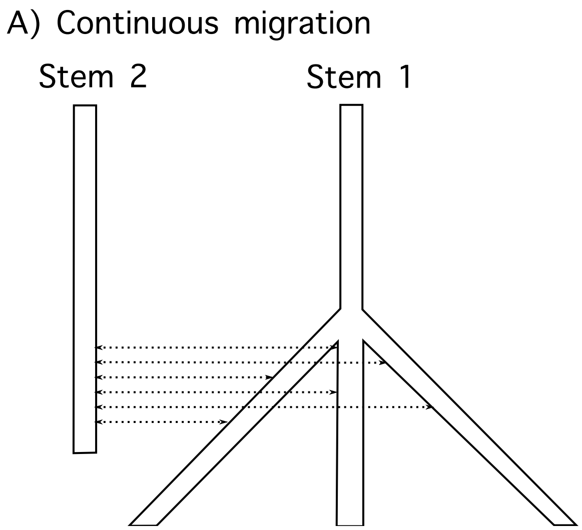

Figure S6: **Two scenarios for population rearrangement.** In our demographic models, we considered A) continuous migration models, in which one of the stem population expands (splits into contemporary populations), and the other stem population(s) has continuous symmetric migration with those populations; and B) one or more of the stem populations expands, with instantaneous pulse (or "merger") events from the other stem population, so that recent populations are formed by mergers of multiple ancestral populations.

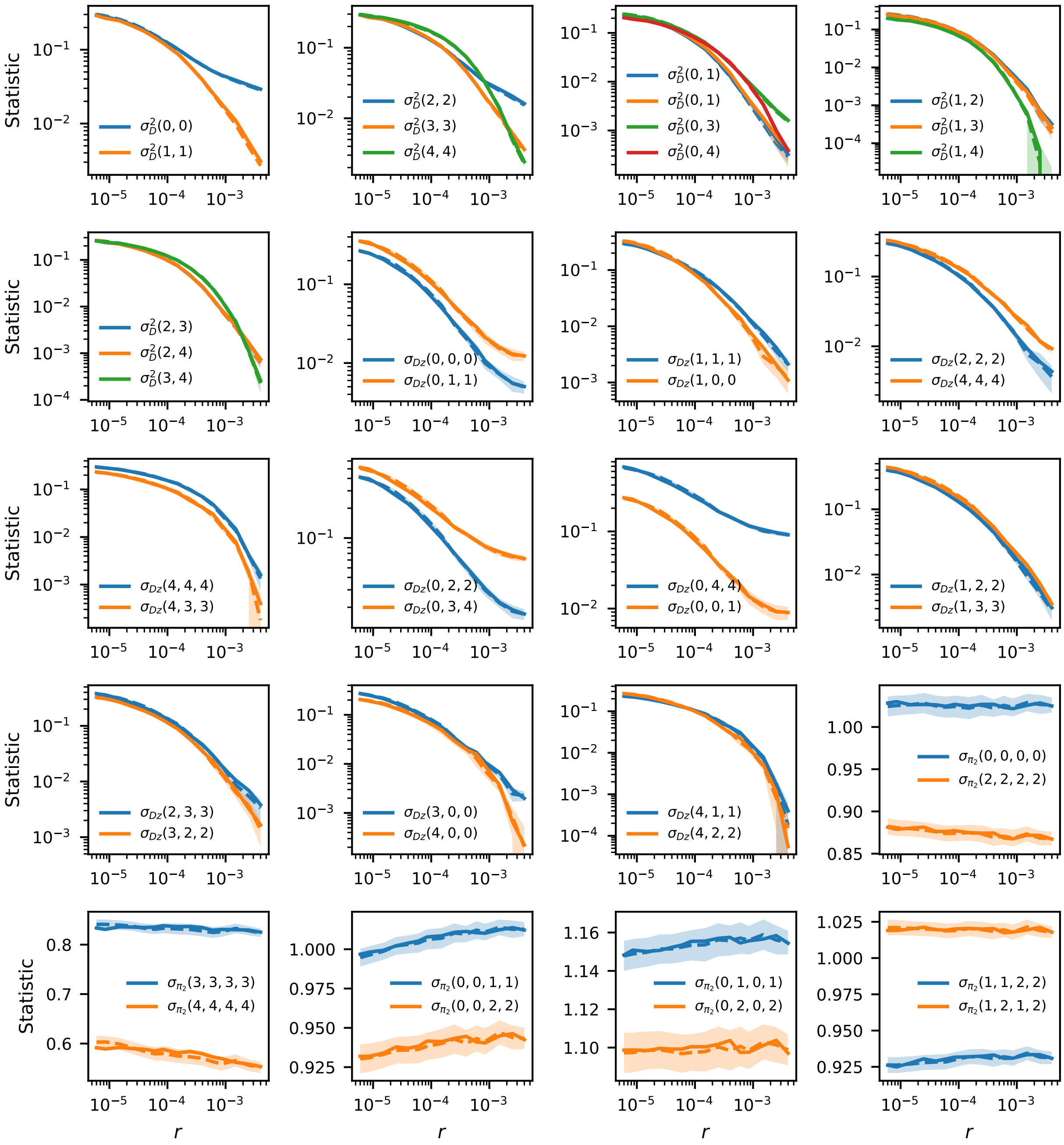

Figure S7: **Comparison of LD statistics parsed using two array-based recombination maps.** The recombination map determines genetic distances between pairs of SNPs, so using different maps could result in systematic biases in parsed statistics if the maps differ significantly. Here, the solid lines are statistics parsed using the HapMapII recombination map, and dashed lines are using the OMNI-YRI map. Shading represents 95% confidence intervals from the OMNI map. These two maps results in LD statistics that are indistinguishable from one another. Notations for each statistic are described in section 2.1 and indexed by populations (0: Nama, 1: Mende, 2: Gumuz, 3: Amhara/Oromo, 4: British).

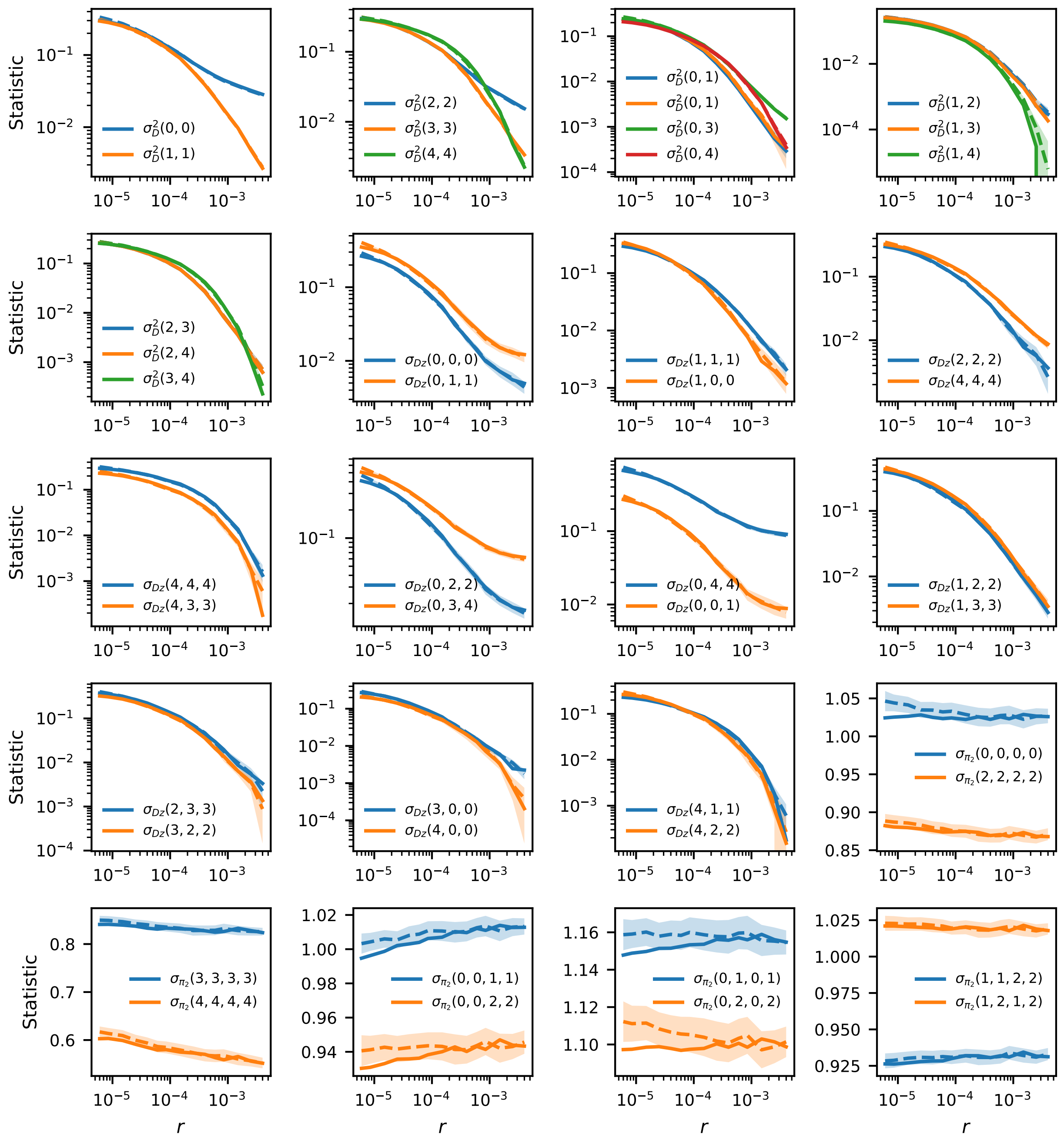

Figure S8: **Comparison of LD statistics parsed using OMNI YRI and Nama-specific recombination map.** The OMNI YRI recombination map and the Nama-inferred map (van Eeden *et al.*, 2022) differ in two primary ways: 1) the YRI and Nama have relatively highly diverged ancestries, and 2) the YRI map was inferred from low-density array data, while the Nama map was inferred from whole-genome sequencing data. Nonetheless, LD statistics computed under recombination rates from the two maps remain largely concordant, and inferences are unaffected by recombination map choice (Section 7). Shading represents 95% confidence intervals from the Nama-inferred map. Notations for each statistic are described in section 2.1, and indexes represent populations (0: Nama, 1: Mende, 2: Gumuz, 3: Amhara/Oromo, 4: British).

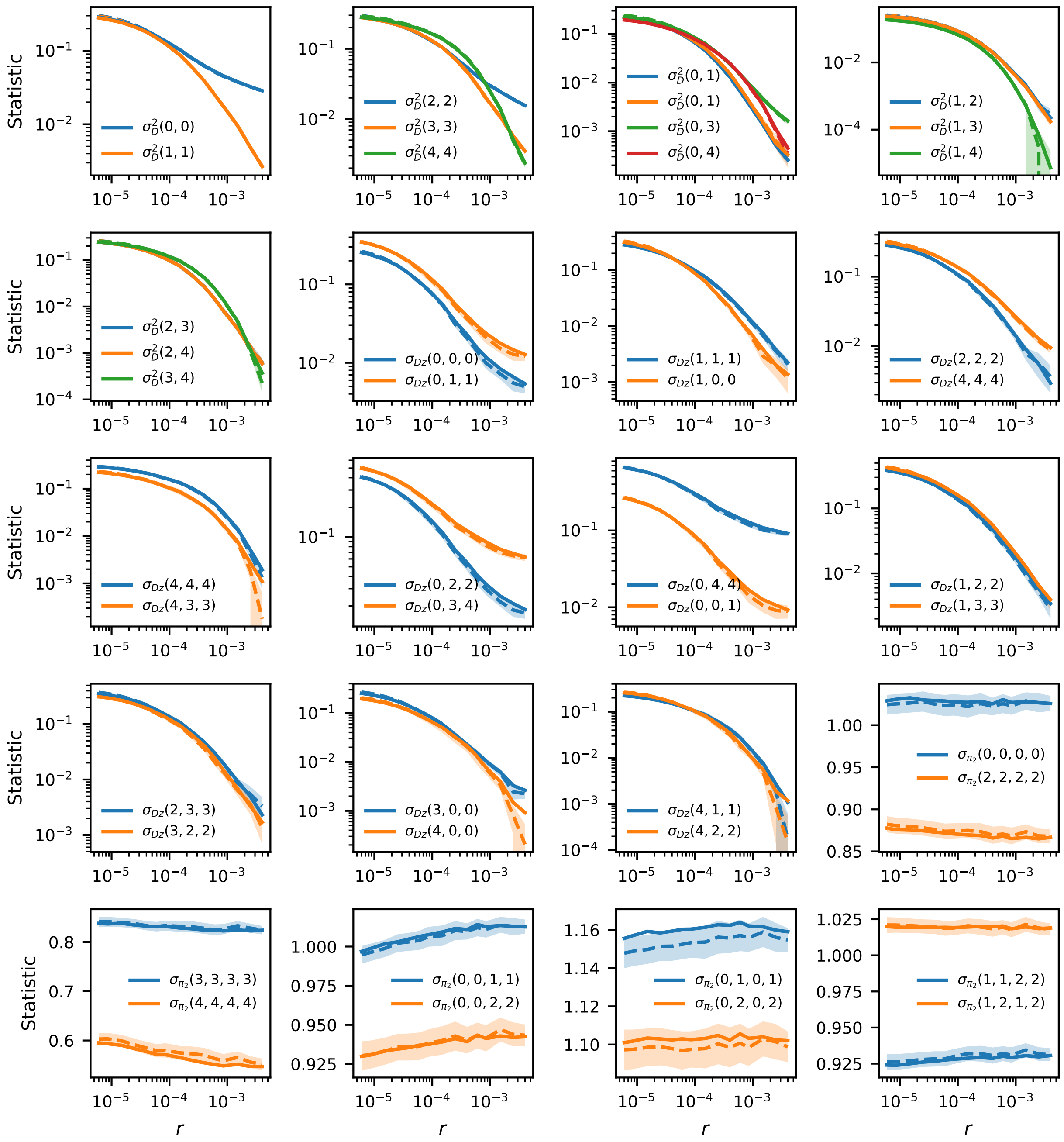

Figure S9: **Comparison of LD statistics parsed from intergenic loci and from all loci across the genome.** Statistics computed from intergenic regions (dashed lines, shown with computed standard errors as shaded regions) and those computed from all regions genome-wide (solid lines) do not show large differences. We chose to perform model inference using intergenic regions alone to reduce bias due to selection in and around protein-coding regions (Section 2.2). Notations for each statistic are described in section 2.1, and indexes represent populations (0: Nama, 1: Mende, 2: Gumuz, 3: Amhara/Oromo, 4: British).

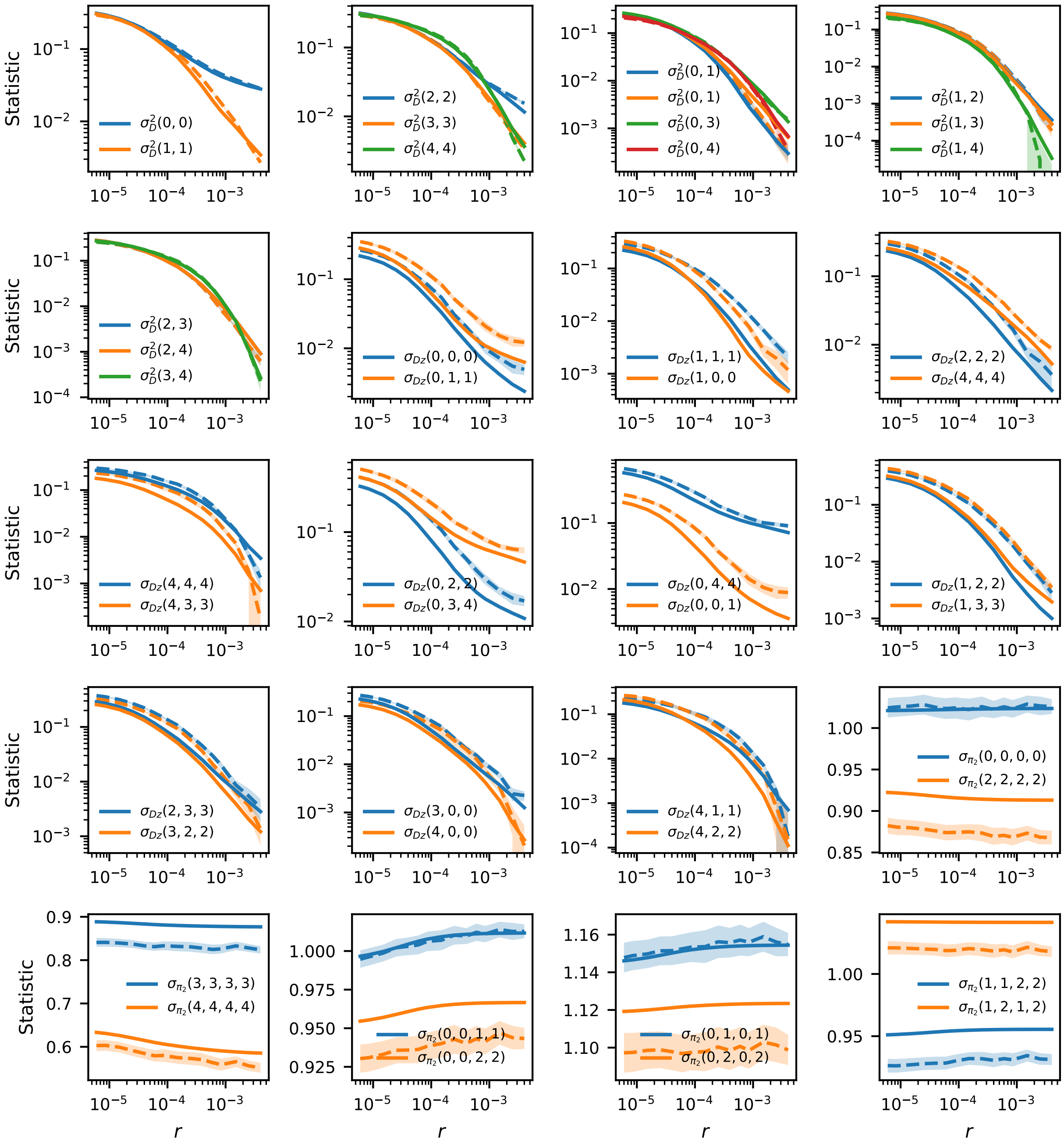

Figure S10: **Single-origin model fit to LD statistics.** Predicted vs observed two-locus statistics as a function of genetic distance for the single-origin model. Each panel represents a different set of two-locus statistics. Solid lines represent estimates using the single-origin model. Dashed lines represent observed statistics. Notation and indexing of statistics are described in the Fig. S7 caption.

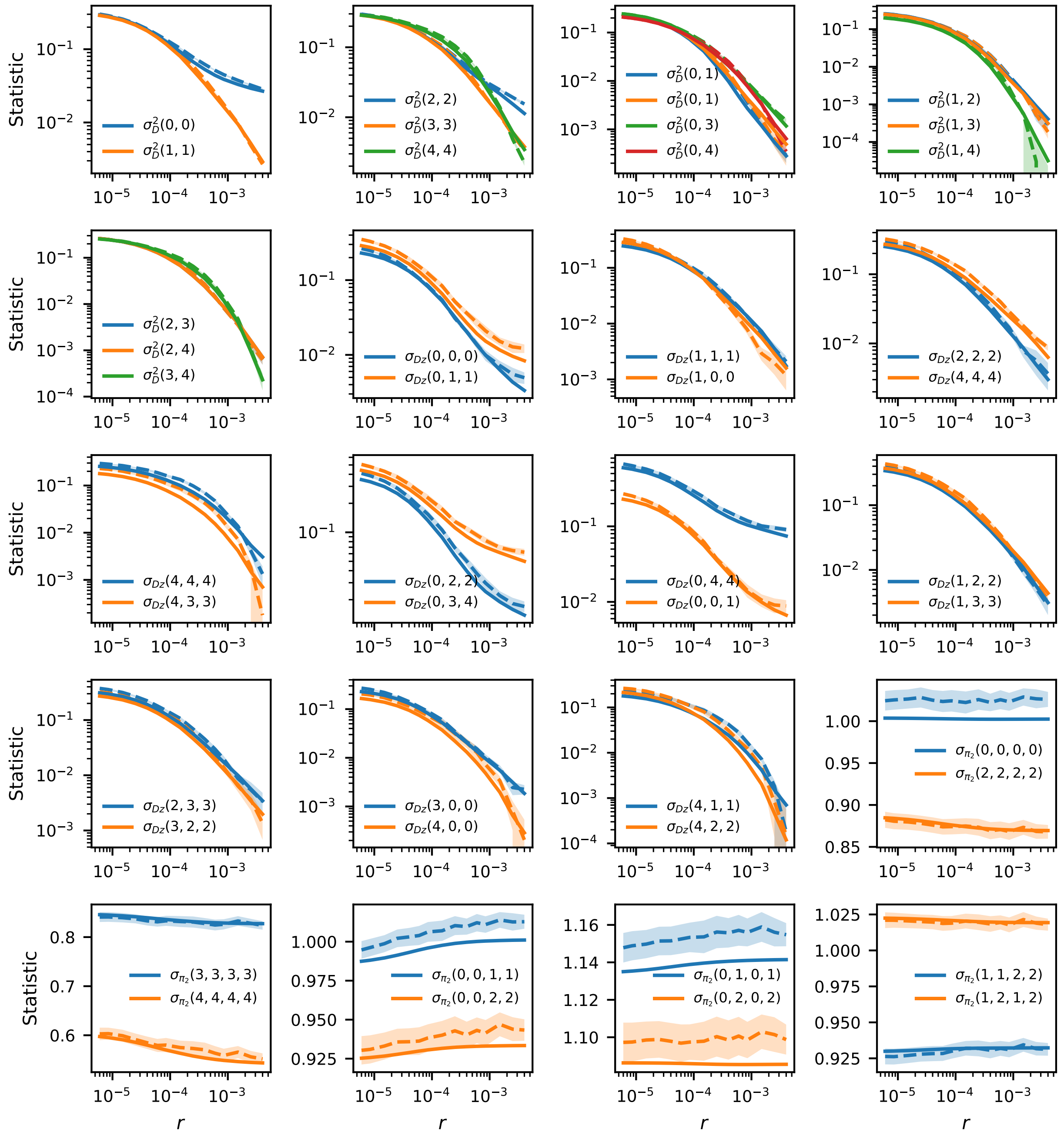

Figure S11: **Continuous-migration model fit to LD statistics.** Predicted vs observed two-locus statistics as a function of genetic distance for the continuous-migration model with migration between stems. Each panel represents a different set of two-locus statistics. Solid lines represent estimates using the continuous-migration model. Dashed lines represent observed statistics. Notation and indexing of statistics are described in the Fig. S7 caption.

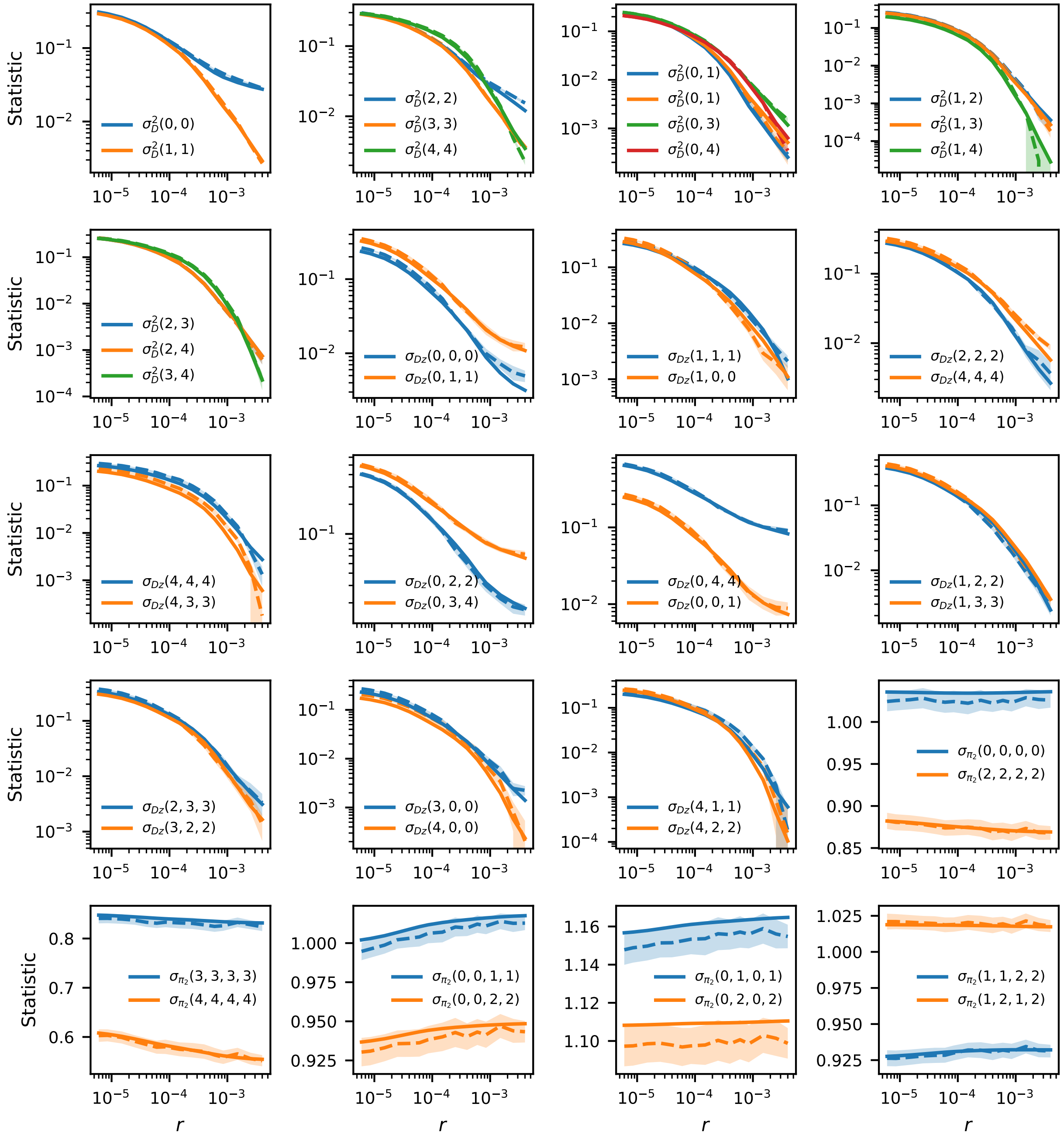

Figure S12: **Merger-without-stem-migration model fit to LD statistics.** Predicted vs observed two-locus statistics as a function of genetic distance for the merger-without-stem-migration model. Each panel represents a different set of two-locus statistics. Solid lines represent estimates using the merger-without-stem-migration model. Dashed lines represent observed statistics. Notation and indexing of statistics are described in the Fig. S7 caption.

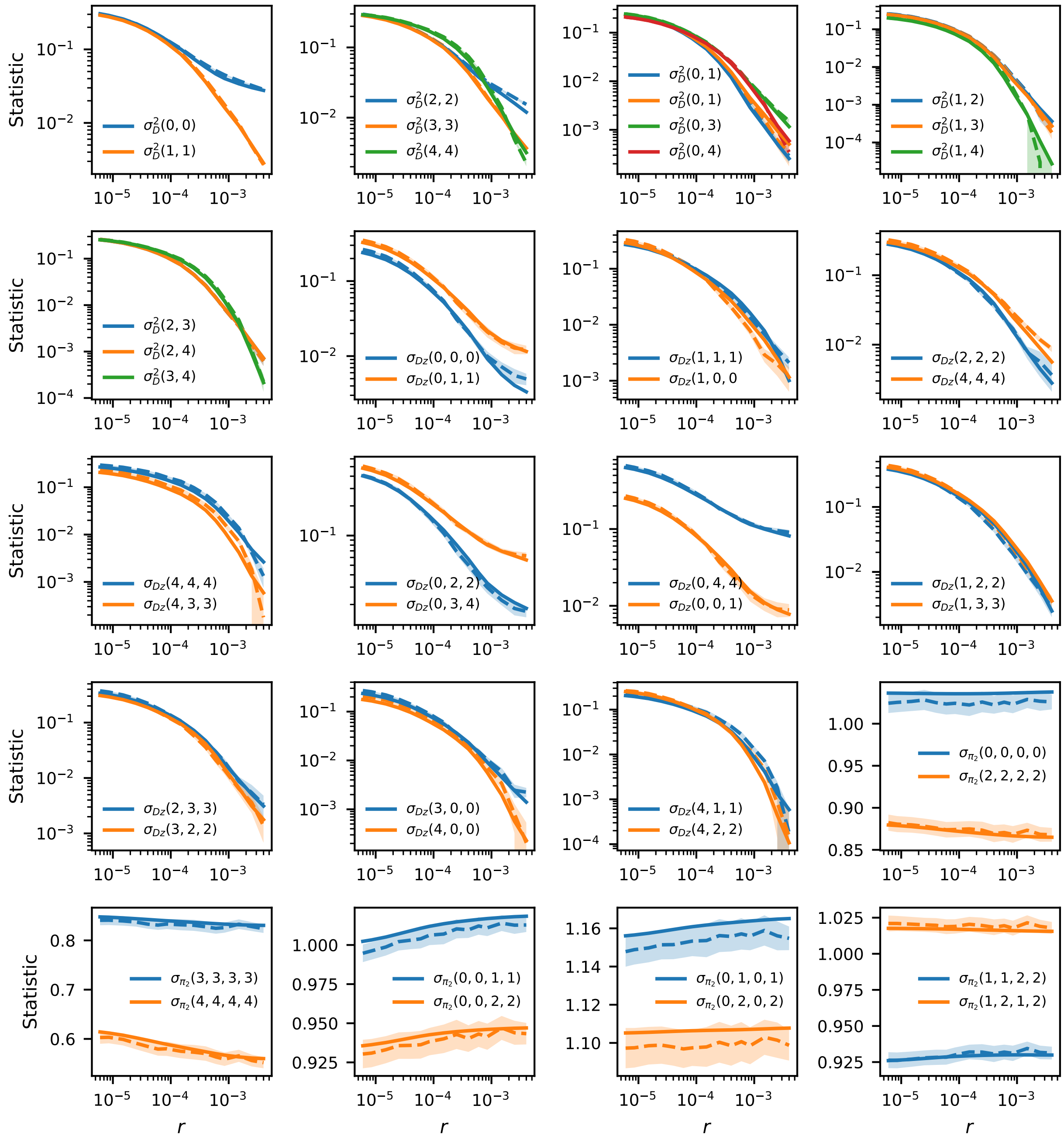

Figure S13: **Merger-with-stem-migration model fit to LD statistics.** Predicted vs observed two-locus statistics as a function of genetic distance for the merger-with-stem-migration model. Each panel represents a different set of two-locus statistics. Solid lines represent estimates using the merger-with-stem-migration model. Dashed lines represent observed statistics. Notation and indexing of statistics are described in the Fig. S7 caption.

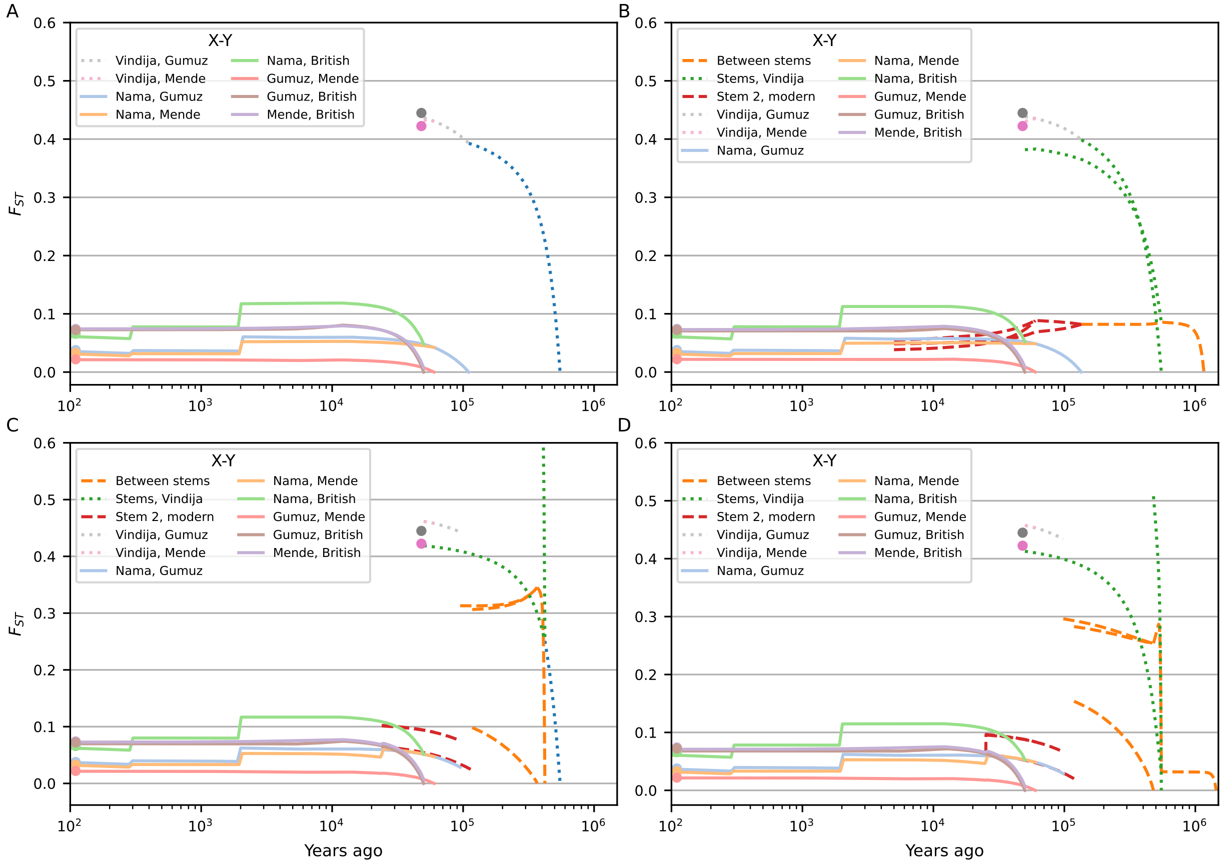

Figure S14: **Predicted F_{ST} between contemporaneous populations over time.** All models (A: single origin, B: continuous migration, C: merger without stem migration, D: merger with stem migration) match observed F_{ST} between sampled populations. The continuous-migration model predicts that human stem populations remain genetically similar, while the merger with and without stem migration models predict a period of increased F_{ST} between ancestors of contemporary humans. This is largely due to the very small inferred N_e in one of the stem branches, which leads to a rapid increase in differentiation due to drift.

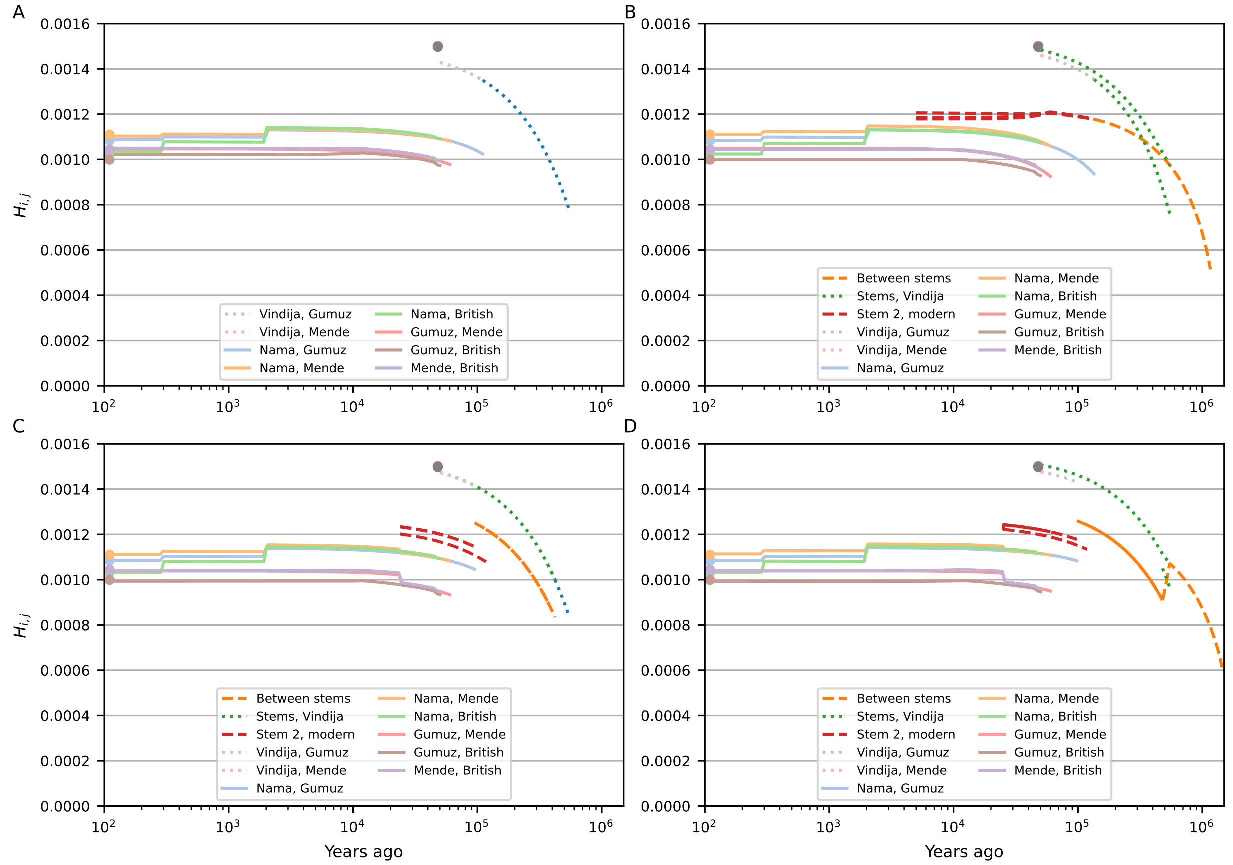

Figure S15: **Predicted pairwise differentiation between contemporaneous populations.** $H_{i,j}$ is the predicted pairwise differentiation between two populations per base-pair, using a mutation rate estimated from matching predicted to observed pairwise diversity from the intergenic data used in the fits (see Sec. 8). The single-origin model (A) shows larger deviations between observed and expected differentiation than the models with early human structure (B: continuous migration, C: merger without stem migration, D: merger with stem migration). In the merger models (C and D), while F_{ST} is large between early stems due to the sharp bottleneck in one of those branches, the pairwise differences are comparable to the continuous-migration model. Stem 1E and Stem 1S have low differentiation, as the bottleneck in Stem 1 (which they both split from) sharply reduces diversity by the time of the split. This has the effect of large F_{ST} but small $H_{i,j}$.

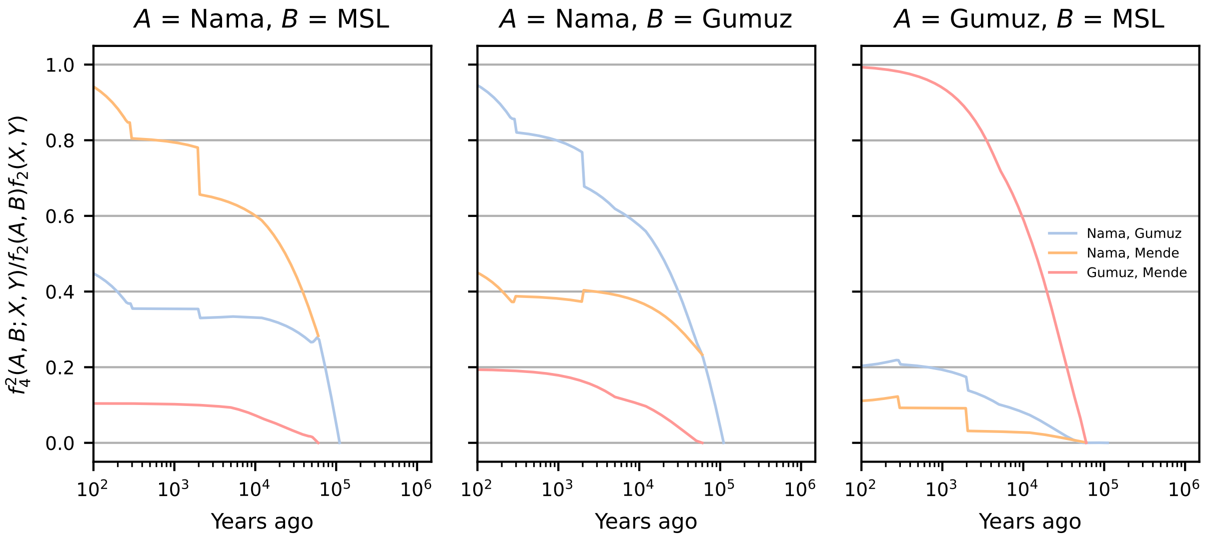

Figure S16: **Predicted f_4 between pairs of contemporary human populations and ancient branches from the single-origin model.** $f_4^2(A, B; X, Y)/f_2(A, B)f_2(X, Y)$ is interpreted as the proportion of the amount of drift between sampled populations A and B that aligns with drift between populations X and Y, sampled in the past (see Section 5.2). In the single-origin model, nearly all differentiation between the Nama, Mende, and Gumuz populations arises after the divergence events, so normalized f_4 decays to zero by the time of those divergences.

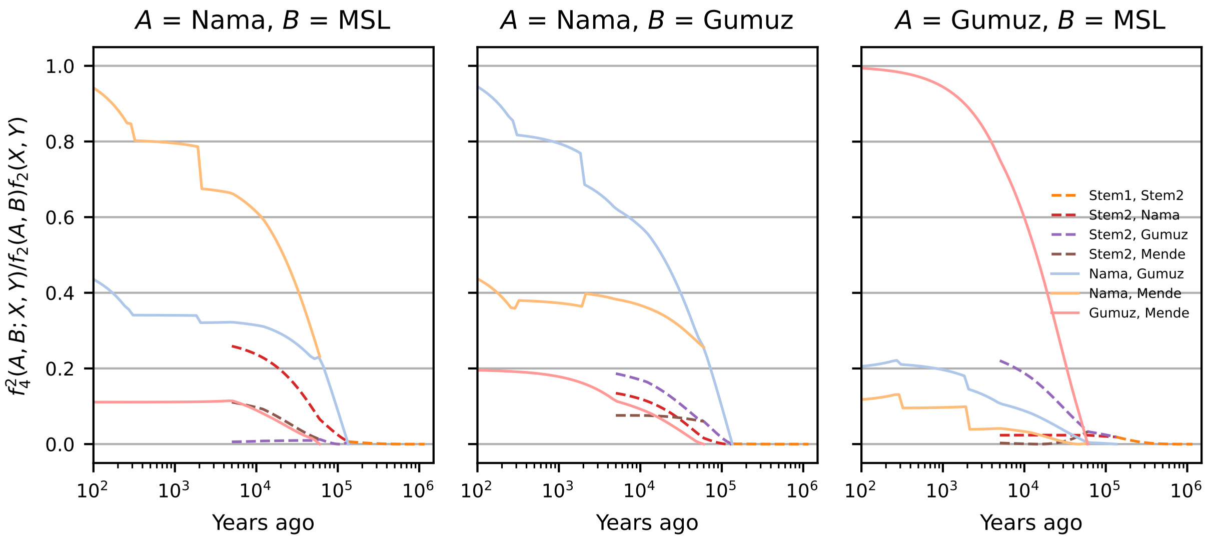

Figure S17: **Predicted f_4 between pairs of contemporary human populations and ancient branches from the continuous-migration model.** $f_4^2(A, B; X, Y)/f_2(A, B)f_2(X, Y)$ is interpreted as the proportion of the amount of drift between sampled populations A and B that aligns with drift between populations X and Y, sampled in the past (see Section 5.2). Despite population structure inferred to have extended up to 1Ma into the past, differentiation between contemporary African populations only traces back to differentiation between ancestral populations ~100-200ka, indicating that while stem populations were structured (Fig. S14), differentiation due to that ancient structure contributes little to differentiation between contemporary populations.

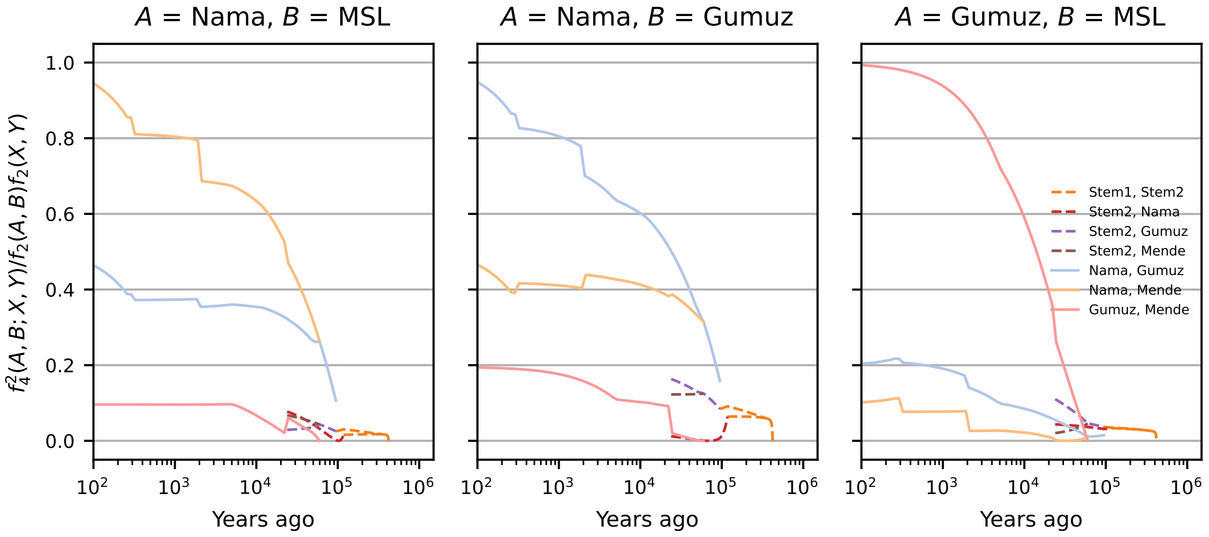

Figure S18: **Predicted f_4 between pairs of contemporary human populations and ancient branches from the merger-without-stem-migration model.** $f_4^2(A, B; X, Y)/f_2(A, B)f_2(X, Y)$ is interpreted as the proportion of the amount of drift between sampled populations A and B that aligns with drift between populations X and Y, sampled in the past (see Section 5.2). Unlike the continuous-migration model, population structure in the stems following the divergence of Neanderthal and human ancestors results in differentiation among contemporary populations that aligns with drift between the stems. In this model, migration between stem populations is disallowed.

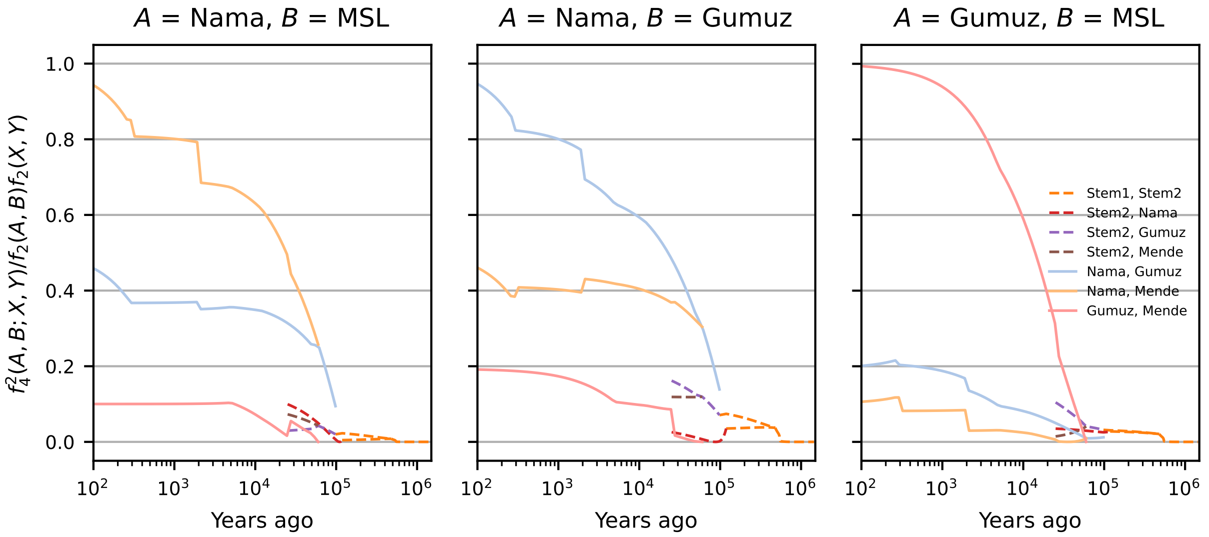

Figure S19: **Predicted f_4 between pairs of contemporary populations and ancient branches from the merger-with-stem-migration model.** $f_4^2(A, B; X, Y)/f_2(A, B)f_2(X, Y)$ is interpreted as the proportion of the amount of drift between sampled populations A and B that aligns with drift between populations X and Y, sampled in the past (see Section 5.2). Differentiation between present-day populations aligns with up to $\approx 5\%$ of the drift between stems, although some population pairs show even less shared drift.

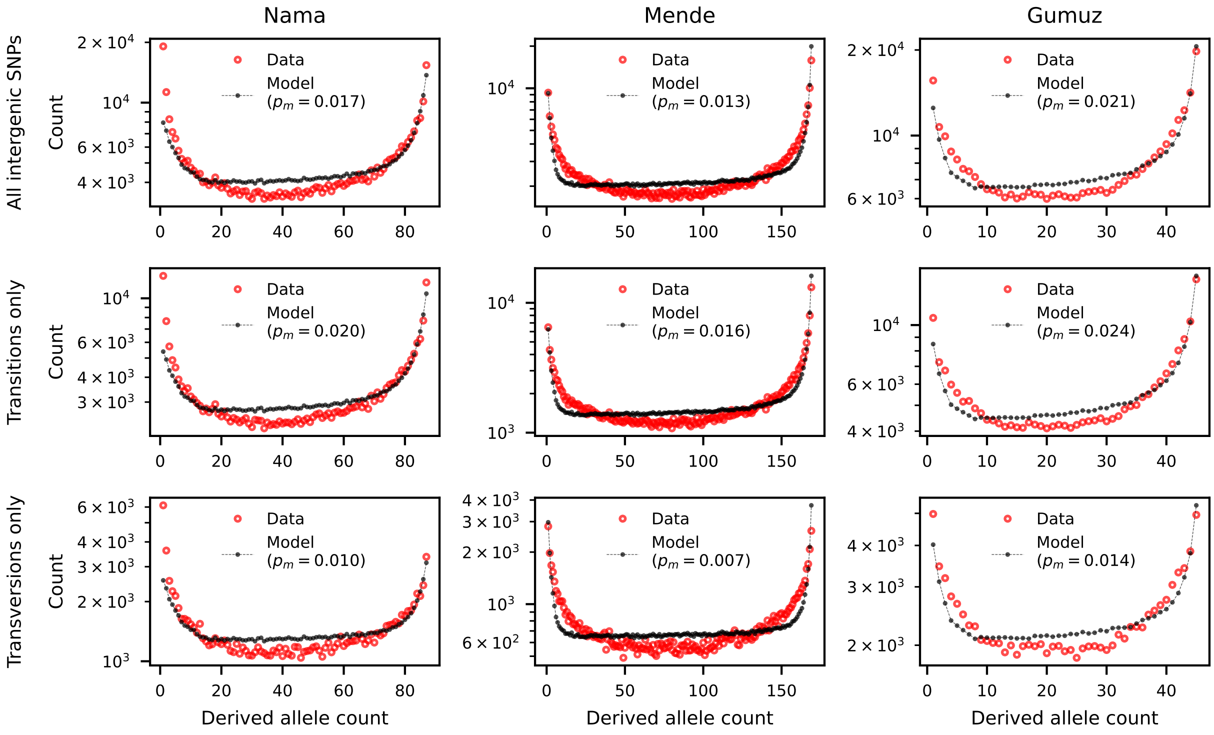

Figure S20: **Conditional SFS compared to single-origin model predictions.** The single-origin model provides a poor fit to the Neanderthal-conditional SFS in the Mende, Gumuz, and Nama (see Section 7.3.1). Ancestral state misidentification (labeled as p_m in the figure legends) was inferred to be between 1 and 2%, and misidentification rates were consistent across model comparisons. Misidentification rates of transition-type mutations were roughly double that of transversion-type mutations, consistent with the higher mutation rates of transition mutations vs. transversions.

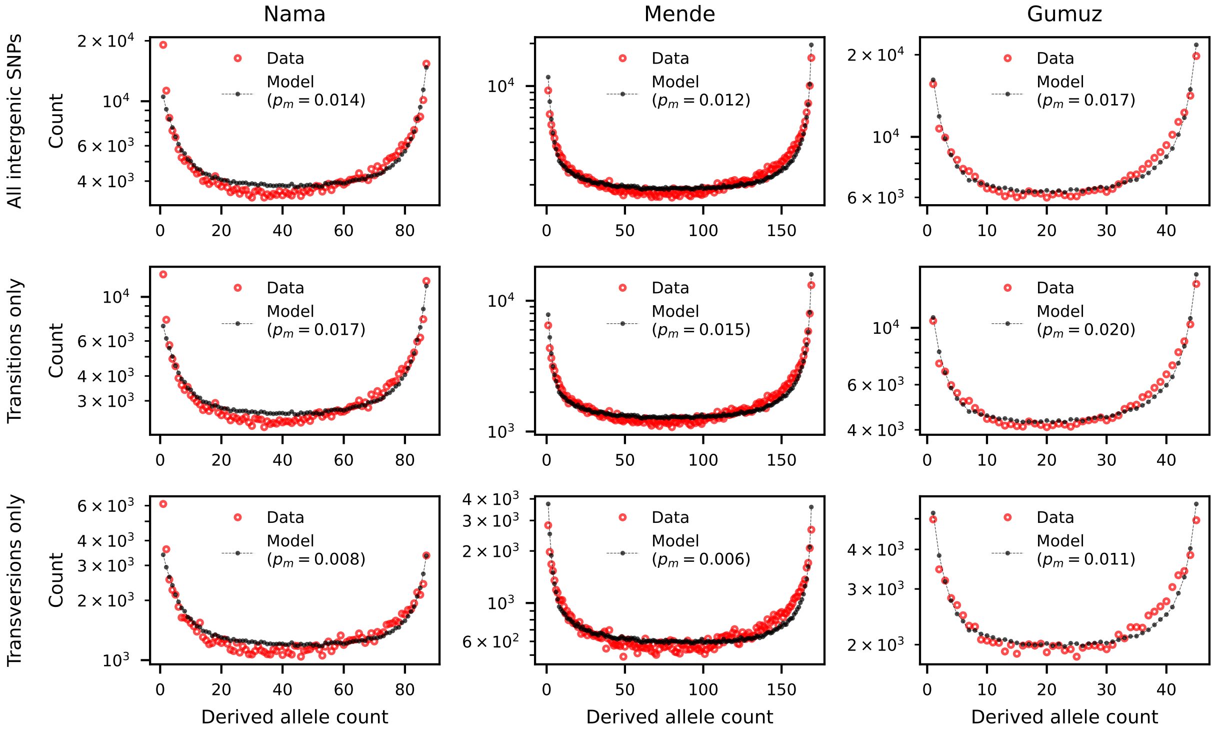

Figure S21: **Conditional SFS compared to continuous-migration model predictions.** The continuous-migration model provides a better fit to the Neanderthal-conditional SFS than either the single-origin model or the merger-without-stem-migration model, although systematic deviations are still noticeable in each population. Ancestral state misidentification (p_m), as estimated with all models, is roughly doubled for transition-type mutations compared to transversions.

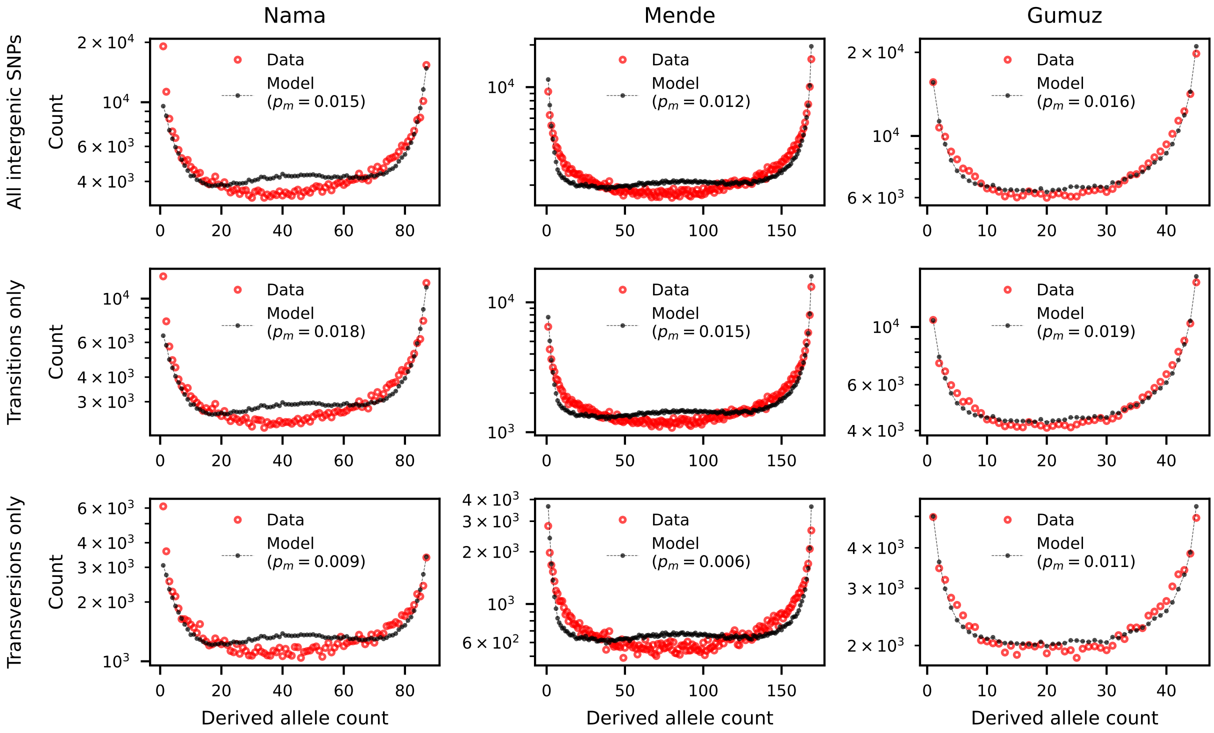

Figure S22: **Conditional SFS compared to merger-without-stem-migration model predictions.** The excess of intermediate-frequency variants in the Nama and Mende cSFS results in a poorer fit to the cSFS compared to the continuous-migration and merger-with-stem-migration models. Thus the model without stem migrations provides a poorer fit to both two-locus statistics and the cSFS. Ancestral state misidentification for transition-type mutations (p_m), as estimated with all models, is roughly double that of transversions.

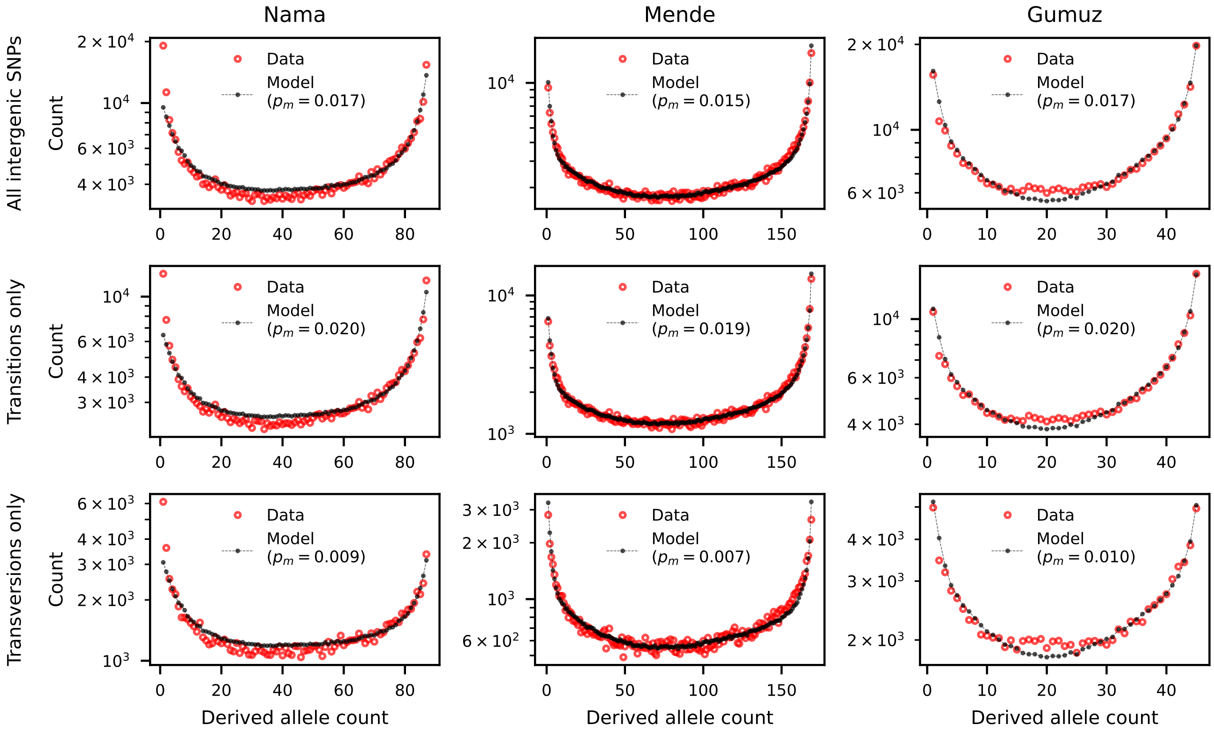

Figure S23: **Conditional SFS compared to merger-with-stem-migration model predictions.** The merger-with-stem-migration model, in addition to providing the best fit to heterozygosity and LD statistics, provided the best fit to the cSFS in the Nama, Gumuz, and Mende populations. Rates of ancestral state misidentification were consistent across each comparison, with the ancestral state of transversion mutations estimated to be $0.7-1.0\%$, and that of transition mutations estimated to be $1.9-2.0\%$. These misidentification rates are consistent with the known difference in mutation rates between transitions and transversions, as well as other SFS-based inferences in humans using the Thousand Genomes data and the 6-primate alignment (see Methods).

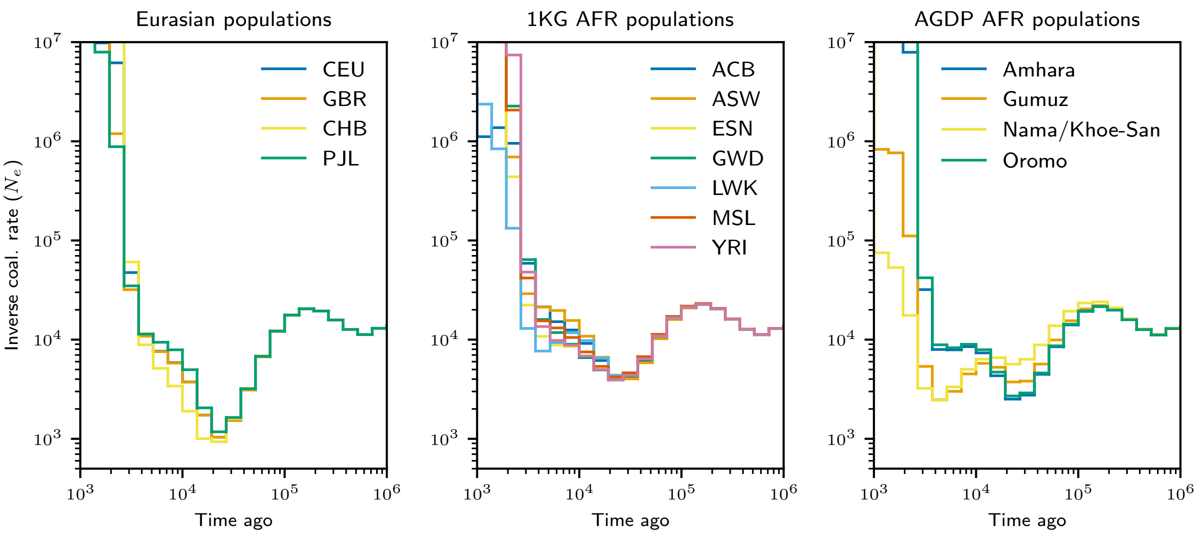

Figure S24: **Inverse instantaneous coalescence rates inferred from the data.** Using combined data from the Thousand Genomes Project and the ADRP, we reconstructed gene genealogies using Relate and calculated the inverse instantaneous coalescence rate for samples within each population. (A) Eurasian populations (CEU: European American, GBR: British, CHB: Han Chinese, PJL: Punjabi), (B) Thousand Genomes African populations (ACB: Afro-Caribbean, ASW: African American, ESN: Esan, GWD: Gambian, LWK: Luhya, MSL: Mende, YRI: Yoruba), (C) ADRP populations.

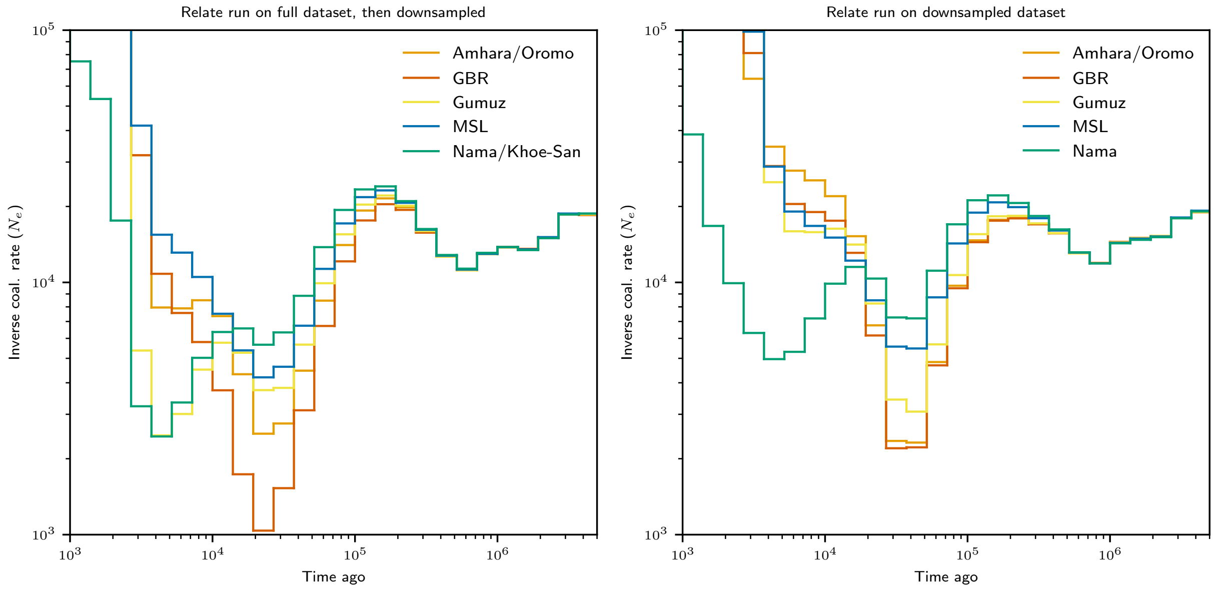

Figure S25: **Inverse instantaneous coalescence rates inferred from the data, subset to focal populations in the model inferences.** Fig. S24 shows IICR curves from reconstructed genealogies inferred using a larger dataset (including nearly 1,200 individuals) than the subset of populations we primarily focused on here. To assess the robustness of Relate-inferred genealogies and for a direct comparison to our inferred demographic models, we ran Relate on the 290 genomes from the Nama, Gumuz, Mende, Amhara/Oromo, and British individuals. While the reconstructed IICR curves are qualitatively similar to those from the full dataset for these populations in the distant past, there are noticeable discrepancies over the recent history in the last tens of thousands of years. Left: Relate run on full dataset, showing focal populations. Right: Relate run directly on the subset dataset of focal populations.

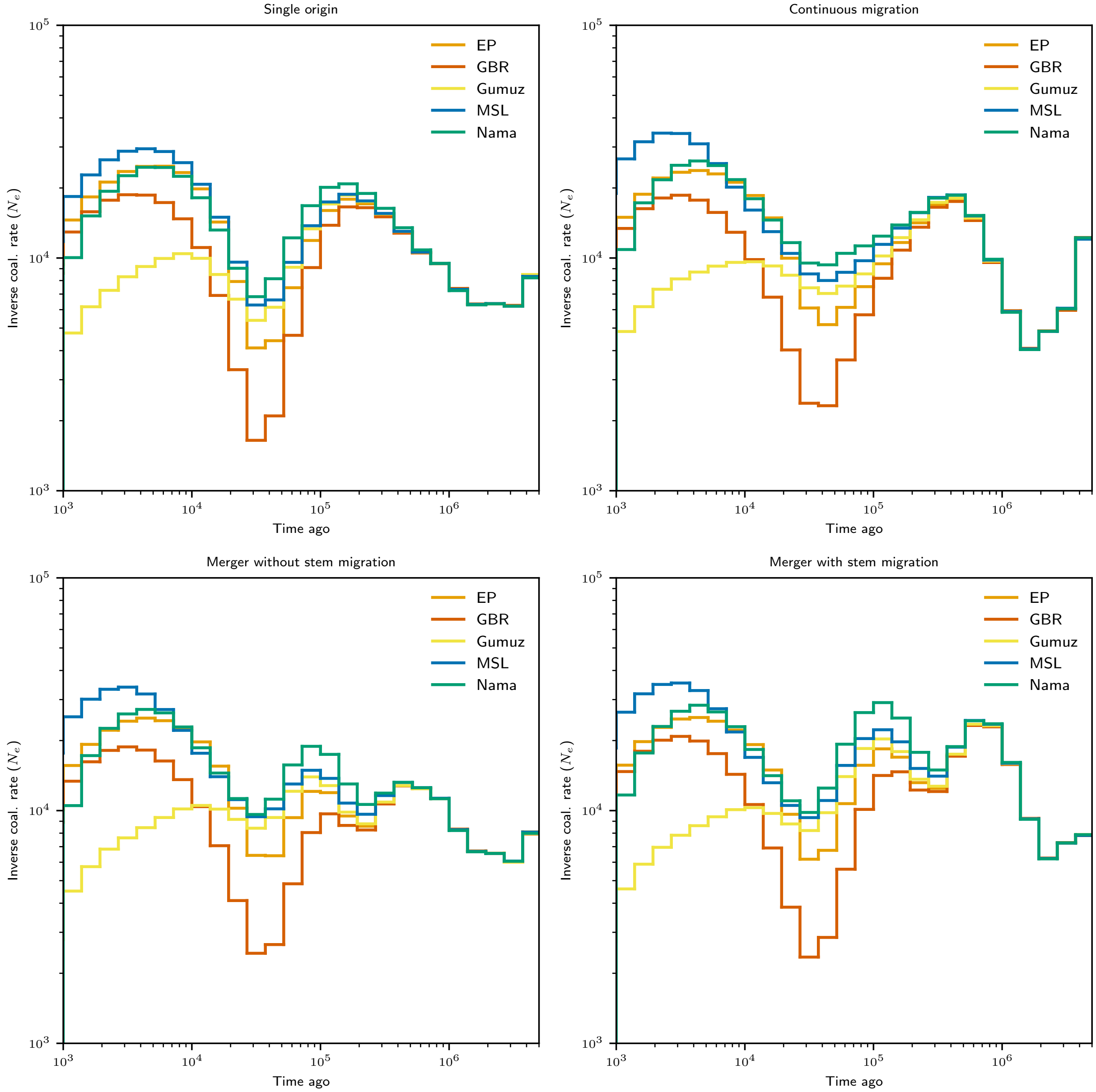

Figure S26: **Inverse instantaneous coalescence rates reinferred from simulated data.** We simulated data under our four highlighted models, sampling the same number of individuals per population as the original dataset, and then ran Relate on each simulated dataset to reconstruct IICR curves for each. Each model has qualitatively different histories, particularly for the distant past with varying numbers and timings of oscillations. However, due to the discrepancies in reconstructed genealogies using even the same data as input (Figs. S25 and S28), it is difficult to draw firm conclusions from quantitative comparisons between reconstructed genealogies.

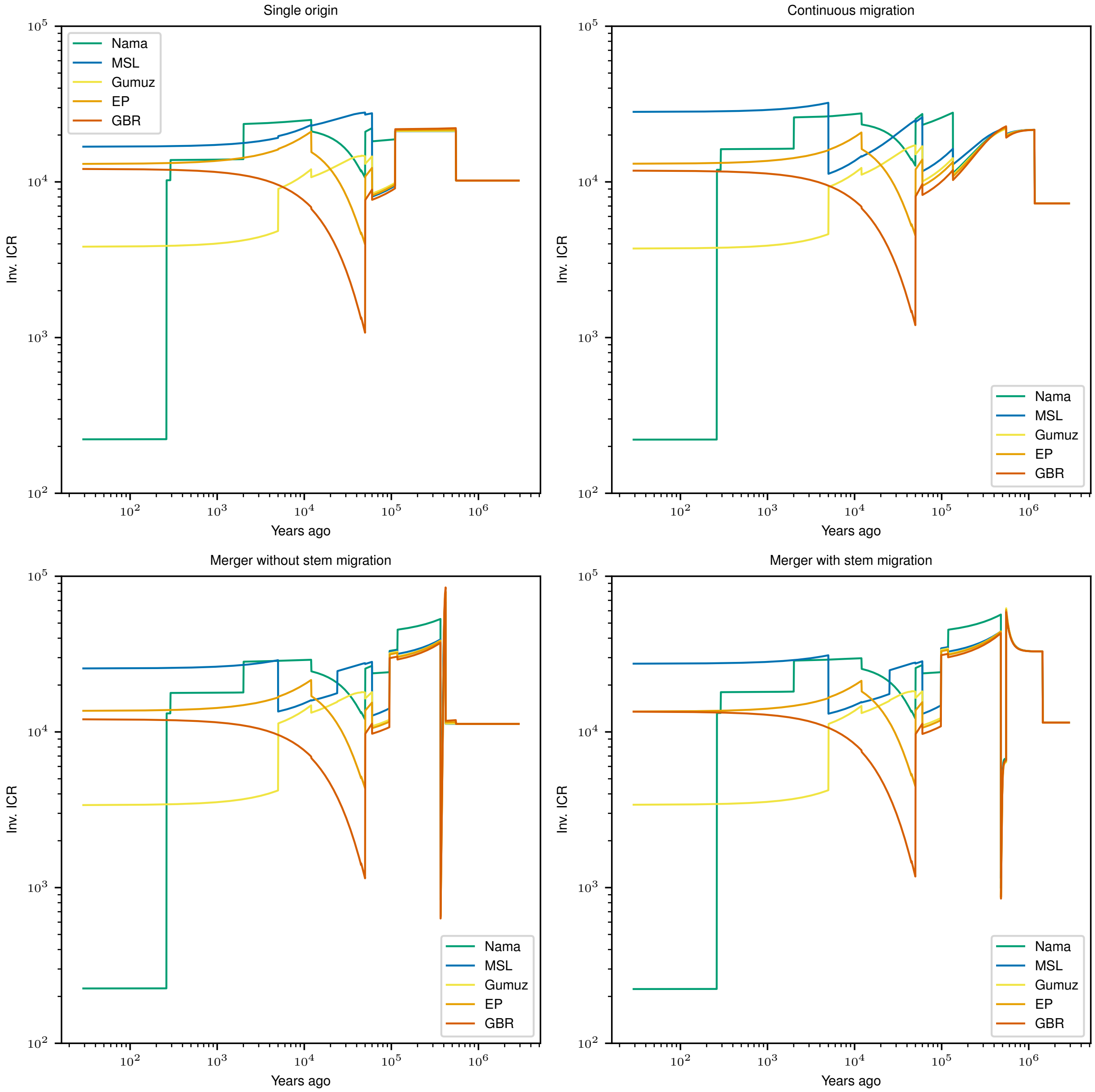

Figure S27: **Expected inverse instantaneous coalescence rates from models.** The inferred demographic models specify population sizes, split times, and migration rates and events, making it possible to compute the exact expected IICR curve for each population. Due to discrete events (instantaneous size changes, for example), the models can produce non-smooth IICR curves, although each predicts a period of increased "effective size" in the past $\sim 200 - 500$ka.

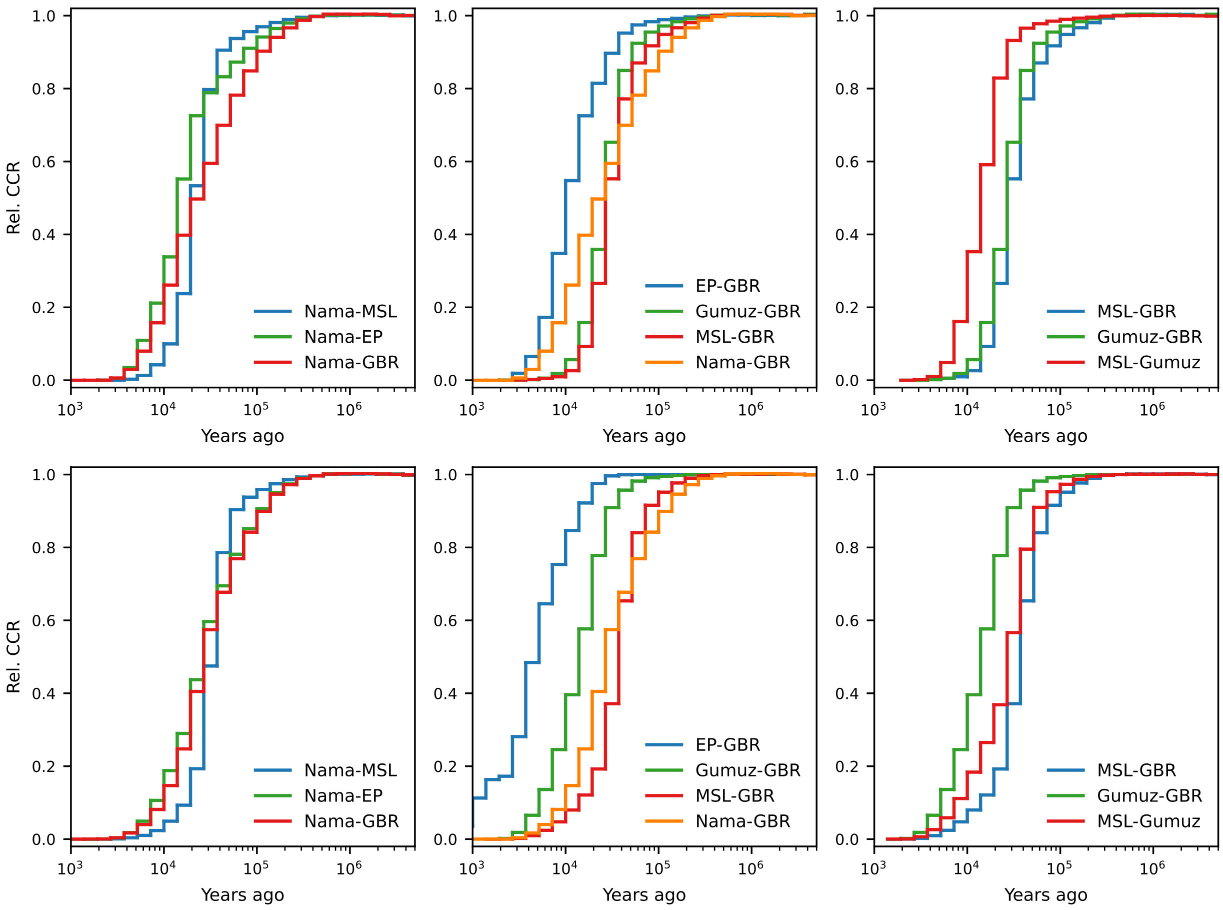

Figure S28: **Relative cross-coalescence rates inferred from the data.** Using the same reconstructed genealogies from Relate as displayed in Fig. S25, we computed relative cross-coalescence (RCCR) curves for pairs of populations using genealogies from the either the full dataset ($\sim 1,200$ individuals, top) and the subset of focal populations (290 individuals, bottom). Despite the overlap in samples, the two sets of inferred genealogies provided inconsistent RCCR, with both the timing and ordering of increased RCCR varying between the two datasets. Genealogies reconstructed from the full dataset provide a better match to each of inferred demographic models (Figs. S29–S32).

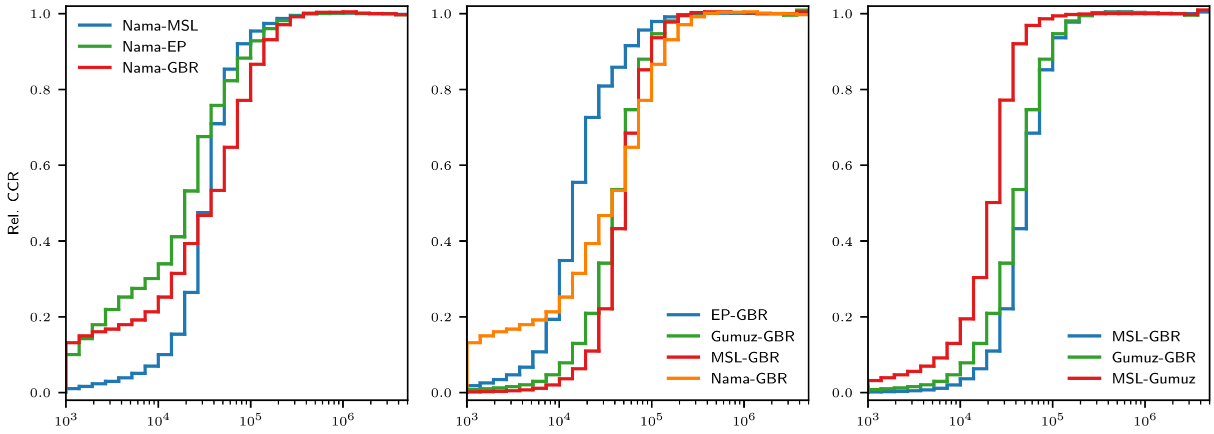

Figure S29: **Relative cross-coalescence rates reinferred from simulated data under the single-origin model.** RCCR curves from genealogies reconstructed from simulated data using Relate match those inferred from the full dataset (S28, top) for distant time points. There are large discrepancies in the very recent history, where Relate and other genome-wide coalescence-based methods are known to be underpowered.

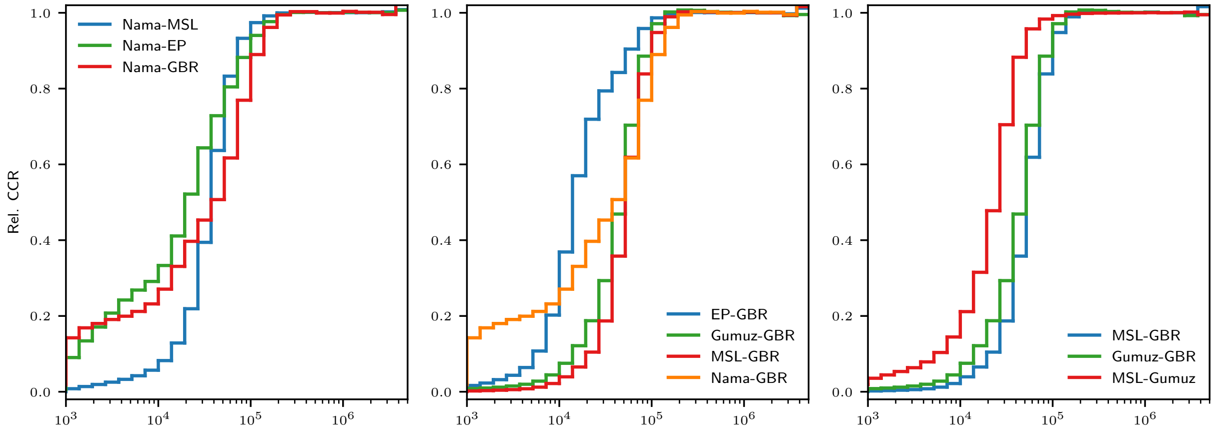

Figure S30: **Relative cross-coalescence rates reinferred from simulated data under the continuous-migration model.** RCCR curves from genealogies reconstructed from simulated data using Relate match those inferred from the full dataset (S28, top) for distant time points. There are large discrepancies in the very recent history, where Relate and other genome-wide coalescence-based methods are known to be underpowered.

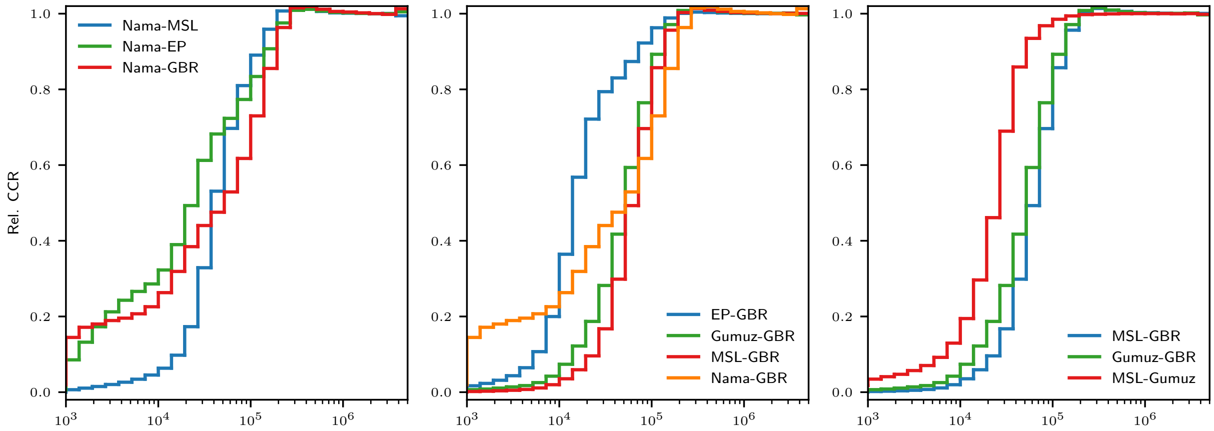

Figure S31: **Relative cross-coalescence rates reinferred from simulated data under the merger-without-stem-migration-model.** RCCR curves from genealogies reconstructed from simulated data using Relate match those inferred from the full dataset (S28, top) for distant time points. There are large discrepancies in the very recent history, where Relate and other genome-wide coalescence-based methods are known to be underpowered.

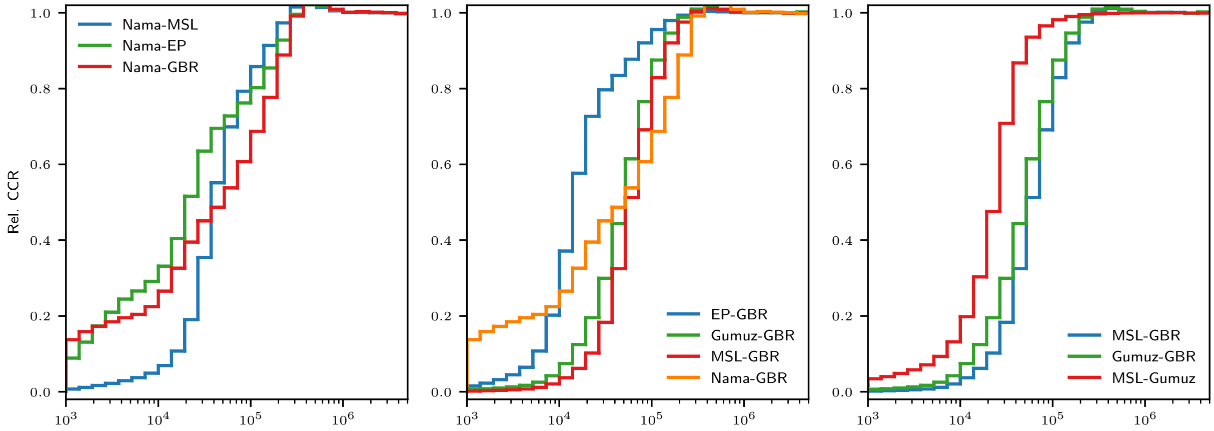

Figure S32: **Relative cross-coalescence rates reinferred from simulated data under the merger-with-stem-migration model.** RCCR curves from genealogies reconstructed from simulated data using Relate match those inferred from the full dataset (S28, top) for distant time points. There are large discrepancies in the very recent history, where Relate and other genome-wide coalescence-based methods are known to be underpowered.

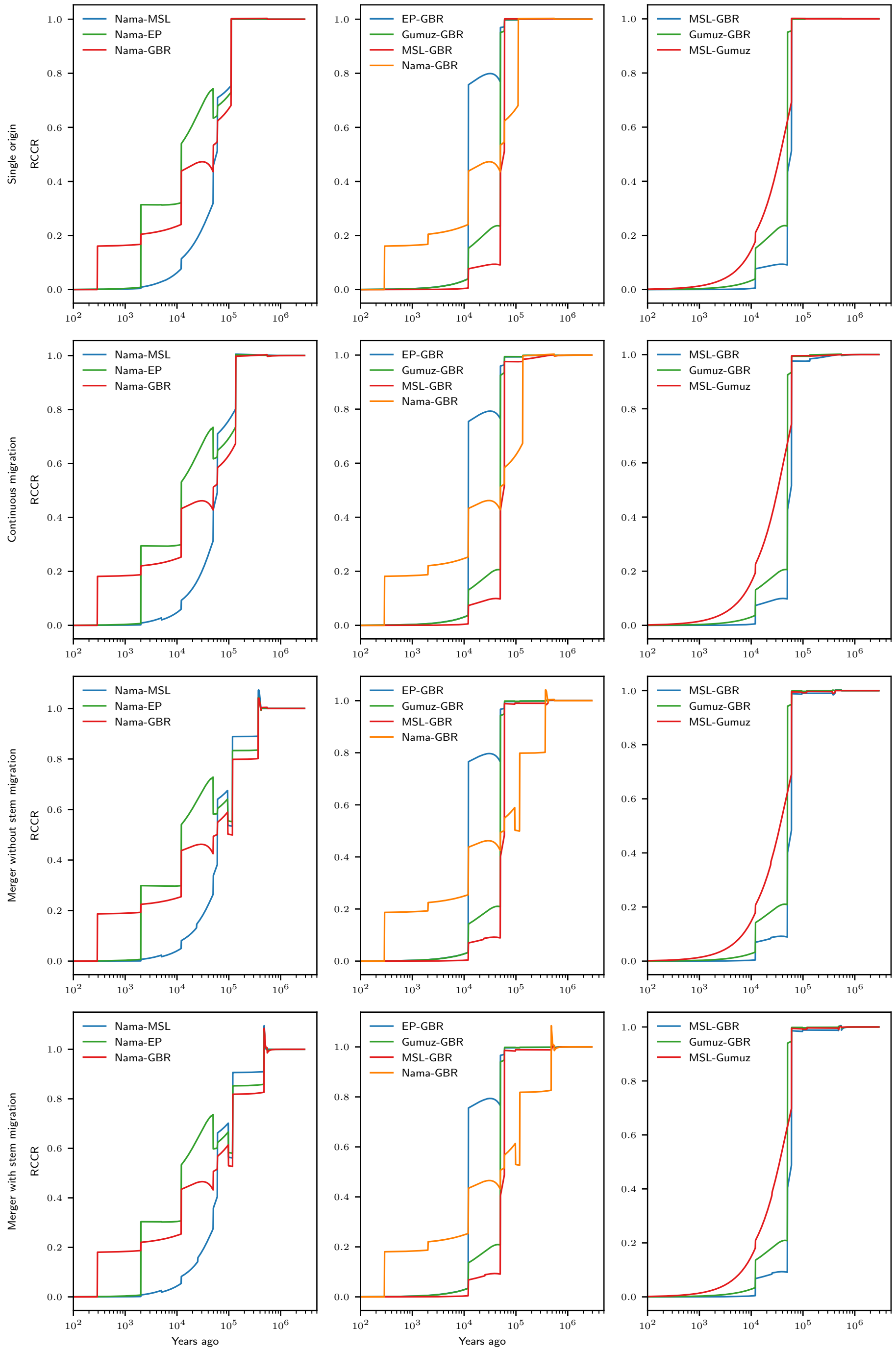

Figure S33: **Expected relative cross-coalescence rates from inferred models.** Our inferred demographic models allow for exact calculation of expected RCCR curves, showing that while the parameterizations of the four models are quite different, they each predict very similar RCCR profiles.

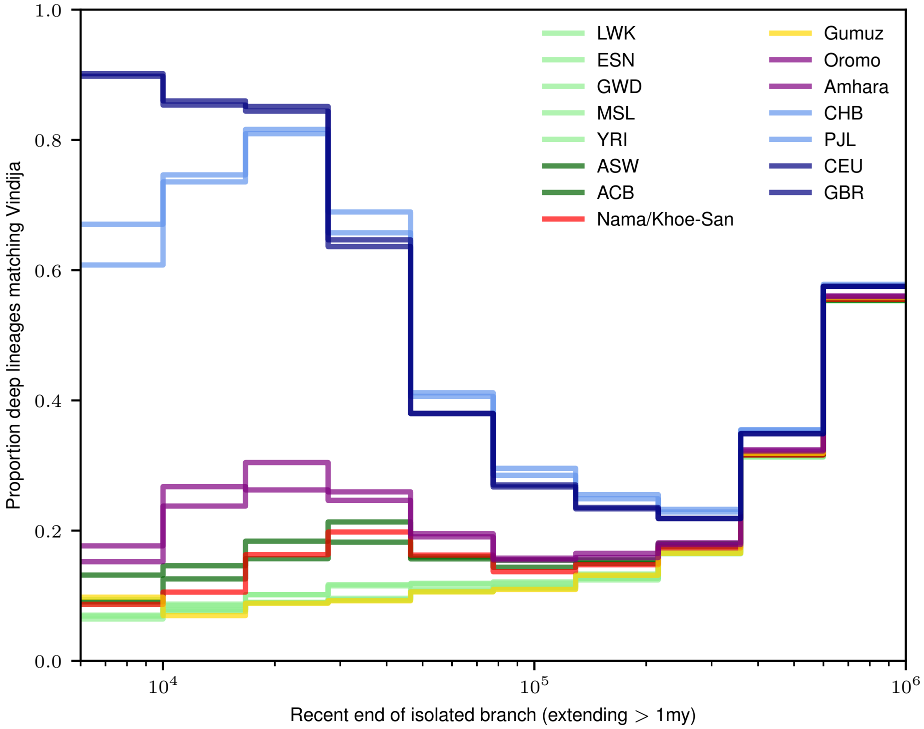

Figure S34: **Neanderthal-matching deep branch distribution from the data.** Following the approach of Speidel *et al.* (2019), we categorized Neanderthal-matching deep branches for each population from Relate-inferred gene genealogies. The proportion of deep branches with Neanderthal affinity is highest for Eurasian populations, as expected, and higher for African populations with recent Eurasian ancestry (Oromo, Amhara, ACB, ASW, and Nama) than the Gumuz and West African populations.

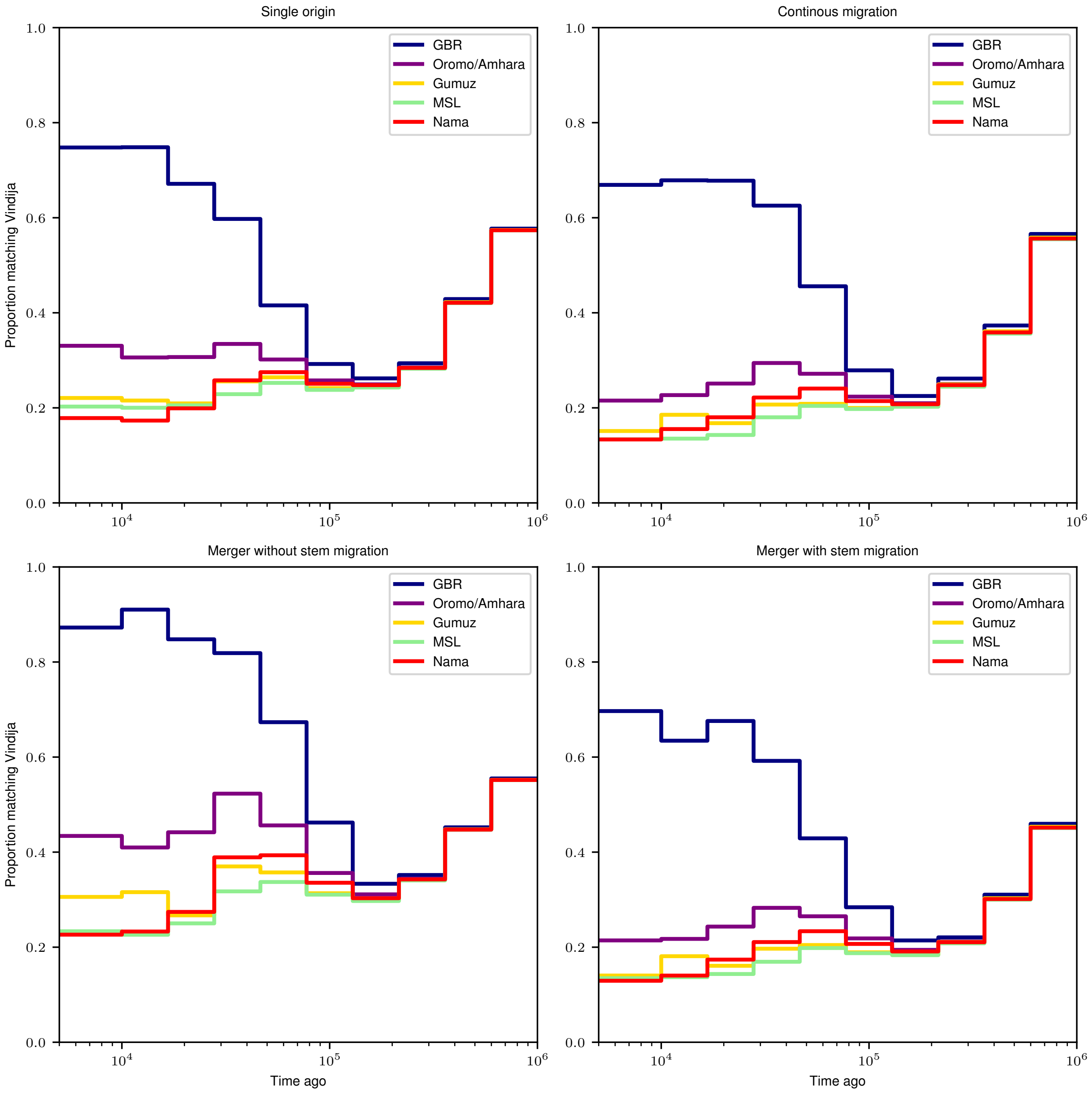

Figure S35: **Deep branch distribution reinferred from simulated data.** We used Relate to reinfer gene genealogies from simulated data under the four highlighted models and categorized Neanderthal-matching deep branches in the simulated datasets. Each model provided a qualitative match to the data (highest proportion of Neanderthal-matching deep branches in the British, followed by Amhara/Oromo and Nama). Similar to reinferred IIRC and RCCR, no model provided a perfect match to the reconstructions using Relate with the data, but the similarities between reconstructed genealogies in the simulated data suggest such coalescence-rate based curves are underpowered to differentiate between plausible models of history.

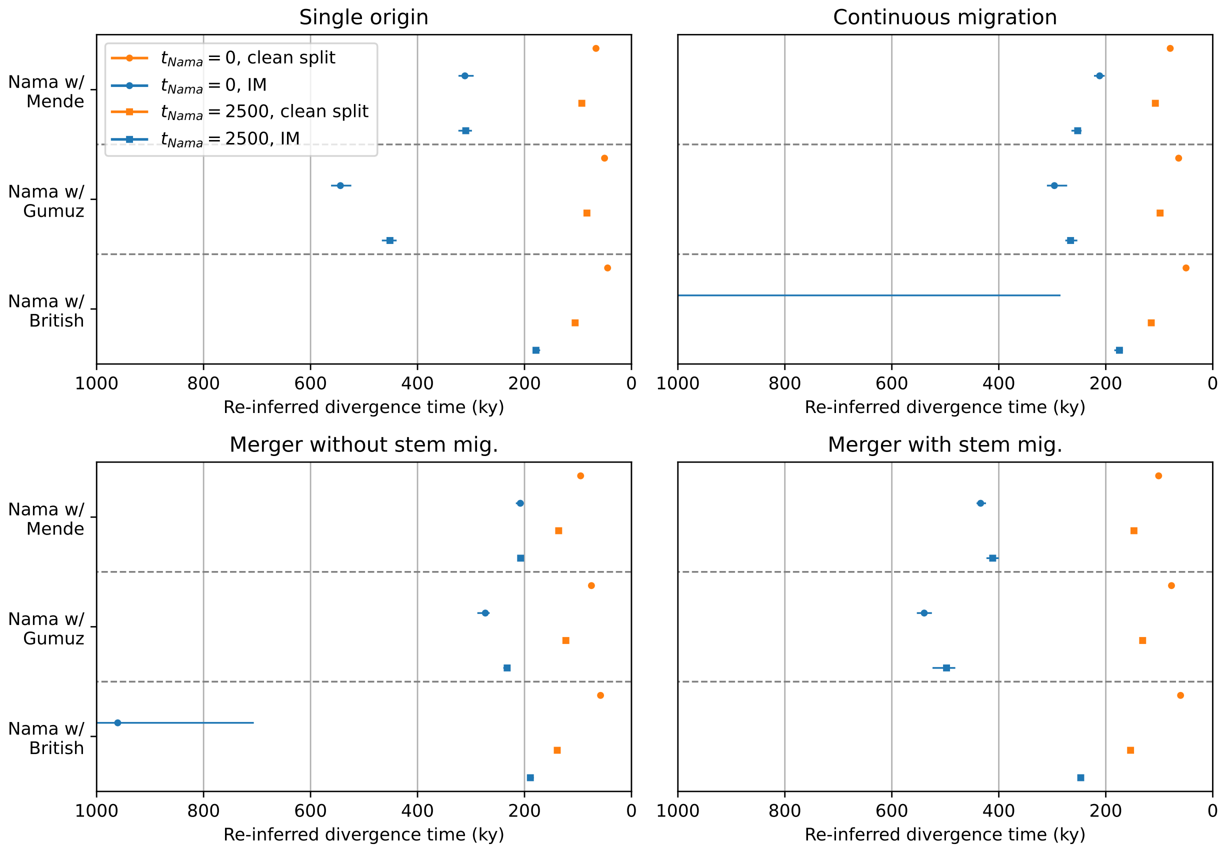

Figure S36: **IM model split times reinferred from simulated data of full model.** We simulated 10 diploid individuals under the four highlighted models (Sec. 7.4). Using the joint-SFS, we reinferred a simpler isolation-with-migration (IM) model for pairs of populations including the Nama to explore the effect of model mis-specification on the earliest inferred divergence times between Khoe-San and other populations. We simulated data with Nama individuals sampled at present ($t = 0$) as well as Nama individuals sampled 2,500 years ago, prior to the recent gene flow from East Africans and Europeans. We fit two models for each model and population pair: one that allowed for migration between the populations (IM), and one that disallowed migration (clean split). Despite all original models having recent population divergences $\sim$ 120ka, the reinferred IM models all settled on divergence times greater than 200ka and sometimes much greater. If the true historical model includes reticulation or long-lasting structure, simpler IM models may be severely biased in favor of more ancient divergence times.

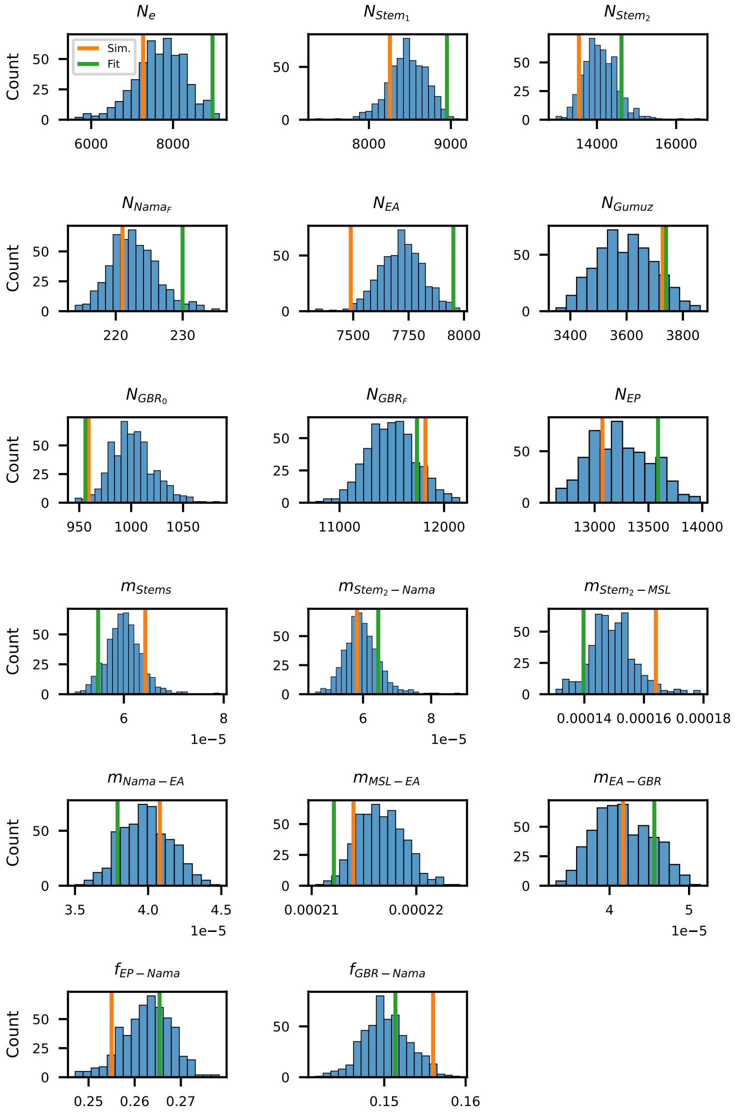

Figure S37: **Evaluation of confidence intervals in the continuous migration model.** From 500 replicate bootstrap datasets, the true (simulated) parameter value lies within the distribution of inferred values from the bootstrap datasets. Orange line: simulated parameter value. Green line: inferred parameter value. Blue histogram: inferred parameter values from 500 bootstrap replicate datasets.

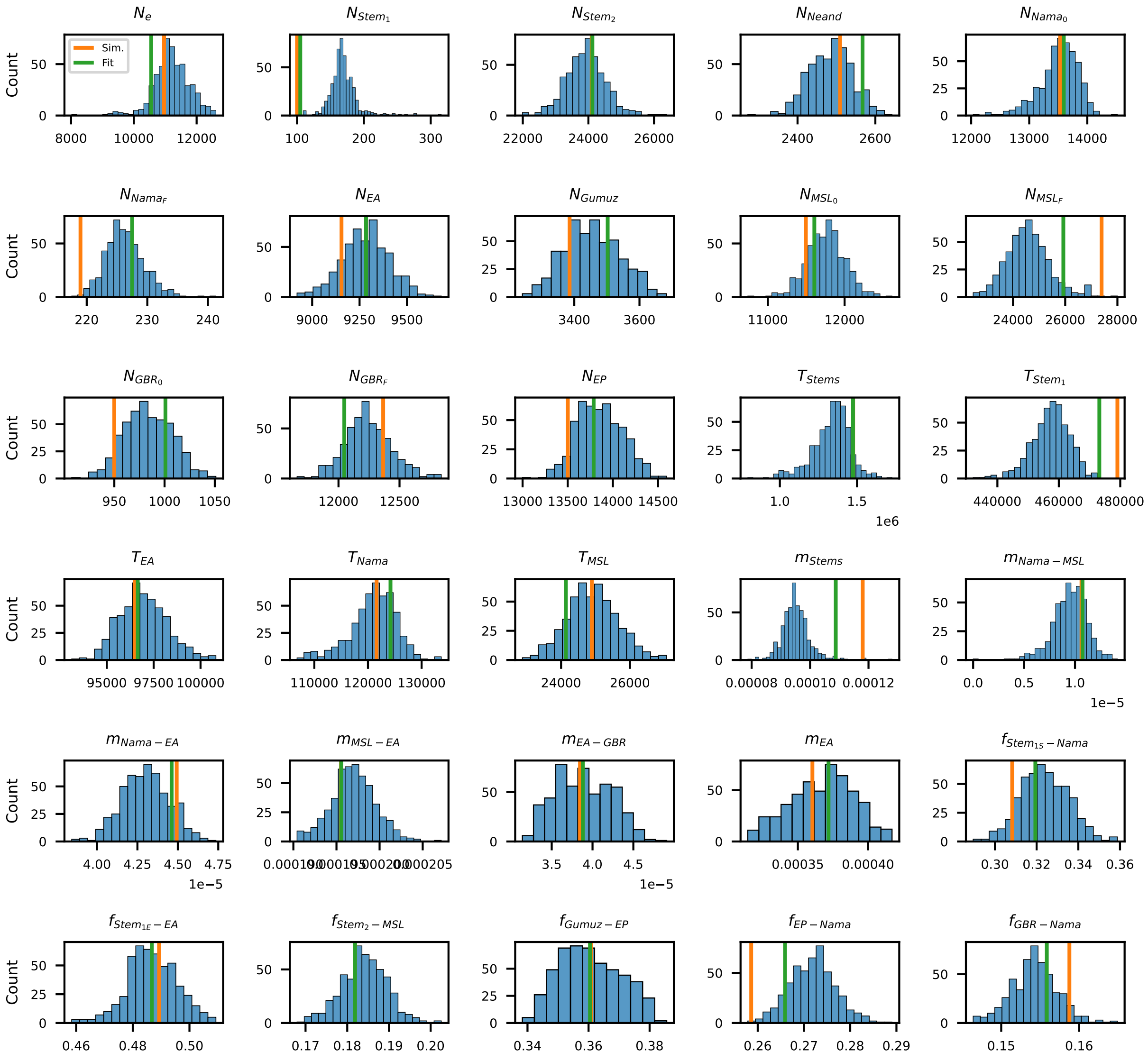

Figure S38: **Evaluation of confidence intervals in the merger with stem migration model.** From 500 replicate bootstrap datasets, the true (simulated) parameter value typically lies within the distribution of inferred values from the bootstrap datasets. Stem 1 experienced a strong bottleneck, at the lower bound of allowed inferred values. In this case, and other parameters directly related to Stem 1, bootstrap-inferred values do not overlap with the true value, although this may be due to incomplete convergence of the optimization at these bounds of parameter space. Orange line: simulated parameter value. Green line: inferred parameter value. Blue histogram: inferred parameter values from 500 bootstrap replicate datasets.

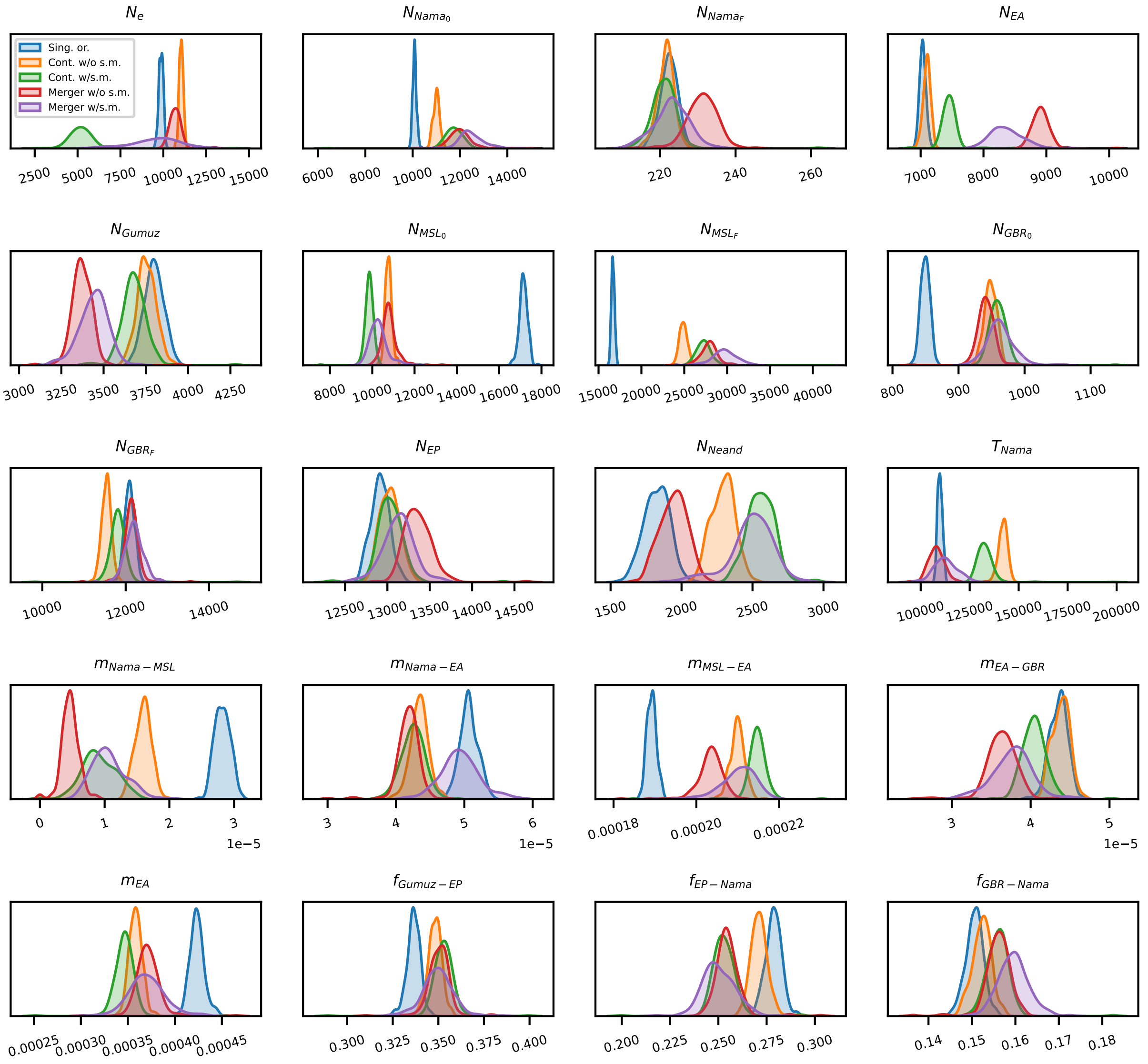

Figure S39: **Overlap of bootstrap distributions of inferred parameters across multiple parameterized models.** While the details of deep history differ substantially between the different inferred models, the parameterization of recent history (population sizes, migration rates) are similar. Despite the differences in the deep history, many parameters related to recent history are consistent across all tested models. The single origin model (blue) more often has inferred values distinct from other models, although this model provides a far worse fit to the data than the other models.

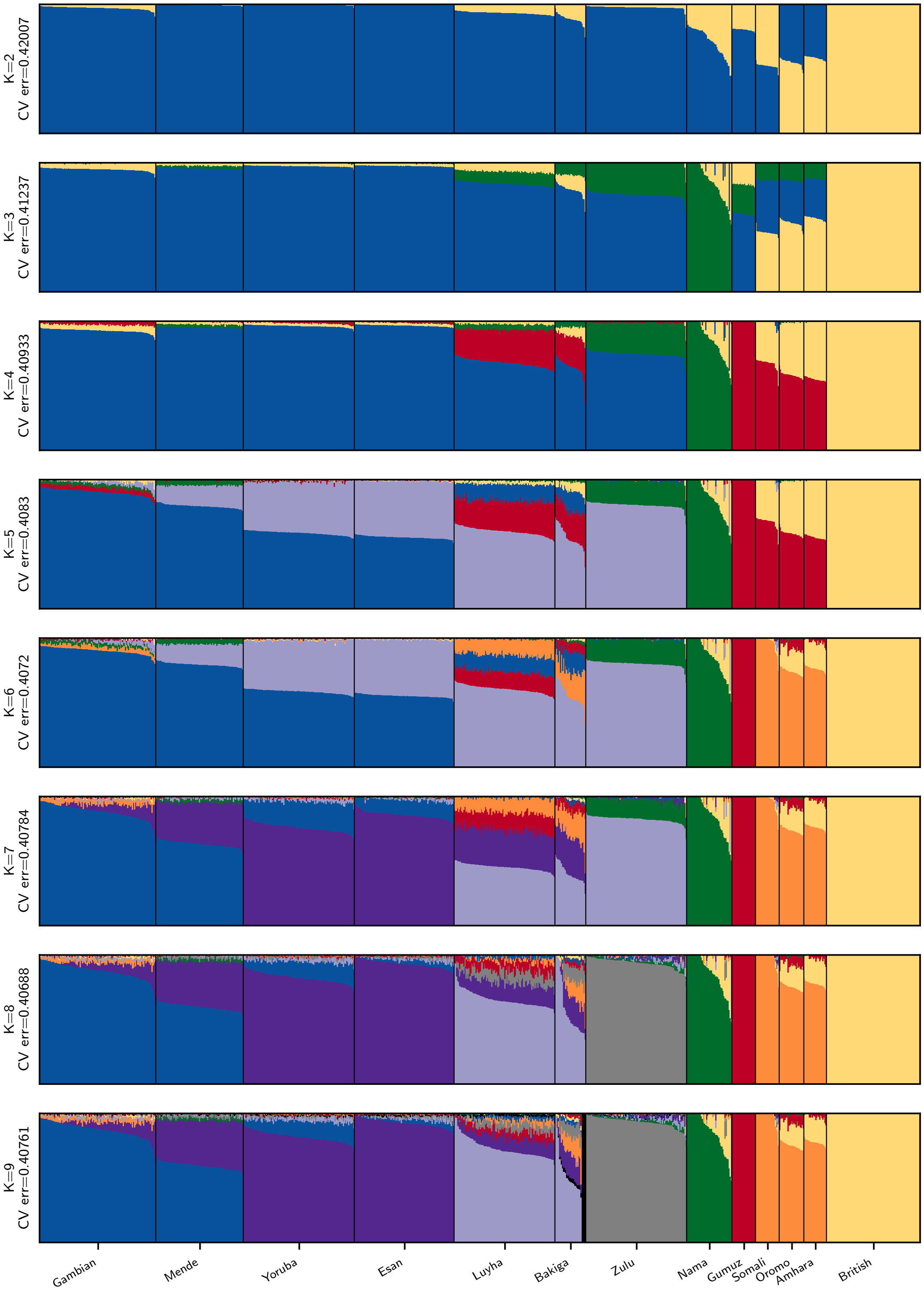

Figure S40: `ADMIXTURE` **clustering using** $K = 2$ **to** 9 **clusters.** `ADMIXTURE` (Alexander *et al.*, 2009) was used to cluster all individuals in the subset of populations used in analyses in this paper. At $K = 5$ clusters, West African populations begin to show variable separation in assigned clusters, likely due to isolation-by-distance demographic processes rather than recent admixture events. Figure 2D in the main text shows the `ADMIXTURE` results for $K = 4$, which most clearly displays known admixture events in the histories of East and South African populations.
