# Supplementary material for: A weakly structured stem for human origins in Africa
Source: Nature. 2023 May 17;617(7962):755–63. doi: 10.1038/s41586-023-06055-y (PMC10208968; doi:10.1038/s41586-023-06055-y)
Supplement: Supplementary file 2 — Reporting Summary [file 41586_2023_6055_MOESM2_ESM.pdf]

nature portfolio

Corresponding author(s): Brenna Henn, Simon Gravel

Last updated by author(s): Jan 18, 2023

Reporting Summary

Nature Portfolio wishes to improve the reproducibility of the work that we publish. This form provides structure for consistency and transparency in reporting. For further information on Nature Portfolio policies, see our Editorial Policies and the Editorial Policy Checklist.

Statistics

For all statistical analyses, confirm that the following items are present in the figure legend, table legend, main text, or Methods section.

n/a	Confirmed	
☒	☐	The exact sample size (*n*) for each experimental group/condition, given as a discrete number and unit of measurement
☒	☐	A statement on whether measurements were taken from distinct samples or whether the same sample was measured repeatedly
☐	☒	The statistical test(s) used AND whether they are one- or two-sided<br>*Only common tests should be described solely by name; describe more complex techniques in the Methods section.*
☒	☐	A description of all covariates tested
☐	☒	A description of any assumptions or corrections, such as tests of normality and adjustment for multiple comparisons
☐	☒	A full description of the statistical parameters including central tendency (e.g. means) or other basic estimates (e.g. regression coefficient) AND variation (e.g. standard deviation) or associated estimates of uncertainty (e.g. confidence intervals)
☒	☐	For null hypothesis testing, the test statistic (e.g. *F*, *t*, *r*) with confidence intervals, effect sizes, degrees of freedom and *P* value noted<br>*Give P values as exact values whenever suitable.*
☒	☐	For Bayesian analysis, information on the choice of priors and Markov chain Monte Carlo settings
☒	☐	For hierarchical and complex designs, identification of the appropriate level for tests and full reporting of outcomes
☒	☐	Estimates of effect sizes (e.g. Cohen's *d*, Pearson's *r*), indicating how they were calculated

Our web collection on statistics for biologists contains articles on many of the points above.

Software and code

Policy information about availability of computer code

Data collection	No software was used to collect data for this paper. Software used to generate variant calls are described elsewhere in van Eeden et al (Genome Biology, 2022) and Gurdasani et al (Nature, 2015).
Data analysis	Code for the analysis software used in this paper is found at the following locations:<br>moments-LD (https://bitbucket.org/simongravel/moments),<br>Demes (https://github.com/popsim-consortium/demes-python),<br>Relate (https://myersgroup.github.io/relate/),<br>msprime (https://github.com/tskit-dev/msprime),<br>tskit (https://github.com/tskit-dev/tskit),<br>and custom scripts implementing analyses using each of these software are available at https://github.com/apragsdale/african-structure-paper.

For manuscripts utilizing custom algorithms or software that are central to the research but not yet described in published literature, software must be made available to editors and reviewers. We strongly encourage code deposition in a community repository (e.g. GitHub). See the Nature Portfolio guidelines for submitting code & software for further information.

Data

Policy information about availability of data

All manuscripts must include a data availability statement. This statement should provide the following information, where applicable:

- Accession codes, unique identifiers, or web links for publicly available datasets
- A description of any restrictions on data availability
- For clinical datasets or third party data, please ensure that the statement adheres to our policy

Nama sequencing data are available from the European Genome-Phenome Archive (EGA), accession number: EGAD00001006198. The African Diversity Reference Panel can be found at accession: EGAS00001000960.

Human research participants

Policy information about studies involving human research participants and Sex and Gender in Research.

Reporting on sex and gender	Adults over the age of 18 were invited to participate in the study. No preference was stated for male or female participants, and no individual was excluded on the basis of their sex. Results apply equally to both sexes.
Population characteristics	DNA samples were collected from three Nama communities in the Richtersveld region of South Africa, which borders southern Namibia. Members of the Nama community in the Richtersveld were initially approached regarding a genetic ancestry study in 2011. The Nama are a Khoekhoe speaking population who traditionally subsisted by sheep, goat and cattle pastoralism. Today, members of the community continue to be involved in pastoralism, as well as local mining, and tourism wage labor. All individuals self-identifying as Nama, Nama-Dama, or Coloured were encouraged to participate. No individual was excluded on the basis of ethnicity during recruitment.
Recruitment	Collection primarily occurred at home, by first approaching a family member, gauging interest in the study, entering the home or sitting outside by their invitation, oral and written consent occurring with family members present and then finally – completing the DNA sampling. By sampling individuals at home, members of the family are able to voice concerns, whether or not they decide to participate, and thus the final decisions are made in a slow and ethical manner. This process sometimes involved introducing the study and then returning at a later day in order to give participants sufficient time to consider. Families were approached based on a research assistant's determination that the family would be interested in participating, or via community meetings.
Ethics oversight	Written consent was recorded per our IRB protocol with human subjects approval from Stanford University (Protocol #13829), Stellenbosch University (N11/07/210) and later maintained via SUNY Stony Brook (Protocol #727494).

Note that full information on the approval of the study protocol must also be provided in the manuscript.

Field-specific reporting

Please select the one below that is the best fit for your research. If you are not sure, read the appropriate sections before making your selection.

☐ Life sciences ☐ Behavioural & social sciences ☒ Ecological, evolutionary & environmental sciences

For a reference copy of the document with all sections, see nature.com/documents/nr-reporting-summary-flat.pdf

Ecological, evolutionary & environmental sciences study design

All studies must disclose on these points even when the disclosure is negative.

Study description	We use low coverage whole genomes from several human populations to estimate demographic parameters associated with long-term evolutionary history. Such parameters include effective population size, divergence time, gene flow and population growth. The focus of the study is understanding the extent to which population structure exists in African populations during the origin of Homo sapiens.
Research sample	Low coverage (4-8x) Illumina short read data were generated for the Nama, Gumuz, Amhara and Oromo populations as part of the African Diversity Reference Panel (Sanger / Wellcome Trust). Data were jointly called with 1000 Genomes Project, and the GBR, YRI, MSL, TSI samples were parsed for various comparative analyses.
Sampling strategy	A sample size of ~100 was estimated for initial DNA collection in the Nama with the aim of capturing heterogeneity in ancestry across multiple villages and accounting for potential family members. For moments.LD analysis, we required a population to have a minimum of 25 individuals in order to capture variants at 2% or greater.
Data collection	Demographic and DNA collection was initiated in 2012 with a joint team of South African and American researchers which included both geneticists and anthropologists (BMH, MM, along with Christopher Gignoux, Caitlin Uren, Justin Myrick and Cedric Werely).

	Research assistants were fluent in Afrikaans, Nama and English (Hendrik Kaimann and Willem DeKlerk). Demographic data were recorded by pen and paper, then later transcribed into an Excel sheet (BMH, JWM).
Timing and spatial scale	N/A
Data exclusions	22 samples were excluded due to potential cross-contamination as detected by FREEMIX.
Reproducibility	N/A
Randomization	N/A
Blinding	N/A

Did the study involve field work? ☒ Yes ☐ No

Field work, collection and transport

Field conditions	N/A
Location	Richtersveld, South Africa
Access & import/export	Data were exported with permission from the Department of Health, Republic of South Africa. Permit number: J1/2/4/2 NO 1/13
Disturbance	N/A

Reporting for specific materials, systems and methods

We require information from authors about some types of materials, experimental systems and methods used in many studies. Here, indicate whether each material, system or method listed is relevant to your study. If you are not sure if a list item applies to your research, read the appropriate section before selecting a response.

Materials & experimental systems

n/a	Involved in the study	
☒	☐	Antibodies
☒	☐	Eukaryotic cell lines
☒	☐	Palaeontology and archaeology
☒	☐	Animals and other organisms
☒	☐	Clinical data
☒	☐	Dual use research of concern

Methods

n/a	Involved in the study	
☒	☐	ChIP-seq
☒	☐	Flow cytometry
☒	☐	MRI-based neuroimaging
